# Participant experiences and recommendations for psychedelic clinical trials and future treatment delivery: a qualitative study with experts by experience from the Psychedelic Participant Advocacy Network

**DOI:** 10.3389/fpsyt.2026.1682810

**Published:** 2026-10-14

**Authors:** Mathieu Seynaeve, Leonie Schneider, Ian Roullier, Katharine Lazenby, Caroline Lilley, Sophia Lobo, Anna O. Ermakova, Hattie Wells, Anthony Cleare, James Rucker

**Affiliations:** 1 Beckley Psytech Ltd., Oxford, United Kingdom; 2 Department of Psychological Medicine, Institute of Psychiatry, Psychology & Neuroscience, King’s College London, London, United Kingdom; 3 Psychedelic Participant Advocacy Network (PsyPAN), London, United Kingdom

**Keywords:** clinical trials, DMT, drug approval pathways, participant perspectives, psilocybin, psychedelics, major depressive disorder, eating disorders

## Abstract

**Background:**

While psychedelic treatments for difficult-to-treat mental health conditions are advancing, participant perspectives remain under-explored. This study examined former participants’ experiences across recruitment, preparation, dosing, integration and study ending, and generated recommendations for future trials and treatment delivery.

**Methods:**

This qualitative descriptive study, informed by reflexive thematic analysis, included four Psychedelic Participant Advocacy Network (PsyPAN) members who were former psychedelic-trial participants. They completed three remote focus groups on recruitment and screening, preparation and dosing, and post-dose integration and the post-study period. Three had joined treatment-resistant depression studies involving psilocybin or N,N-dimethyltryptamine; one had joined a psilocybin study for an eating disorder. A one-day workshop reviewed the analysis and developed recommendations, refined with wider PsyPAN input.

**Results:**

Five themes were developed. First, participants entered trials seeking potentially effective treatment but encountered procedures shaped by eligibility criteria, equipoise, randomization, blinding, standardization, fixed schedules and endpoint-driven evidence generation. Second, participants’ accounts extended “set and setting” to the clinical-trial context by showing how research-specific conditions, including treatment-allocation uncertainty, communication, staff conduct, time-limited support, study ending and the wider support matrix, shaped experience and could mitigate tensions between treatment-seeking and research requirements. Third, facilitators mediated between protocol requirements and participants’ embodied, relational and psychological needs, particularly during dosing. Fourth, dosing was experienced as the beginning of an ongoing process of meaning-making, change and integration. Fifth, experiences after study ending varied; some participants described loss, grief or frustration, while all reported that further structured support would have been beneficial. They highlighted limited access to psychedelically informed care and advocated independent, trial-specific peer support. Recommendations emphasized clear communication, expectation management, distinction between research and treatment frames, reduced avoidable burden, staff continuity, participant agency, boundaries around touch and movement, involvement of support networks, and planning for integration and study ending.

**Conclusion:**

Relational, contextual and systemic factors shaped participants’ experiences across recruitment, preparation, dosing, integration and study ending. Participant-informed practices should be considered when designing and delivering psychedelic studies, while recognizing scientific, regulatory and resource constraints. Structured post-study peer support may address continuing support needs after trial completion.

## Introduction

Mental health disorders affect more than one billion people globally, with depression and anxiety being the second most common source of long-term disability. Eating disorders are less common, affecting about 16 million people, but are associated with substantial impairment and premature death from medical complications or suicide (1, 2). Estimates suggest that only 9% globally, and one-third of people with major depressive disorder (MDD) in high-income countries receive minimally adequate treatment, and that about half of the patients with MDD do not seek treatment (3, 4). A comparable gap has been identified for eating disorders (5, 6).

In response to this unmet need, psychedelic treatments have re-emerged as investigational options for difficult-to-treat psychiatric disorders, including major depressive disorder (MDD), treatment-resistant depression (TRD), generalized anxiety disorder (GAD), postpartum depression, eating disorders, substance use disorders, and adjustment disorders secondary to serious medical illness (7–9). Over the past decade, clinical development has moved from small proof-of-concept studies into multi-site phase 2 and phase 3 programs, particularly in depressive disorders, meaning psychedelic treatments could receive regulatory approval in the coming years, provided that rigorous evidence on efficacy, safety and implementation can be presented to regulators (7, 10).

Classical serotonergic psychedelics, including psilocybin, lysergic acid diethylamide (LSD), N,N-dimethyltryptamine (DMT) and 5-methoxy-N,N-dimethyltryptamine (5-MeO-DMT), act primarily through serotonergic receptor agonism, and produce marked but time-limited alterations in perception, affect, cognition, and self-experience (11–13). In contemporary research, psychedelic substances are typically delivered within a structured treatment model comprising preparation, monitored dosing, and post-session integration or psychological support, reflecting the recognized importance of extra-pharmacological context (14–20).

These factors are often referred to as “set and setting,” indicating a range of psychological and environmental factors that might influence participants’ individual responses to receiving a psychedelic substance and how they make meaning of their experience (21–23). Set refers to the participant’s internal state, including personality, intentions, expectations, fears, motivations, prior experiences and preparation for the session, whereas setting refers to the physical, social and cultural environment in which the drug is administered, including the treatment room, study procedures, and the behavior of facilitators and other staff (21, 22, 24). A third factor, the “matrix,” has historically been added to denote the broader environment from which the participant comes, lives during treatment, and returns to afterward, including family relationships, living circumstances, community support and the everyday social world (21, 23, 25, 26). Recently, experts recommended more transparency about contextual factors when reporting psychedelic studies (27).

Some of these factors, which are likely at play in any psychiatric intervention (28), can be influenced to varying degrees by the support model and treatment setting used in psychedelic studies (14–17, 29, 30). Others, including developmental history, attachment style, socioeconomic background, previous healthcare experiences, social support, and the availability of post-trial healthcare, may be more difficult to affect through trial protocols. Inadequate response to previous treatments, a prerequisite for eligibility in most psychedelic studies, is often associated with negative emotions toward medication and healthcare professionals, including feelings of frustration, hopelessness, and dissatisfaction (18–20). In addition, adverse childhood experiences (ACEs), common in the difficult-to-treat psychiatric populations for which psychedelic interventions are being studied (31–34), are associated with lack of trust in medical professionals, reduced willingness to follow guidance and general relationship difficulties (35, 36).

Within the constraints of what can be influenced as part of the protocolized studies, the therapeutic alliance between participants and facilitators has been proposed as a central process to modulate acute psychedelic effects and later clinical outcomes (37–40), although direct contributions to long-term efficacy have been contested (41). Similar to the link between working alliance (42) and therapeutic benefit observed in psychotherapy (43, 44), a predictable, collaborative and boundaried relationship with facilitators may contribute to creating the conditions under which participants feel able to disclose anxieties, enter and tolerate challenging psychedelic experiences, and feel adequately supported to metabolize these afterward (45) (**Figure 1**).

**Figure 1:**
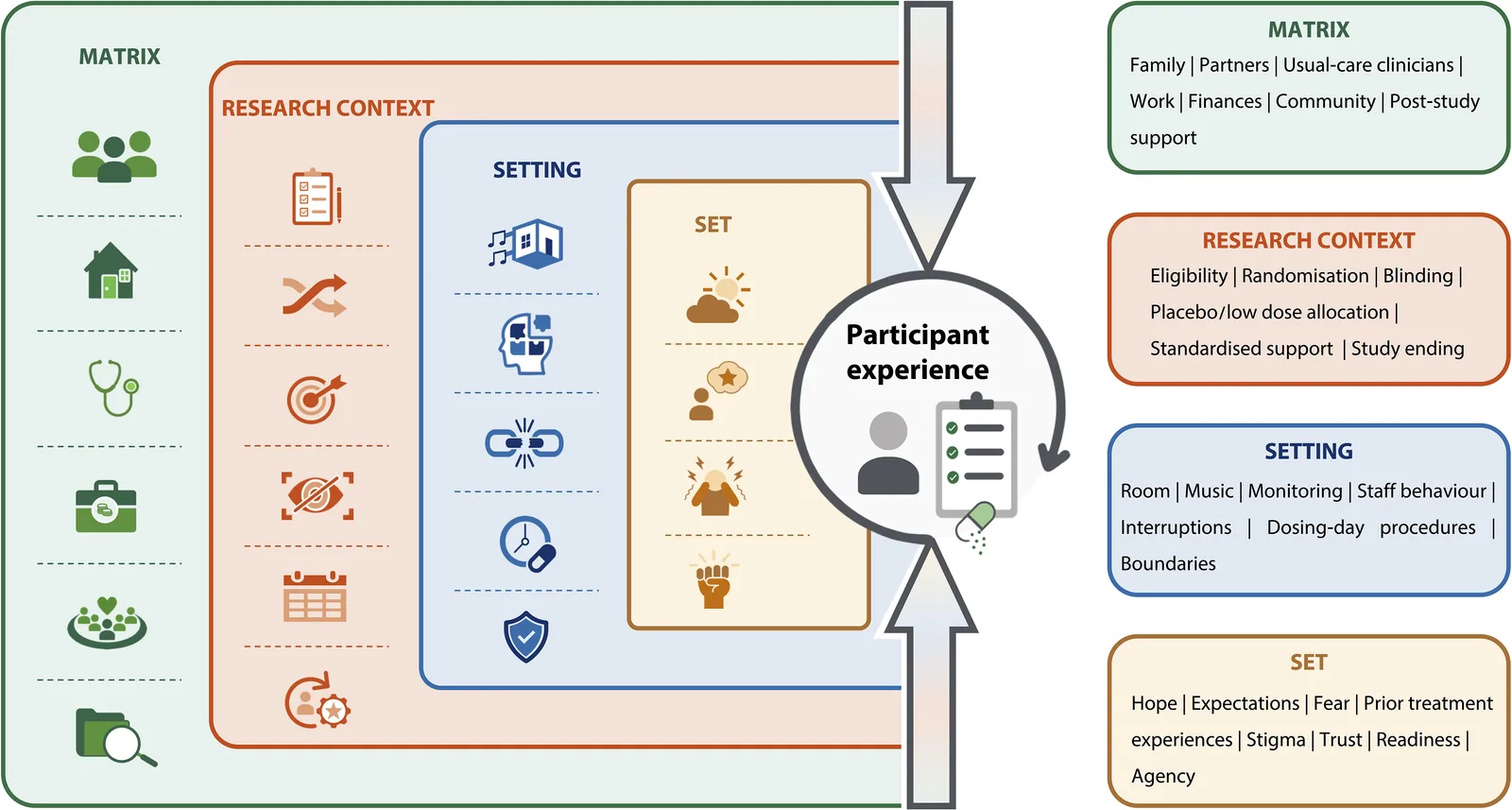
Contextual factors influencing participant experience in clinical trials.

For the purposes of regulatory approval, however, sponsors and regulators have recently sought to distinguish “drug effects” from non-drug effects, a distinction that is complex and has been the subject of a lively debate (14, 45–49). Some late-stage programs and accompanying commentaries have attempted to standardize or minimize accompanying psychotherapeutic components and to frame them as “psychological support” rather than psychotherapy (48, 50, 51). Others have argued that such reduction may exclude clinically important common factors and contextual mechanisms (45, 47, 49, 52–54). Some have proposed approaching psychedelic interventions as “complex interventions” with multiple interacting components and non-linear causal pathways that cannot be fully understood by RCTs alone (55, 56), and have recommended embedded qualitative research on participant perspectives and experiences as part of intervention development and implementation.

Relative to the number of quantitative psychedelic trials, few qualitative studies have explored participants’ viewpoints (19, 57–70). Across published accounts, participants often reported mistrust or disappointment with mental healthcare after unsuccessful treatments and not feeling taken seriously, yet linked rapport with facilitators to therapeutic outcomes and valued calm presence, empathic understanding and warmth. Before dosing, participants reported anxiety or nervousness and limited time to build trust and prepare, often across three preparation sessions. Expectations varied, and preparation was considered important for exploring and managing them. Several wished they had been told that strong emotions could arise, and some recommended that experienced patients share their experiences during preparation to reverse mistrust and normalize responses. A second major theme concerned learning to navigate and surrender to intense, profound or overwhelming experiences and the difficulty of letting go of control. Participants described limited agency to steer frightening or challenging experiences. Afterward, many struggled to find words to capture their experience or explain it to others; making sense of challenging experiences required time, with insights becoming apparent over the following days and weeks.

The main therapeutic processes mentioned by participants were insights, altered self-perception, feelings of connectedness, transcendental experiences, and having access to an expanded emotional spectrum. Connecting with other participants could create a sense of connection, and help participants feel validated and understood. Group-based integration and peer-support groups were beneficial after study participation (71–73). Participants also expressed a desire for multiple doses, which they felt might be needed for sustained improvements, potentially starting with a lower dose to allow familiarization with the effects and their responses. They generally compared psychedelic treatments favorably with previous treatments, describing them as more effective, more rapid, and more focused on core issues or inner processes as opposed to therapy or antidepressants. A recent scoping review of qualitative studies of psilocybin-assisted psychotherapy in controlled settings noted that most studies had relied on individual semi-structured interviews at a single timepoint, focused on relatively short-term perspectives (61). The authors recommended examining how participants integrate insights and changes over time, including after study endings, and whether they seek follow-up interventions.

In diverse areas of healthcare, people with lived experience (PWLE), experts by experience (EBE) and advocacy groups have contributed to raising awareness, knowledge sharing, education, peer and family support, and engagement with healthcare professionals and policy makers, particularly in response to perceived systemic knowledge gaps (74–77). By bringing together experiential and professional expertise, advocacy groups can function as ecosystems of shared learning, where interconnected roles can give rise to a multidimensional understanding of complexity (78) and allow members to function as “ambassadors of knowledge.” The Psychedelic Participant Advocacy Network (PsyPAN) is a not-for-profit organization that seeks to improve the wellbeing of psychedelic clinical trial participants by building a community of experts-by-experience to shape best practice across all levels of the global psychedelic sector, so that the participant voice informs trial and treatment development. Members are former trial participants who have reflected on their participation over time, discussed it with others, and contributed through public events, scientific meetings, media interviews and publications.

The present study brought together PsyPAN members for a series of focus groups with experts by experience on psychedelic clinical trials, addressing two aims:

1. To explore how former trial participants understood and appraised their experiences across recruitment and screening, preparation and dosing, post-dose (integration), and the post-study period.
2. To generate participant-informed recommendations for improving the design, conduct and follow-up of psychedelic clinical trials and potential future treatments.

## Methods

### Study design

This study was part of a patient and public involvement (PPI) initiative by Beckley Psytech Ltd. (BPL), a clinical-stage biotechnology company developing psychedelic treatments, to better understand participant perspectives on psychedelic clinical trials and inform future study design, participant support and post-trial care. BPL and PsyPAN formed a Patient Council comprising four patient experts with experience of psychedelic clinical trials and three BPL team members with expertise in patient support, psychiatry, psychotherapy, and trial development.

Three remote focus groups were convened to gather insights into participants’ clinical-trial experiences, both personal and informed by the collective experiences of the 70+ PsyPAN members. They focused on: (1) recruitment and screening, (2) preparation and dosing, and (3) post-dose integration and the post-study period. Discussions were recorded, transcribed and summarized, followed by an in-person workshop to validate the identified insights and discuss potential support options.

The study design, including participant selection, iterative focus-group format, topic-guide development and summary reports, was informed by a qualitative descriptive (QD) approach combined with reflexive thematic analysis (RTA). QD is suited to under-researched, practice-oriented questions aiming to produce a rich and accessible account in participants’ own terms while remaining close to the data and avoiding theoretical abstraction (79). Iterative focus groups can stimulate exchange, comparison and co-construction of meaning among people with similar lived experiences, helping to identify priorities and ensure research is relevant to patients’ needs (80–82). RTA, as developed by Braun and Clarke, was used because it provides a flexible but rigorous approach to identifying and interpreting patterned meaning while explicitly recognizing the researcher’s active interpretive role (83–87).

The study received ethical approval from King’s College London (KCL Ethics Ref: LRU/DP-24/25-42660). All participants provided written informed consent. Participants were remunerated for their time at an hourly rate.

### Participants and sampling

Purposive sampling was used. Eligibility criteria were: (a) previous participation in at least one psychedelic clinical study; (b) willingness and ability to discuss these experiences in a focus group; and (c) involvement in PsyPAN or related participant advocacy. We aimed for diversity in indication, psychedelic substance, gender and overall study outcome. Participants were actively engaged in articulating, discussing and refining participant perspectives in public and peer settings, and could reflect on their own experiences and recurring concerns within a wider participant community.

Four participants were selected and took part in all three focus groups. Two identified as male and two as female. Three had participated in treatment-resistant depression studies involving psilocybin or N,N-dimethyltryptamine (DMT), and one in a psilocybin study for an eating disorder. Their accounts included substantial benefit, mixed or temporary benefit, and disappointment, allowing exploration across a range of perceived outcomes. None had taken part in a BPL-sponsored study.

### Development of the discussion guide

In line with QD methodology, prior literature was used as a sensitizing resource to guide inquiry. The discussion guide (**Supplementary Material File 1**) was developed following a review of the literature on participant perspectives in psychedelic studies, broader literature on set and setting, therapeutic alliance, trial participation, and post-trial care.

The guide was organized chronologically around three phases of trial participation. Prompts explored participants’ expectations, interactions with study staff, the perceived impact of contextual and relational factors, areas of unmet need, and recommendations for future studies. The guide was used flexibly so that participants could introduce issues not anticipated by the researchers and could respond directly to one another’s experiences.

### Data collection

Focus groups lasted approximately 60 min–90 min and involved the same four participants. The iterative format allowed participants to revisit topics, develop consensus and disagreement over time, and progressively develop more considered expert-by-experience recommendations, reflecting the collectivized nature of PsyPAN, where participant knowledge had already developed through dialogue.

The focus groups were moderated by three BPL team members (SL, CL, and MS), using established good practice for focus groups in mental-health research: a semi-structured guide, clear boundaries and expectations, attention to emotional safety, encouragement of quieter voices, and support for shared and divergent perspectives (82, 88–90).

A preliminary summary of the emerging findings was shared after the third focus group. A one-day in-person workshop with the same participants then provided an opportunity to review, elaborate and challenge the preliminary analysis and work collaboratively on possible solutions and recommendations. The workshop generated further data and refined the analytic account. Draft recommendations were subsequently shared with PsyPAN, and participants sought informal feedback from the wider network, allowing broader expert-by-experience input into their framing and wording.

All focus groups and the workshop were audio-recorded and transcribed verbatim using an AI transcription service (Otter.ai). MS checked and corrected all transcripts against the original audio recordings to ensure accuracy. Transcripts were de-identified and imported into NVivo 14 to support data management and coding.

### Data analysis

The analysis was informed by Braun and Clarke’s six recursive phases of familiarization, coding, initial theme development, theme review, theme definition, and writing-up (83–87).

MS read and re-read all transcripts and field notes, listened back to the audio recordings, and wrote preliminary notes and reflexive memos. Coding in NVivo 14 began with broad organizational categories reflecting the discussion guide and temporal phases of participation. Within these, codes were developed inductively from participants’ accounts, compared across participants and focus groups, and clustered into topics. Through iterative rounds, a detailed report was developed (**Supplementary Material File 2**: Focus Group Discussion Topic Summaries Report), organized around successive study phases and retaining detailed quotations to preserve participants’ words and meaning.

The coding was reviewed by AOE, an experienced qualitative researcher, and coding was discussed to challenge assumptions, refine topic boundaries, explore alternative interpretations, and ensure that the developing analysis remained grounded in participants’ accounts. Both the detailed topic report and the themes presented, accompanied by their supporting quotations, were also discussed with the wider author group, including the experts by experience.

The report formed the basis for the development of five themes, defined as patterns of shared meaning. Consistent with RTA, these were actively developed through iterative engagement with the transcripts, the workshop material, and the authors’ evolving interpretations.

### Reflexivity and researcher positioning

Given the inherently interpretive nature of qualitative research, the professional backgrounds and theoretical orientations of the research team influenced both data generation and analysis. The first author (MS) is a psychiatrist and trainee Jungian analyst who has previously worked as a study doctor in academic psychedelic clinical trials and is currently overseeing medical and psychological support activities in psychedelic drug development. CL and SL also had experience in participant engagement and psychedelic study development within BPL.

The data were intersubjective and co-constructed through dialogue between former trial participants in an advocacy network and researchers with experience of designing, delivering and reflecting on psychedelic studies in commercial and academic settings. During the discussions, differences between the perspectives of experts by experience and researchers were actively explored and informed the analysis and results, particularly Theme 1.

The expert-by-experience co-authors’ contribution was grounded in more than individual trial participation. Their knowledge developed through taking part in psychedelic clinical studies, reflecting on those experiences over time, and comparing them with other former participants’ accounts. Recurring concerns emerged around recruitment, preparation, dosing support, integration, study endings, and community. Over subsequent years, these insights were further shaped through PsyPAN discussions, integration groups, advisory roles, trial-steering groups, consultation with researchers and sponsors, public engagement, media work, conferences, and dialogue with clinicians, academics, regulators, and other participants. This process transformed personal experience into a more collective form of experiential expertise.

## Results

**Table 1** summarizes the topics covered during the focus groups. A detailed report of all discussion topics, including extensive participant quotations, is provided in **Supplementary Material File 2**: Focus Group Discussions Report.

**Table 1:** Overview of topics covered during the focus groups.

Study phase	Topics covered
Recruitment and screening	• Expectations about study participation • Challenges during recruitment • Communication during recruitment and screening
Preparation	• Therapeutic approach and establishing trust • Learning to navigate the psychedelic experience • Optimizing the preparation phase
Dosing day	• Trust, surrendering, and moving through difficulty • Facilitators’ presence, support, and use of touch • Creating a therapeutic setting
Post-dose, integration	• Processing psychological changes • Integration support • Working with disappointment • Preparing for study ending • Tools for integration
Post-study period	• Post-study support

Five themes spanning the study trajectory are presented below, followed by participant-informed recommendations for future psychedelic trials and, where applicable, treatment delivery (**Figure 2**). **Table 2** illustrates each theme with a quotation.

**Table 2:** Illustrative quotations for the five themes.

Themes	Illustrative participant quotations
Theme 1: Participants are seeking a treatment, but encounter a clinical trial	*I have had contact with mental health services for 18 years, I’ve been in and out all kinds of different psychiatric institutions, I’ve been in inpatient units, I’ve been on multiple forms of medication. I’ve done CBT, dialectical behavioural therapy, MBT … I’ve done so many different things, which are all NICE guidelines approved, traditional forms of mental health treatment. And over the years, I have found all those experiences increasingly frustrating, they were scratching the surface, they were constantly putting a sticking plaster on the most enormous deep wound, and it was just not doing the job. So the idea that I will be trying something so radically different, is very appealing. (P3)*
Theme 2: Extending “set and setting” to the clinical-trial context	*Someone was mentioning the setting and how important that is to the experience on the dosing days, I think it’s crucial that people understand that the creation of that setting begins with the first contact with the potential participants. They may or may not end up on trial, but if they do, this is the beginning of your relationship, your trust building. It starts even when you’re just going through some boring paperwork. (P3)*
Theme 3: Facilitators as mediators	*I was scared to roll over, I felt frozen. I felt like I couldn’t turn around, because I was worried about being rude to the people on whichever side of the bed. It was really a symbol of how I navigate life. Worry about upsetting people, worrying about saying the wrong thing, all of this constant anxiety … My general trajectory with all of my psilocybin experiences has been resistance, terror, fear … Resistance really is what I now see my depression as, it’s resistance to thoughts, feelings, emotions, and the more I push, the tighter I am within that grip, the more brittle and restrictive it is. (P2)*
Theme 4: Dosing as the beginning of an ongoing process	*I felt some lasting impact from the experience for quite a few months afterwards. It felt like the psilocybin was showing me a whole new journey of healing that I could go on. And although it had been incredibly profound and in some ways transformative in the short term, I had the sense that it was the beginning of the end of a journey, a useful window into how I might be able to work on the difficulties I have. But then the frustration and disappointment rushed in because I thought: “well, how do I do that?” It was just such an interesting, alternative approach to self-healing, with potential for continuing to be useful in that respect. (P3)*
Theme 5: Unmet support needs after study ending and the value of peer support	*I think that the field is moving towards redosing and understanding that this isn’t a “once and done” treatment. And what’s really interesting in the integration space is having peer support, meeting others who have been through the same clinical trial process, which is quite unique and really important. You usually do not get this in an ordinary integration groups. I think there’s something really important about the peer connection and finding the others. (P1)*

**Figure 2:**
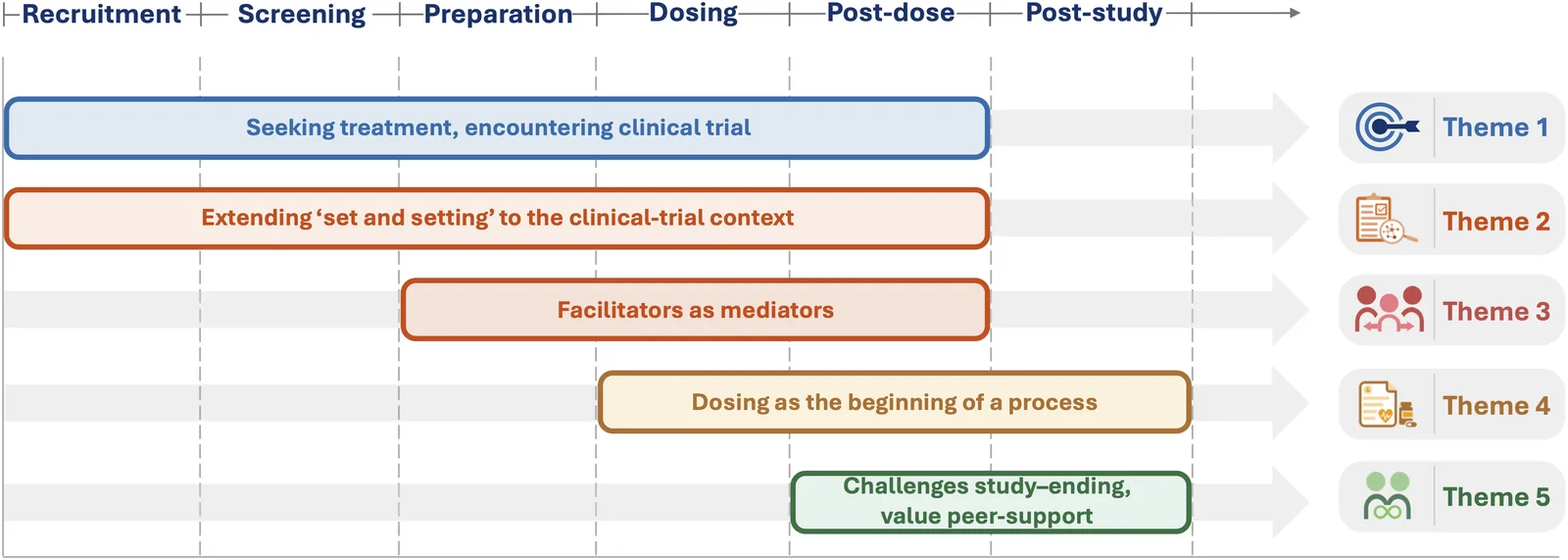
Themes in relation to study phases.

### Theme 1: participants are seeking a treatment, but encounter a clinical trial

This theme highlights the difference between participants’ reasons for taking part and what they hoped to find, and the aims for which clinical trials were designed (**Figure 3**). Participants hoped to access an effective treatment; pilot studies aimed to inform intervention development, whereas RCTs aimed to assess treatment safety and efficacy in a patient population (91).

**Figure 3:**
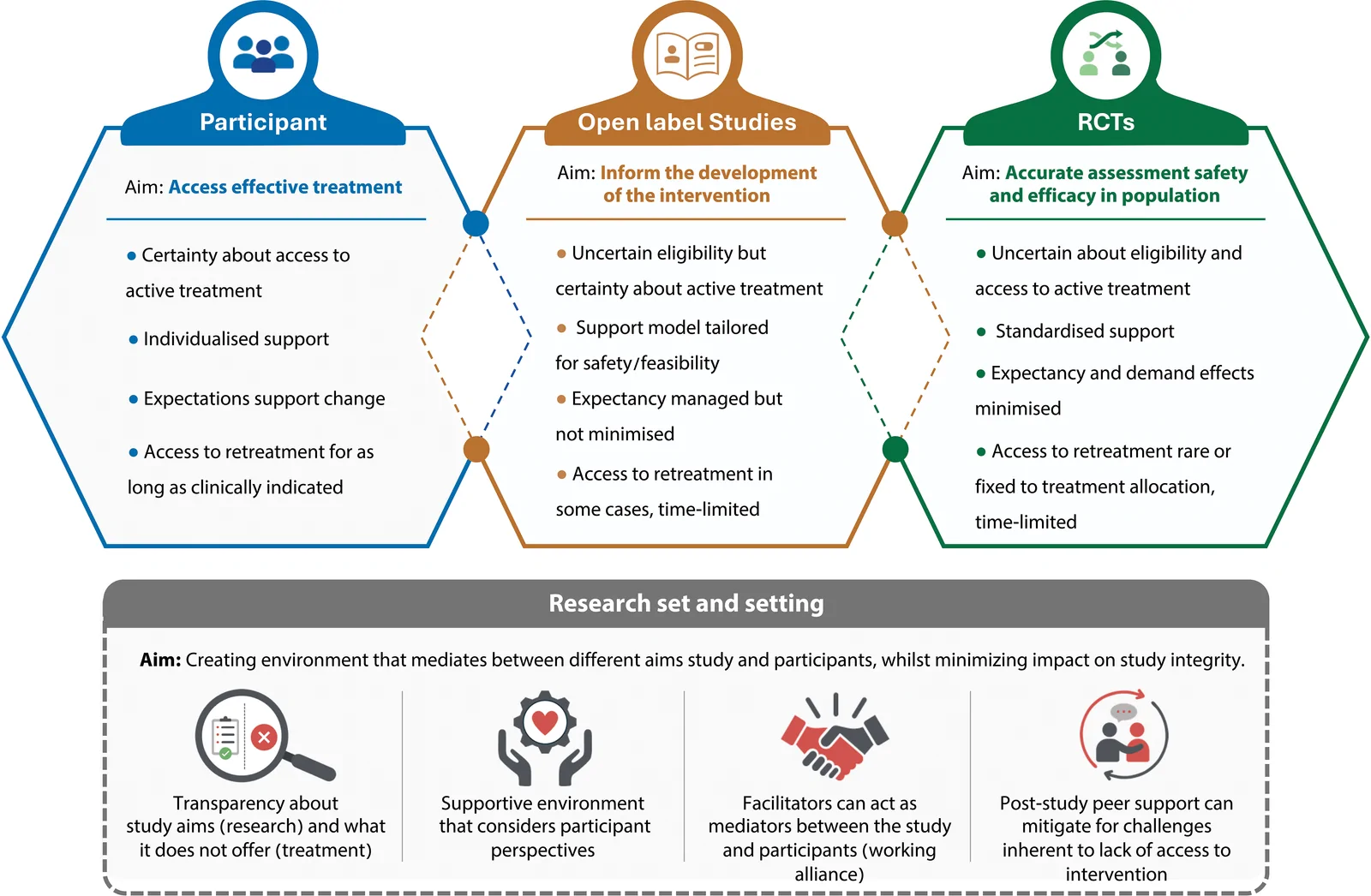
Psychedelic trial participation is shaped by the interplay between participants’ treatment expectations, study objectives and the research setting.

This lack of alignment was most evident during the early stages of the studies but remained apparent throughout the study journey and subsequent themes.

#### Hope to find a better treatment but mixed expectations

All participants had previous experiences with failed treatments, and their interest in taking part in a psychedelic study was often motivated by hope to access an effective treatment. A sense of having exhausted available treatment options, and the perception that psychedelic interventions offered a novel and different therapeutic approach, contributed to their appeal.

Expectations about study participation were influenced by personal histories, including previous challenging psychedelic experiences, cultural beliefs and the views of support networks and caregivers. Although partners were supportive, some participants experienced judgment or skepticism from friends or family and were discreet about participation. Medical professionals’ views varied; some GPs or psychiatrists advised against participation because antidepressants had to be tapered, or because of ethical concerns or perceived lack of scientific credibility.

“I wonder how much stigma is still attached? And how, even though these trials are completely legal, GPs would have ethical concerns around referring people? I’ve heard such varied feedback from people that have taken part in these trials, and sometimes you’re lucky, and other times people are just like: “you’re playing with fire”.” (P2)

Despite the growing evidence base and positive media reporting about psychedelic research, participants still reported feeling nervous or anxious going into the studies.

#### Challenges during recruitment and screening

All participants found screening stressful because of prolonged uncertainty around eligibility and perceived screening teams as focused on excluding rather than including them. Some found themselves guessing what the team sought, balancing demonstration of symptom severity with perceived risk.

“I felt during some interviews that I was trying to make my case. But it’s a fine line. I was trying to second guess, “What are they looking for?” Someone who needs this, but is not so unwell that they would be taking a massive risk.” (P1)

Although taxing, extensive screening raised some participants’ hopes and expectations regarding the trials’ potential impact, as contact with the study team increased anticipation, emotional investment and relationship development.

“My desire and desperation to take part grew during recruitment because I sensed this would be very different to anything I’d experienced before. By the time I’d spent a couple of months going through screening, I was desperate to be accepted and would have been crushed if refused.” (P3)

After applying to take part in a study, participants sometimes did not hear from research teams for prolonged periods. This delay could negatively affect their symptoms and mental health.

“I think there’s scope for better communications. I applied and it was nine months before I heard anything … We’re reaching out in desperation, so just even an acknowledgement or setting expectations in terms of timescales would be really helpful.” (P1)

One participant had been deemed ineligible and recounted disappointment and despair, amplified by limited clarity about the reasons.

“It felt like a distant, administrative team rather than caring people who wanted to make sure you were okay … I knew why I’d failed screening, but not the detail or nuance, and needed that information to know not to apply for similar trials. I was very disappointed not to take part, but that was compounded by the sheer admin. It felt chaotic.” (P2)

Participants also reflected on the broader burden of participation, noting that time, travel and repeated appointments could narrow who was practically able to take part.

“The population of trial participants is still very narrow demographically, and that needs to change. It takes a lot of time to take part in a trial, with all the different appointments. If you’re on a low income or have family commitments, you’d probably be priced out…” (P2)

### Theme 2: Extending “set and setting” to the clinical-trial context

In response to the tension between what participants sought and what studies could offer, participants described how the research context itself shaped their experience. We use the term “research set and setting” to extend the conventional concept of set and setting beyond the dosing session to include research-specific conditions across the trial trajectory. These included uncertainty created by eligibility criteria and placebo control, requirements for standardization and neutrality, limited opportunities for retreatment, and time-limited relationships with study staff. The subthemes show how communication, expectation management, facilitator relationships, and practical preparation could make these conditions more supportive, while recognizing that some constraints are intrinsic to particular study designs.

#### First impressions during recruitment set the tone for study experience

Creating a supportive and trustworthy setting began with the first interactions during recruitment, setting the tone for the overall experience and subsequent trust. When research teams acknowledged that screening could be emotionally taxing, they demonstrated understanding and support for participants’ wellbeing. Flexibility in the timing of surveys contributed to a more manageable screening experience.

Sensitivity about communicating ineligibility was considered part of the research team’s duty of care. Participants preferred an in-person conversation or call to email, ideally with discussion of other available treatment options.

“I think you have a duty of care even from the very beginnings of that recruiting process, you’re working with people who have mental health issues … People are coming to you in a state of desperation of feeling like it’s a last resort … you have to be sensitive to the impact of disappointment if people aren’t accepted.” (P3)

#### Managing expectations: preparing for all possible experiences

Once deemed eligible, an essential aspect of preparation was education about potential dosing experiences. This included psychedelic effects and how to respond, variability in individual responses and the possibility of low-intensity or “non-experience,” especially in placebo-controlled studies. Rigid expectations about specific experiences should be moderated to avoid disappointment. Participants also recommended introducing the possibility that unexpected or challenging experiences could still be helpful.

“If you go into it thinking: “I’m going to be cured of my problem”, than it’s a really hard experience to have, if you don’t get that outcome. I think if you’re holding in mind that there may be something of value to be got from whatever experience you encounter, that can be a helpful reframe. Not at all to suggest that bad experiences don’t occur, they do, but it’s just trying to set the person up for having a method, before they even do the experience, for reframing it if it’s a challenging one. I think providing people with that tool as early as possible is absolutely essential.” (P3)

Some of the focus group participants who had received multiple ascending dose levels warned against the assumption that lower doses equated with lower intensity.

“It’s really important to not equate higher doses necessarily with more intense experiences. I had three doses. The first session was the lowest dose but I had an extremely intense experience on what was definitely a low dose. There was very minimal visual change … but I actually had one of the most emotionally intense experiences in my entire life and released a huge amount of trauma during that time … I think that says a lot about the trust I felt in my sitters.” (P3)

Based on their experiences in studies with different psychedelic substances, another participant cautioned against equating a more intense experience with greater therapeutic value.

“Just because the experience is more intense it doesn’t necessarily mean it’s a better or a more healing experience … During my DMT experience, I didn’t know which way was up for most of that, it was surreal. My psilocybin experiences are more autobiographical … being able to revisit those experiences with a sense of which way is up. One should be careful not to fall into thinking that more intense is always better.” (P1)

#### Facilitator interactions modeled the approach to dosing

Participants found that the open, relaxed and welcoming atmosphere created by facilitators helped them open up. Compared with psychotherapy, facilitators were felt to position themselves alongside participants rather than as experts.

“The most important insight throughout my process was that I’m the source of my own healing. I don’t have to rely on others to give me the answers. That was the opposite of what I’d experienced in mainstream medical care, where I would constantly be thinking of therapists, doctors, mental health nurses, as the holders of some kind of magical answer … the authority figures that I was constantly deferring to. And the experience on the trial was so remarkably different to that.” (P3)

As part of the “research set,” this non-directive approach during preparation modeled an essential element of dosing. Encouraging participants to search for answers themselves helped build agency and confidence toward the psychedelic experience. Facilitators were likened to guides who knew the territory and could support participants to find their own way, rather than medical experts who “knew the way” to a cure.

Given complex personal histories, possible childhood adversity and extensive journeys through healthcare, some participants indicated that building enough trust to be vulnerable with facilitators required time.

“During my dosings I went to some pretty dark and difficult places. I only did that because I felt safe enough to, because of the relationship I’d built up with the guides. That relationship started six or seven months prior to actually having the doses. The therapeutic relationship doesn’t start the moment the person has taken a substance and is lying down on the couch, it starts way beyond before that.” (P3)

“There is that time aspect of “I’ve only known you for two weeks, and here I am laying myself bare and being completely vulnerable in your presence”. For me, that’s an issue anyway, because of what I went through as a child with the abuse, and all of that side of things.” (P2)

Participants agreed that exploring their personal histories helped prepare for dosing. By building a joint map of their inner psychological landscape, including experiences, memories or anxieties that could surface, participants could consider possible responses and how facilitators might support them.

“With the initial conversations, they’re trying to build that important bond of trust between you and the team, within just two or three sessions. And it’s not framed as a therapeutic session. It’s framed more as getting to know you. But you go on a really deep dive, you dig up a hell of a lot of stuff, because that’s how you give them an idea of how both you and they can best navigate what might come up during the sessions.” (P2)

#### Tools for preparation

Discussions also covered several practical tools that research teams had offered participants during preparation to better navigate the dosing experience. Clarity about the facilitator’s role during dosing, boundaries around touch or movement, and being introduced to the dosing room prior to dosing day, including all elements during a “dress rehearsal,” reduced anticipatory anxiety. By incorporating guided meditations and breathing exercises into preparation sessions, participants were introduced to tools to self-regulate during the dosing sessions, and body scans were particularly helpful to prepare for more “visceral” psychedelic experiences. **Table 3** provides further detail.

**Table 3:** Tools for preparation.

Explaining the role of the facilitator	
Including how they would interact and what facilitators would be doing during the dosing session	*I got told a lot about what they would be doing to me and how they would touch me. They used a cabin crew analogy, that they were there to support me with whatever I needed, “We’re helping you on your journey”. But I didn’t know what they would be doing while I was journeying. That’s the only missing piece. I found my facilitator at one point using a phone which felt quite unnerving. But reading, writing, taking notes felt okay … just mentioning that. (P1)*
Clarifying session boundaries and use of touch	
Explicitly discussing session boundaries, the use of touch, movement and expression during the session, helped participants to worry less about how they would be perceived so they could surrender more fully to the experience	*To be given that permission is vital for me. During my first dosing, I was frozen on the bed, didn’t feel I could roll over, I was worried about wetting myself, embarrassing myself somehow. Thankfully there was a second dose. “Whatever comes up, just do what you have to do”. And I moved so much on that bed, I didn’t get up and dance or anything, but foetal I was, not worried about how I might be judged or perceived by people in the room. And the increasing trust helps with that. They really helped me (by saying) “wet yourself, it’s fine”, and I didn’t, but it was like getting rid of another bit of resistance for me. “I can’t let go fully, because otherwise, what might happen?” (P2)*
Learn tools to self-regulate during the dosing sessions	
Incorporating guided visualizations and breathing exercises into preparation sessions	*If we had a preparation conversation, they would often start it or end it with this very simple breathing technique, guided breathwork. And there was a kind of mantra, which was something along the lines of “I am here now”, as a grounding technique. It introduced me to a technique to help me get through challenging moments during the doses. There was a particular moment during one of the doses where I became very stressed out and anxious. And one of them very quietly reminded me of the technique to bring myself back to a place of safety. So I thought that was fascinating that they were beginning to just provide me with some tools that I could later use by planting seeds. (P3)*
Body scans were helpful to prepare for more “visceral” psychedelic experiences	*Doing a body scan and releasing tensions in the body, especially for DMT which is a very visceral experience, was a really supportive tool. It’s important to put these things in, these little seeds, these tools that you can use to support yourself. Being given them as part of the preparation helps. (P1)*
Offering metaphors to mentalise dosing experience	
Metaphors provided by guides helped participants to better understand and navigate the experience, modeling the importance of acceptance and surrender.	*One of my guides compared the experience to a roller coaster and was saying that people don’t get on a roller coaster and then start panicking halfway up and ask for the roller coaster to be shut down. You’ve just got a flow, you just go with the experience. And you’ll probably have a negative experience if you try to stop things. I thought that was a really good analogy and got me used to the idea that I just have to go with what comes up. (P4)*
Dress rehearsal for dosing day	
Being introduced to the dosing room prior to dosing day, including familiarization with components such as vital signs or a weighted blanket that could impact the dosing experience’s quality	*You’re being put in a hypersensitive, extremely vulnerable state and you need to trust that you can be that vulnerable. But every detail is amplified. So getting the room right, making sure that the person is introduced to the elements first as well. I wasn’t introduced to the weighted blanket that they introduced during my session, and I hated it. (P2)*

### Theme 3: facilitators as mediators

Participants described facilitators as key mediators of the dosing experience, with trust established during preparation enabling them to engage with and move through challenging psychological states. Facilitators’ calm and non-directive presence, timely reassurance, and encouragement to remain with or return to the experience were experienced as central to this process. For some, appropriately bounded physical touch further enhanced feelings of safety and supported meaningful engagement with difficult material.

Building on the previous theme, facilitators, as well as members of the wider study team, can be regarded as part of the interpersonal dimension of the “research set and setting.” They can function as adaptive mediators between participants’ individual psychological needs and the constraints of a standardized study protocol. This was particularly important during dosing, when the variability and intensity of subjective experience, alongside differences in personal histories and coping styles, necessitated support that was sufficiently attuned to participants’ moment-to-moment needs.

#### Trust, surrender, and moving through difficulty

“You have to form a deep bond for the treatments to work properly. So you can let go fully and surrender to it. And allow yourself to be that vulnerable.” (P2)

Participants linked trust developed during preparation to feeling safer during dosing, relinquishing fear of losing control, reducing tension, anxiety and discomfort, and engaging more deeply with the experience.

“I think it was a reflection that the preparation was done really well, I had developed that trust. And even on the first dosing, it became really intense. I think I had let myself go. I, I felt that trust.” (P3)

For some, difficulty letting go of control yielded valuable insights into depressive symptoms, illustrating the potential value of challenging experiences when sufficiently processed. The study team’s explicit encouragement to embrace whatever emotions or physical reactions arose modeled acceptance and helped this participant confront fear with less avoidance.

“Once I got through the dark phase … I got to a stage where I was like, “I can deal with anything”… “I can deal with sadness, I can deal with pain, I can deal with all of these different emotions, and approach them with openness”, which was the opposite of depression, where you’re constantly pushing back … And that carried on to afterwards, for several months, that openness. The seed for that was sown with the permission to do whatever I needed to do.” (P2)

This permission to explore freely resonated with another participant, for whom a simple phrase from their guide had a lasting impact:

“It stays with me to this day, and it’s only such a simple phrase. I was a bit concerned about: “am I doing the right thing, I’m not set up in the right position…?” And one of the guides said: “it’s your day” and that resonated so much with me. It’s time for you to explore, it’s all about you. Previous to that, I’ve been trying to keep so many people happy in situations, I’m not really thinking about myself. And when they said that: “it’s your day”, I thought: “yeah, it is. It is my day, I can relax”.” (P4)

Similarly, for the participant in an eating-disorder study, facilitators’ encouragement to move toward rather than avoid anxiety supported an impactful experience of moving through difficulty.

#### Facilitators’ presence and support during dosing

Facilitators needed to balance responsive guidance with confidence in the unfolding process and the participant’s capacity to navigate it. A calm and steady presence contributed to safety without feeling overly observed or judged.

“It’s like when you’re giving birth and you have a doula, the best doulas won’t be nervously looking at you all the time. What you need is somebody who’s just holding space and doing something gentle and nourishing. And allows the process to unfold because that’s what’s happening both with birth and with psychedelics, there’s a process unfolding. And if you need something, they’ll step in.” (P2)

Several participants reported that they were influenced by a concern about meeting the perceived demands of the scientific process and the expectations of the research team.

“I really felt like I let everyone down on one of the trials, I felt like there was so much expectation. Although I think I imposed that expectation. And I was like, “this has to go right”, and obviously, I’ve benefited the first time, so I was desperate for that lifting of the depression and anxiety again.” (P2)

Reassurance by the facilitators was deemed an essential component of managing these expectations, affirming to participants that there is no singular “right” way to navigate the experience, and modeling trust in the participants’ ability to engage with the process fully.

“The reassurance is key. I was terrified that I was going to somehow spoil it all. It felt like such an incredible opportunity. And I was paranoid that I was going to somehow not do enough to make the most of the opportunity. That’s a very familiar anxiety that I carried around with me my whole life. But I did need some reassurance from people that there isn’t a right or wrong way of doing it. You’re encouraged to engage with the whole process.” (P2)

As overt effects waned, participants emphasized remaining engaged because insights and understanding could still emerge. One participant found it helpful when a facilitator encouraged her to turn inward, even when she felt the effects had subsided and could have begun speaking or analyzing the experience.

“He looked at me and he said: “You should go back in. The medicine is still coursing through you.” I’m feeling perfectly in the room and agree, “Well, okay, maybe I do that” and I put the eye-mask back on. I could have continued speaking and started my usual process of controlling and analysing, boxing it up. But he was absolutely right, there were further deepenings and epiphanies to be had.” (P1)

#### Physical touch, consent, and boundaries

Participants distinguished three related aspects of touch: consent and boundaries, its embodied or symbolic function, and the meaning of touch being withheld or removed.

“I really struggle with touch and living in my head rather than in my body because of what my body’s been through. That’s been my refuge, I’ve been so disconnected. So for me it’s important to agree beforehand the parameters of what that touch may entail, to have a conversation with someone: “what are the boundaries?”” (P2)

For some, well-timed and sensitive touch enhanced safety and encouraged engagement with challenging internal experiences; within a trusting relationship, its embodied and symbolic meaning could also be important.

“I just think touch is essential because we process things cognitively, but we process things physically as well … A lot of my psychedelic experiences were related to themes of parenting, inner-child work, mothering … So, moments where the sitters were comforting me by having a hand on my back, on my shoulder, was part of this symbolic process of working through that.” (P3)

I also think the touch element of psychedelics is important. Coming back to the birth analogy, so much of this pain is preverbal and in some ways, it is about reparenting and holding. Touch is so powerful.” (P1)

By withdrawing touch, facilitators non-verbally conveyed separation, which the participant understood as an insight into self-reliance.

“It’s not just the moment of the touch, but when the touch is removed … I have this memory of this intense experience of being comforted in the depths of my pain … But at a certain point they removed it, where I understood symbolically, I had to retreat back into myself, and that part of my journey was understanding that ultimately, I had to carry it on my own. So there’s something very important about what the touch represents.” (P3)

Reflecting on their experiences as part of two clinical studies, another participant felt that physical touch had not been used sufficiently.

“I think touch is incredibly important and I wasn’t offered enough touch during the studies. I’m so pleased to hear that the anorexia study was different. While I can understand why there’s less touch potentially in clinical trials, I genuinely think it’s missing.” (P1)

The discussion covered the safety concerns about the use of physical touch as well, and participants agreed that video recording the sessions was an acceptable mitigation and was preferred over not using physical touch at all.

### Theme 4: dosing as the beginning of an ongoing process

This theme captures participants’ shared perspective that dosing was the beginning of a process of meaning-making and various changes. Processing the experience began as the acute subjective effects of the study drug were subsiding, requiring an initially protective and reflective post-dose environment, and continued beyond study completion. Changes arose non-linearly and at different paces, ranging from symptom improvement and improved wellbeing to greater emotional openness, psychological flexibility and shifts in perspective. Participants also described disappointment when expected benefits did not occur, when the experience was less subjectively intense than hoped, or when initial improvements faded over time. Several participants mentioned feeling like they let the study and staff down when they did not show benefits, resulting in feelings of shame. Supportive integration sessions with sensitive facilitators helped participants to process challenges through reframing and exploring alternative meanings. Requiring further processing and integration of the experience beyond the sessions with the facilitators was common, and helpful tools included journaling, movement, creative expression, nature connection, playlists, guided meditations, and other somatic or reflective practices. Retrospectively, several participants conceptualized study participation as the beginning of a broader process of change marked by increased clarity and self-agency.

#### Processing changes

“The single most important piece of advice that I wasn’t given, and it’s been one of my key takeaways, is that this is the start of a process, and certainly not the cure.” (P1)

Participants understood integration to begin as the drug effects subsided and continue beyond the final study visit. Several recalled an urge to share, capture or analyze their experiences immediately, fearing they might not retain them. They also emphasized protecting time and space for meaning-making to happen gradually and avoiding a prematurely fixed narrative. Facilitators helped participants balance these impulses.

“I felt a lot of anxiety about “what if I forget all this? What if it washes away?” And the sitters were just trying to reassure me “you don’t need to make sense of it right now, we will come back to this and have an integration discussion the following day”… They were quite good at reassuring me: “do whatever feels right, you can do some writing when you get home if you want to…” I was given some advice about things I may want to do.” (P3)

For the evening after dosing and the journey home, participants valued a protective environment that allowed processing and limited unhelpful influences. Access to outdoor space was valuable for one participant.

“All I wanted to do was connect with nature and just feeling. feeling the softness of the ground beneath my feet, feeling the breeze on the skin, my face. It was amazing. A dog running around … it’s such a beautiful experience to do that. Just looking at the clouds and just really being in that hyper present state still. In that whole moment of experiencing and fully feeling every emotion.” (P2)

Participants recommended considering when to return to work and social obligations, allowing additional time for gentler reintegration and choosing carefully whom to tell. Questions from family members and a therapist felt confusing and overwhelming for one participant.

“One of the best pieces of advice someone gave me was to not talk about it too quickly. And to feel I was getting permission not to answer the questions. Choose who you share your story with very carefully. That was incredibly useful advice.” (P1)

“Because there’s a lot of questions from all sorts of people, but where do you begin? “How do you think it’s helped you?” Because I was sort of midway through CBT at that time, which wasn’t going very well. And it was just endless questions from the CBT therapist, “what happened, how do you think it’s helped you in this?”” (P4)

In the course of the first days after their dosing experience, participants became aware of changes in their mental health symptoms.

“I remember the question being posed, “Are you depressed?” and I was worried about killing the fairy … I paused for quite a while… “No, I don’t feel depressed” and that was amazing to be able to say that.” (P2)

Other changes emerged over subsequent weeks and months. Participants shared a sense that the study began a process of change requiring ongoing engagement and support.

“The outcome isn’t just about the dose, it’s not about the trip, it’s about outcomes convening in a week, a month, two months, three months time … because I could easily think that without any aftercare, the trial is a failure. “That doesn’t work for me, I’m back to nothing working for me again”. But ultimately, it’s served me really, really well.” (P2)

“I felt some lasting impact from the experience for quite a few months afterwards. It felt like the psilocybin was showing me a whole new journey of healing that I could go on. And although it had been incredibly profound and in some ways transformative in the short term, I had the sense that it was the beginning of the end of a journey, a useful window into how I might be able to work on the difficulties I have. But then the frustration and disappointment rushed in because I thought: “well, how do I do that?” It was just such an interesting, alternative approach to self-healing, with potential for continuing to be useful in that respect.” (P3)

For some, rapid changes were difficult to adapt to, even when positive, illustrating the need for ongoing support.

“We were speaking to somebody who was really struggling with the rate of change and equated it to crash dieting. It was such a massive change that they weren’t able to integrate that degree of change. So resources and support addressing this are necessary because it is not a slow and steady process. I think for many of our participants, having support integrating the rate of change would be really helpful.” (P1)

#### Integration support, disappointment, and study ending

Participants experienced varying degrees of disappointment because subjective effects were limited, symptoms did not change, or initial improvements faded. Some experienced this as personal failure and felt they had let the research team down.

“I’d been going through the difficulty, the darkness and going in and through that to the other side … but with my depression and anxiety coming back as I came back into the room, I was just disappointed … I felt like I failed. I had this whole team of people piling that effort and energy into me to help me and it hadn’t. My mindset at that point was it hadn’t worked.” (P2)

Despite the initial sense of failure and disappointment, the research team offered a more hopeful perspective that the experience could still lead to valuable insights if the participant remained open to possibilities of change that differed from their expectations and habitual ways of trying to cope with their depression.

“This is where the session the day after was so important … it was a psychiatrist who really helped me to reframe it. He was like: “Last time you got a gift, you got an afterglow, and that was great … This time, this is where the hard work begins.” … It’s very much like: “I didn’t get what I wanted, but maybe I got what I needed”. And the session afterwards helped me to embark on a different therapeutic path … The team were vital in helping me with that.” (P2)

Due to the strong rapport and bond that some of the participants had developed with the facilitators and study team, the end of the trial period invoked a sense of grief and loss. Explicitly addressing this with the facilitators and framing it as another opportunity to learn to let go, to separate, helped with a sense of closure.

“I felt a lot of grief at my trial ending because I trusted my guides so much … they had accompanied me in the rawest expression of my emotion, they had witnessed aspects of myself that I just don’t think other people have ever seen. So the trial coming to an end was quite a painful thing for me. They were cognizant of that … We were talking about it, I think that’s the key thing … I needed help to give a sense of ritual to the ending and closure." (P3)

A common thread that emerged when participants reflected on what had helped them manage the study ending was that facilitators and study teams had emphasized participants’ internal resources to improve their mental health, empowering them as agents of change.

“A major learning for me from the trial was that “The answer is within you”. It’s not another medicine or another doctor or another therapeutic approach - the answer is within. And being given the tools to be able to work with that within yourself; whether it’s journaling prompts, playlists, or body based practices.” (P1)

By positioning themselves alongside rather than above participants, facilitators mitigated dependency. Practices such as journaling, music and body-based work helped participants access further experiences of inner guidance and resources.

“I was given a lot of support with integration, but even with that, the onus really is on you, taking responsibility within that. If you’ve been someone who’s had years where everything you were indoctrinated to believe was: “The solution to your problem is in a service or medication or therapy”. It’s a big shift to think, “oh, gosh, no, the answer is in myself” That’s massive.” (P3)

#### Tools for integration

Participants suggested tools that had helped integrate their experiences and could be shared with study participants. They also recommended involving and educating the participants’ support network more actively. Further detail is provided in **Table 4**.

**Table 4:** Tools for integration.

Finding what works	
Participants benefited from a variety of approaches and practices to explore changes in their mental health in the course of the integration process and advocated for an individualized approach, following the participant’s curiosity.	*There’s not necessarily a right or wrong version of integration. I found some writing useful, but I also danced, I used my photography a lot, went on walks … There’s all sorts of different things. And I think just encouraging and empowering the person to find out what feels intuitive and natural for them. The last thing you want is to turn it into a piece of work, you don’t want to feel like set homework to do, because then you just stopped being engaged with it in the right way. (P2)*
Journaling and drawing	
Journaling had been helpful for several participants, as a way of recording the experience and insights of the dosing day to be revisited later to reconnect with the experience.	*I wasn’t encouraged to keep a journal, but I did. And it was so valuable during both trials. Going back to that was similar to going back to the playlist to go back to the dosing days, and being able to go through that process again, and to be reminded of the insights and the connections, the epiphanies, that experience was so valuable. (P2)*
One participant had received a booklet containing prompts to guide reflective writing developed by the research team to guide the process of integration.	*I was given an integration booklet to help me as a resource that I could use if I wanted to, to help with the integration process. It has various kinds of questions that I could try answering to as a kind of journaling guide. And it inspired me to keep a journal, but I was supported in that process with the excellent integration resource that the team had produced. (P3)*
Somatic approaches	
Body-based and physical approaches had been helpful for some participants.	*I think bodywork, dance, physical movement. Which I’m uncomfortable with generally, but that’s a path that’s opened up to me since the second trial and has really served me well. (P2)*
Watching the recording of the dosing session	
Access to the audio or video recordings of dosing and integration sessions could be helpful to hold onto the experiences.	*I think if patients are going to be given permission to view the video, it should happen in a therapeutic setting, with a therapist to work through it together and shouldn’t be given ownership, not to take home and potentially post on Instagram. Plenty of people might want to do that and maybe regret it later, particularly when people are sharing things on social media hoping for a positive connection and it not going well. (P3)*
Listening to the dosing playlist	
Participants recommended providing access to the study playlist after the dosing, as a tool to revisit and process the dosing experience	*In terms of integration afterwards, on my own, I would listen to the playlist, a lot. Got back into music more. (P4)*
Tools to support families of participants	
Participants discussed the need to better prepare and involve participants’ personal support networks, to reduce the burden on participants of having to explain what they are going through, particularly towards children, who might be affected by the degree of change their parent goes through and benefit from guidance on how to best relate to this.	*What is really important is support for families, something to give to them to help them support you during your integration process. You may not have the words at that point, and they may not know what to do. Something tangible that they can check to support you, without you having to say what support you need, would be really helpful. And I think planning in some activities, so children feel part of it, especially if there’s a massive degree of change. It’s like, “Well, mom’s like a different person. What’s going on right here?” I certainly started eating differently post-trial, really taking better care of myself and my body and just helping them with that too. We should focus on this as a sort of journey for the whole family. (P1)*

### Theme 5: unmet support needs after study ending and the value of peer support

For some participants, the study’s ending elicited feelings of loss and grief, and all participants indicated that they would have benefited from structured support beyond the end of the studies. They struggled to access psychedelically informed therapists, and some experienced return to their pre-existing mental health support as insufficient or frustrating. Most participants also wished they had further access to the therapeutic approach used during the studies. When symptoms returned and no formal route back into the treatment model was available, some participants described desperation and considered self-medicating, while also recognizing that this did not recreate the supportive context of the study. Integration groups hosted by research teams were experienced as valuable, but participants especially valued connection with others who had gone through psychedelic clinical trials and felt that open psychedelic communities did not fully address the unique context of trial participation. They therefore advocated dedicated, independent, trial-specific peer-support aftercare spaces to continue support and processing beyond study ending.

#### Experiences after study ending

Participants wanted continued integration after study ending, preferably with a psychedelically informed therapist.

“I started seeing my therapist after the first trial, and having the experience of going into the DMT trial and then going back into that therapeutic support is a huge improvement for me in terms of my integration and the support I’ve had. So I think that will be really important, some way of discharging people into something.” (P1)

“So, the stipulation from our trial was that I had to be going back to therapy or going into therapy afterwards … it was good, but it was also odd, because I had this experience on the trial, and then started a very traditional, typical, same old thing I’ve had before, an eating disorder therapy at an eating disorder service … she was nice, she didn’t stigmatise my experience, but I would much prefer to have been working with someone who was more psychedelically informed.” (P3)

All participants felt they would have benefited from continued access to the study intervention or psychedelic treatment model, especially when some of their symptoms returned. This concerned not only the study drug but the psychological support and structured healthcare setting around it, and some described desperation when no equivalent route was available.

“You’ve had this bright light shone on you and you’re like: “Oh, finally, all the weights lifted”, and then you feel it coming back, you’re like: “Well, what can I do?” But you now know, something can help you. I’m not saying there’s any comfort in that desperation of nothing helping you, but it’s what you know, and now you’ve been given this hope.” (P2)

“As the months have gone on, I’ve been really keen to get onto another trial, but mainly because it’s not just about the doses, but everything that can go hand in hand with that in terms of integration and support … For me, the desire to do it again is a feeling like there’s more work to be done. And I don’t feel able or have the resources to do that on my own … So that’s my motivation to want to do it again.” (P3)

Participants therefore suggested that future studies consider multiple dosing sessions where compatible with study aims, noting that trust and their capacity to surrender had developed through repeated exposure.

Participants had considered self-medicating after study ending but recognized that, without supportive context or expert psychological support, this would not recreate the intervention; its legal status further complicated access.

“I thought: “well, the only way for me to get that experience back is to self-medicate”, but I still felt like I needed the therapeutic support, somebody to talk to about the experience.” (P4)

#### Integration groups and peer-support

Some participants attended monthly integration groups established by study teams and led by clinicians, finding them helpful for ongoing integration.

“And then afterwards, there was an integration group … and those sessions helped me until COVID hit and then I stopped going. I desperately needed even more sessions, because I was lost and disappointed and really gutted.” (P2)

Gaining access to a supportive community of other people who had used psychedelics was valuable, but one limitation of these open groups was that most attendees had not taken part in a clinical study.

“Most of the people in that space hadn’t been on a clinical trial, I was very much a minority … Finding community was really powerful. I met very loving and caring people who made themselves available and said: “contact me if you’re struggling”. So that was amazing.” (P3)

Because psychedelic experiences occurred within the distinct context of a clinical trial, participants considered a closed group for former study participants more appropriate.

“I still think there is a value in having spaces for clinical trial participants, as opposed to having it for everyone and having experienced psychonauts there. That can work for some people, for other people it’s a very different path that they’ve taken. And if it is a sector-wide responsibility, funded by various organisations, I think that would be for the best.” (P2)

Participants concluded that an independent peer-support aftercare program for people who had completed psychedelic studies would best meet the need for structured, ongoing integration support.

“I think that the field is moving towards redosing and understanding that this isn’t a “once and done” treatment. And what’s really interesting in the integration space is having peer support, meeting others who have been through the same clinical trial process, which is quite unique and really important. You usually do not get this in an ordinary integration groups. I think there’s something really important about the peer connection and finding the others.” (P1)

## Discussion

This study explored the experiences and perspectives of participants involved in psychedelic clinical trials, with the aim of generating participant-driven insights and recommendations that can inform the design of clinical trials with psychedelic substances, and potentially future treatments.

Consistent with motivations for trial participation reported in clinical studies (92) and previous qualitative studies of psychedelic trials (19), participants described seeking a more effective treatment after inadequate responses to existing mental healthcare. Protracted failed-treatment trajectories, often required for eligibility, are associated with hopelessness, despair (93) and frustration with treatments and healthcare professionals (18). Presenting psychedelic interventions as radically shifting treatment paradigms (94, 95), particularly through media reporting, risks inflating hopes and expectations about outcomes, and a sense of “last resort,” exacerbating disappointment if these are not met (96). Participants’ accounts demonstrated how personal histories, cultural narratives and support networks shaped expectations, reinforcing recommendations to manage these carefully (96–98). Despite the “Michael Pollan effect” (99), the legacy of over-reporting psychedelic risks (100) continued to influence expectations and skepticism among family, friends and mental-health professionals. Although prior use of psychedelics has been linked to positive expectancy bias (101), one participant’s previous challenging experiences showed that this is not inevitable. Framing psychedelic interventions within established biopsychosocial models may help mitigate “psychedelic exceptionalism” and manage expectations among participants, research teams and media (47).

Because clinical psychedelic interventions were available only through study participation, participants found screening challenging because of uncertainty about eligibility and timelines and lengthy, emotionally taxing assessments. Recruitment teams seemed focused on exclusion—”screening out rather than in”—and participants tried to balance demonstrating need without presenting a risk profile that could preclude participation. Similar perceptions occur in other trials (92, 102), but may be especially consequential in psychedelic research, where subjective effects, expectations, investigator enthusiasm, and cultural narratives can influence experience and reporting through demand characteristics, functional unblinding and placebo responses (96, 103–106).

The burden of research is a major reason patients decline or withdraw from trials (107). Our findings echo qualitative research in other therapeutic areas: overwhelm during screening, anxiety around treatment allocation, frustration with repeated measures, and loss or isolation at trial ending (92). Inconsistent communication and insufficient education were modifiable sources of burden; information could be poorly timed, excessive, inaccessible or complex, and participants felt insufficiently informed about risks and benefits, equipoise, and randomization (92).

Extending “set and setting” to the clinical-trial context helps account for the interaction between participants’ treatment-seeking and trial procedures that may not align with those hopes, including fixed eligibility criteria, lengthy assessments, equipoise, randomization, masking, standardized support, and finite study contact. Transparent communication about timelines, processes and eligibility, together with acknowledgment of screening’s emotional impact, helped minimize anxiety and build trust. Ineligibility should be communicated sensitively and include signposting to support, as disappointment and rejection may exacerbate mental-health difficulties. These measures are consistent with patient-centered trial design generally (108–110), but may be especially important in psychedelic studies where context can exert a greater influence on outcomes (15, 111–113).

Within this mitigating milieu, facilitators were experienced as important mediators between research requirements and participant needs. Starting during preparation, participants valued facilitators’ open, empathic attitude and non-directive approach, consistent with preferred characteristics in non-clinical settings (114). By modeling non-avoidance and acceptance, facilitators encouraged perspectives linked to therapeutic outcomes (112, 115). Through exploring personal histories, participants and facilitators developed a shared understanding of the participant’s intrapsychic space, potential challenges during dosing—likened to a map of the territory—and how to respond. Compared with previous therapeutic experiences, facilitators positioned themselves alongside participants as collaborative “guides” rather than “authority figures holding answers.” Education about the range of possible experiences, from low-intensity or absent effects to challenging experiences, tools to regulate affect such as breathing exercises or guided visualizations, familiarization with the dosing room, and clarity about boundaries around touch, expression and movement fostered agency, confidence and psychological safety. Agency, the capacity to act, think or respond differently, may be supported through information, skill-building and introspective self-direction (116). Establishing sufficient trust could nevertheless be difficult within limited preparation, particularly after adverse life events or previous interactions with healthcare professionals, consistent with earlier participant accounts (19). Recent frameworks to assess and optimize preparedness (117, 118), and a validated Psychedelic Preparedness Scale (119), include many of these factors.

During dosing, participants reported that trust in facilitators, themselves and the study process, developed during preparation, lessened anxiety, tension, discomfort and fear of losing control and allowed deeper engagement. This is consistent with the role of a sufficiently developed therapeutic alliance in “holding” and “containing” participants (37, 39). Facilitators balanced supportive presence with encouragement to focus internally, minimizing distractions and mitigating disruptions. Confronting challenging material in this supported context sometimes yielded insights, including recognition of emotional resistance in depression (120). Used sensitively and within pre-defined boundaries, physical touch enhanced safety and engagement with challenging or non-verbal content for some participants, including through embodied or symbolic processing. Concerns about consent and appropriate boundaries aligned with wider ethical discussions (121–123); participants advocated clear consent procedures and safeguards such as video recording. The physical setting also mattered because minor elements could acquire symbolic meaning. Participants stressed the influence of the wider research team as part of the interpersonal setting, and continuity of staff throughout the study, particularly when multiple doses were offered.

The integration process began as the acute effects subsided but continued beyond study completion, requiring an initially protective and reflective post-dose environment for gradual processing. Reported changes were consistent with published literature (124) and could be immediate or emerge over time, ranging from improved wellbeing and symptom reduction to emotional openness, psychological flexibility and shifts in perspective. Some participants experienced disappointment when expected benefits did not occur or initial improvements faded over time. Several also felt compelled to meet perceived expectations by contributing positive outcomes, exacerbating shame or a sense of failure when this did not occur and illustrating possible demand characteristics (103, 104).

Supportive integration sessions with sensitive facilitators, including reframing experiences and exploring alternative meanings, helped participants process such challenges. Even positive changes, especially when rapid, could feel overwhelming or destabilizing and benefit from support. Practices such as journaling, movement, creative expression, nature connection and mindfulness-based techniques supported ongoing integration, including where participants had previously found them unhelpful. Access to music or meditations used in the studies helped some reconnect with the dosing experience. Our findings are consistent with observations that “psychedelic integration” is a broad concept extending beyond the final study visit, includes varied practices (125), may benefit from further development (126–128), and does not always fully contain participants’ experiences (19, 23). Retrospectively, participants described study participation as the beginning of a process of change marked by increased clarity and self-agency, which continued to evolve through later experiences and relationships, akin to a developmental process or individuation (129).

For some participants, study ending elicited loss and grief after significant attachment bonds with the team. All felt they would have benefited from ongoing structured support, especially with a psychedelically informed therapist, but access was limited by cost and returning to pre-existing mental-health support could feel insufficient or frustrating. All sought ways to participate in similar studies, and limited options led some to consider self-medication while acknowledging the importance of therapeutic support. Participants recommended longer-term integration options and, where feasible, retreatment after relapse. They valued integration groups hosted by research teams and sharing experiences with others who had completed similar studies. They also called for dedicated, independent peer-integration spaces tailored to the distinctive challenges of psychedelic trial participation. These priorities accord with recent recommendations for post-trial care, including peer-support integration groups (71). Participants also emphasized involving their support network, including healthcare professionals, partners, family and other supportive people. This extends Betty Eisner’s “matrix”: the socio-ecological context surrounding participants before and after drug exposure, including family dynamics, social relationships, living circumstances and community support (25). With consent, involving this network across study phases and after study ending could help participants feel supported and better able to integrate experiences into their lives and relational environments.

Participant-centered recommendations for each phase are summarized in **Tables 5**, **6**. Many have also been highlighted elsewhere (19, 117), although regulatory and scientific requirements in pharmaceutical drug-development studies can constrain their implementation. Some suggestions for improving trials may therefore be more applicable to future approved treatments. Recommendations are therefore classified by likely applicability to: (1) randomized controlled trials (RCTs), including blinded and placebo-controlled drug-development studies; (2) open-label studies (OL), including pilot, feasibility, dose-finding or extension studies; and (3) future psychedelic treatment delivery (Tx), should treatments receive regulatory approval.

**Table 5:** General participant-centered recommendations.

Phase/domain	Participant-centred design consideration	Applicability
Research/treatment frame	From first contact, distinguish the research frame from clinical care. In RCTs, explicitly explain eligibility, randomization, placebo or low-dose allocation, fixed visit schedules, time-limited support, possible non-response, loss of durability or relapse, post-session difficulties and study ending.	RCT, OL
Expectation management	Elicit prior treatment histories, media exposure, previous psychedelic knowledge or use, hopes, fears, and assumptions. Use scientific equipoise to balance hope with uncertainty, counter therapeutic misconception and psychedelic hype, and prepare participants for benefit, minimal benefit, or no response.	RCT, OL, Tx
Research set and setting	Treat recruitment, screening, preparation, dosing, integration, and ending as part of the participant’s research set and setting. Maintain predictability, neutral interpersonal tone, procedural clarity and sensitivity to how study procedures shape expectancy and meaning-making.	RCT, OL
Therapeutic milieu	Train all participant-facing staff, not only facilitators, in sensitive communication, emotional containment, boundaries, confidentiality, neutrality and awareness of the emotional impact of assessments, procedures, and perceived demand characteristics.	RCT, OL, Tx
Staff continuity	Where feasible, maintain continuity of key participant-facing staff across preparation, dosing and integration. When changes are unavoidable, prepare participants transparently to reduce uncertainty and preserve trust.	RCT, OL, Tx
Participant agency	Frame participants as active collaborators within protocol-defined boundaries. Emphasize self-discovery, choice and internal resources, while avoiding signals that the team expects a particular experience, response or narrative of transformation.	RCT, OL, Tx
Support network and matrix	With participant consent, provide concise information for partners, family members, support persons, GPs, psychiatrists or therapists on expected variability, possible post-session difficulties, how to support the participant, and whom to contact if concerns arise.	RCT, OL, Tx
Medication tapering and continuity of care	Where medication changes are required, provide clear timelines, clinical monitoring, realistic discussion of withdrawal, relapse or symptom-worsening risks, and coordination with usual prescribers where possible.	RCT, OL, Tx
Accessibility and participant burden	Consider the cumulative burden of repeated visits, assessments, travel, medication changes, time off work, caring responsibilities and reimbursement. Reduce upfront costs where feasible and communicate reimbursement procedures clearly.	RCT, OL, Tx
Participant-experience data	Consider embedding qualitative, acceptability or participant-experience measures across recruitment, preparation, dosing, integration and study ending to capture contextual, interpersonal, psychological, behavioral, and post-session difficulties not fully reflected in symptom scales or standard adverse-event reporting.	RCT, OL
Peer support	Consider structured trial-specific peer support, preferably after primary endpoint assessment or study completion in RCTs. Open-label and future treatment settings may allow earlier or more integrated peer support, with safeguards for confidentiality, boundary management, and contagion effects.	OL, Tx
Additional dosing and post-trial access	Consider open-label extension, retreatment, maintenance or redosing designs where scientifically justified, ethically appropriate and compatible with study aims, blinding, endpoint interpretation, safety monitoring, and regulatory requirements.	RCT, OL

**Table 6:** Phase-specific participant-centered recommendations.

Phase/domain	Participant-centered design consideration	Applicability
Recruitment and screening	Acknowledge enquiries where feasible, provide realistic timelines and communicate delays clearly, recognizing that silence can be experienced by vulnerable participants as indifference, rejection or lack of care.	RCT, OL
Recruitment and screening	Explain eligibility criteria and safety rationales in accessible terms and within scientific equipoise, while avoiding any implication that participants should shape symptom or risk reporting to gain entry.	RCT, OL
Recruitment and screening	Communicate non-eligibility sensitively, ideally with an opportunity for brief discussion, explanation where possible, and signposting to alternative support so rejection does not reinforce hopelessness or last-resort beliefs.	RCT, OL
Recruitment and screening	Acknowledge the emotional burden of repeated assessments and risk screening; allow reasonable flexibility, pauses, questions and discussion of distress triggered by procedures.	RCT, OL
Preparation	Allow sufficient preparation for participants to understand the protocol, meet key staff, discuss anxieties, expectations and usual responses to disappointment, and develop a workable sense of trust before dosing. Additional preparation may be considered when readiness or risk concerns arise.	RCT, OL
Preparation	Explore expectations, fears, prior psychedelic experiences, treatment disappointment, media narratives and hopes for change. Address therapeutic misconception and avoid implying that any specific acute experience, dose intensity or response trajectory is necessary or superior.	RCT, OL, Tx
Preparation	Explain the facilitator or monitor role clearly, including verbal support, silence, note-taking, physical positioning, monitoring procedures, neutrality, boundary limits and how participants can request help.	RCT, OL, Tx
Preparation	Discuss the full range of possible experiences, including intense, subtle, confusing, challenging, low-intensity, placebo-like, disappointing, non-beneficial or short-lived responses. Maintain neutrality around dose, intensity and therapeutic value.	RCT, OL, Tx
Preparation	Introduce simple grounding, breathing, body scan, visualization or attentional practices so they are familiar if used during dosing or integration. Offer metaphors or mantras as optional supports, not scripts participants must adopt.	RCT, OL, Tx
Preparation	Consider dosing-room familiarization or a brief dress rehearsal, including eyemask, headphones, blankets, monitoring equipment, room layout, staff movement and procedures likely to occur on dosing day.	RCT, OL, Tx
Preparation	Discuss boundaries in advance, including movement, emotional expression, toileting, physical touch, safety touch, therapeutic touch, video or other safeguarding procedures, and how consent will be documented, monitored, and revisited.	RCT, OL, Tx
Preparation	Plan the immediate post-dose period, including transport home, the first evening, return to work or care duties, reduced overstimulation, and involvement of chosen support persons where the participant consents.	RCT, OL, Tx
Dosing	Include a pre-dose check-in to revisit the plan, assess current mental state and risk, answer final questions, reconnect with intention and remind participants how to request support.	RCT, OL, Tx
Dosing	Create a protected dosing environment where avoidable noise, interruptions, unnecessary staff movement and unclear procedures are minimized, recognizing that small contextual details may become amplified during altered states.	RCT, OL, Tx
Dosing	Where safety allows, time and deliver physiological monitoring in ways that reduce disruption and preserve containment, especially during peak subjective effects.	RCT, OL, Tx
Dosing	Facilitators may aim to provide calm, responsive, non-directive presence without conveying surveillance, pressure, disappointment, outcome expectation or a single correct way to experience the session.	RCT, OL, Tx
Dosing	Where compatible with the support model, facilitators may gently encourage re-engagement with the experience when participants are frightened, distracted or avoidant, while preserving autonomy, safety and protocol neutrality.	RCT, OL, Tx
Dosing	If touch is permitted, use it only within explicit, documented and revocable consent procedures, with attention to timing, meaning, removal of touch, safeguarding, video or other oversight where appropriate, and local policy.	RCT, OL, Tx
Dosing	Consider comfort, lighting, music, access to toilet facilities, food, mirrors, corridor transitions and participant associations with hospital or medical settings, because contextual cues may shape safety, threat, and meaning-making.	RCT, OL, Tx
Immediate post-dose	Provide a post-dose debrief before discharge while reassuring participants that meaning-making can remain gradual, incomplete, and subject to later revision.	RCT, OL, Tx
Immediate post-dose	Offer concrete guidance for the first evening, including reducing overstimulation, cautious use of phones or social media, gentle activities, journaling if helpful, and careful choice of whom to speak with.	RCT, OL, Tx
Integration	Frame integration as an ongoing post-session process that may extend beyond formal visits, while being transparent about what support the study or service can and cannot provide.	RCT, OL, Tx
Integration	Make space for disappointment, shame, perceived failure, nocebo-like worsening or nonresponse after low-intensity, difficult, placebo-like, non-beneficial or short-lived experiences without premature positive reframing.	RCT, OL
Integration	Monitor for difficulty integrating rapid, destabilizing or positive changes in mood, identity, relationships, eating, work, family roles or self-care, and provide additional support or referral where needed.	RCT, OL, Tx
Integration	Offer optional integration tools such as journaling prompts, playlists, creative expression, movement, nature connection, mindfulness, somatic practices, or guided meditations.	RCT, OL, Tx
Study ending	Anticipate ending as part of integration. Discuss closure, dependency, grief, continuing resources, relapse or loss of durability, and how the participant can carry forward tools or insights independently.	RCT, OL
Post-study/post-treatment	Where possible, provide handover information to usual-care providers, with participant consent, and signpost to psychedelically aware therapists, charities, crisis resources, integration resources, or community support.	RCT, OL
Post-study/post-treatment	Consider structured peer-support groups after study completion or after primary endpoint assessment in RCTs. Open-label and future treatment settings may allow earlier or more integrated peer support with safeguarding, confidentiality and contagion-effect governance.	RCT, OL, Tx

Abbreviations and applicability codes: RCT, randomized controlled trial, including blinded or placebo-controlled drug-development studies; OL, open-label study, including pilot, feasibility, dose-finding, or extension studies; Tx, future treatment delivery after regulatory approval.

### Limitations

This study has several limitations. The sample was small, self-selected and demographically narrow, consisting of four white participants from psychedelic clinical trials conducted in the United Kingdom. The findings cannot be assumed to represent the full range of psychedelic trial participant experiences, including participants from more diverse ethnic, cultural, socioeconomic and diagnostic backgrounds, those who declined participation, failed screening, withdrew, had strongly negative experiences, or did not become involved in advocacy. The retrospective focus-group format allowed comparison, elaboration and collective meaning-making, but may also have encouraged convergence, inhibited some disclosures, or made disagreement less visible. The participants’ involvement in PsyPAN shaped the data. Their accounts reflected personal participation, peer discussion, public engagement, advocacy and exposure to published literature. This limits generalizability, but increases the depth and reflexivity of the expert-by-experience perspective. Claims about facilitators, preparation, touch, movement, integration or study effects should be interpreted as participants’ recollections and meaning-making. The study did not observe facilitator behavior, study procedures or therapeutic processes directly, and cannot establish causal mechanisms. The recommendations should therefore be treated as participant-informed design considerations requiring further empirical testing, stakeholder deliberation and adaptation to specific trial contexts. The study was initiated as part of a patient and public involvement initiative by a company developing psychedelic treatments. Although none of the participants had taken part in a study sponsored by that company, this context may have influenced the questions asked, the focus-group dynamics and the interpretation of findings. The professional, clinical, commercial, and theoretical positions of the authors should be considered when interpreting the analysis.

## Conclusion

Experts-by-experience perspectives emphasize the importance of psychosocial, interpersonal and contextual factors shaping participants’ experiences throughout trials. Alongside “set” and “setting,” participants stressed the wider social context: support from family, friends and healthcare providers. Their accounts underscore the tension between standardized, placebo-controlled protocols required for drug development and participants’ wish for personalized approaches aimed at therapeutic benefit. Although not all recommendations can be met within rigorous RCT designs, they can inform post-approval psychedelic-assisted therapies and enhance acceptability, efficacy and safety in practice. Meanwhile, peer-support integration programs may address unmet needs for financially accessible support after psychedelic studies.

## Data Availability

The datasets presented in this article are not readily available because Dataset contains identifiable quotes by participants and is not publicly shared, but quotes are contained within the publication. Requests to access the datasets should be directed to mathieu.seynaeve@kcl.ac.uk.
