# Supplementary material for: Participant experiences and recommendations for psychedelic clinical trials and future treatment delivery: a qualitative study with experts by experience from the Psychedelic Participant Advocacy Network
Source: Front Psychiatry. 2026 Oct 14;17:1682810. doi: 10.3389/fpsyt.2026.1682810 (PMC13713624; doi:10.3389/fpsyt.2026.1682810)
Supplement: Supplementary file 1 [file SupplementaryFile1.docx]

SM File 1: Focus Group Discussion Topic Guide

Supplementary material for: Participant experiences and recommendations for psychedelic clinical trials and future treatment delivery

Contents

Topic guide structure	2

Standard prompt domains	2

Detailed focus group topic guide	3

Session 1: Recruitment and screening	3

Session 2: Preparation and dosing	4

Session 3: Integration and post-study phase	5

# Topic guide structure

The focus groups followed the main stages of trial participation. Each session used the same prompt domains, with follow-up questions adapted to the discussion.

| **Session** | **Subsections** | **Focus** |
| --- | --- | --- |
| **Session 1: Recruitment and screening** | Treatment of condition prior to trial; Awareness/referral to clinical trials; Screening and medical preparations; Education and consent | Experiences before the trial, first contact with clinical trials, screening, medical preparation, education and consent. |
| **Session 2: Preparation and dosing** | Before preparation; Preparation sessions; Before dosing; Dosing; Post-dose before integration session 1 | Experiences before preparation, preparation sessions, anticipatory period before dosing, dosing day, and the immediate post-dose period before the first integration session. |
| **Session 3: Integration and post-study phase** | Integration sessions; After the integration sessions; After study ending; Experiences of relapse and recovery; Open discussion / additional issues | Integration sessions, experiences after integration, study ending, relapse, recovery and longer-term support needs. |

# Standard prompt domains

The following prompt domains were applied flexibly across the subsections listed in the guide.

| **Prompt domain** | **Intended focus** |
| --- | --- |
| Initial expectation or understanding | What participants expected or understood before this part of the study. |
| Description of actual event | What happened, from the participant perspective. |
| Thoughts during | Thoughts, interpretations or meaning-making at the time. |
| Feelings during | Emotional responses during the experience. |
| Pain points during | Difficult, burdensome, confusing or distressing aspects. |
| Surprises during | Unexpected positive, negative or neutral aspects. |
| Patient needs | Needs, unmet needs, and forms of support that would have helped. |
| Sensitive comment/topic | Issues that felt difficult, private or sensitive to discuss. |
| General comments / questions | Additional reflections, questions or recommendations. |

# Detailed focus group topic guide

The tables below show the topic guide by focus-group session. Prompts were not used as a fixed script; they were used to support discussion and to make sure each topic area was covered.

## Session 1: Recruitment and screening

Experiences before the trial, first contact with clinical trials, screening, medical preparation, education and consent.

| **Subsection** | **Discussion prompts** |
| --- | --- |
| Treatment of condition prior to trial | • Initial expectation or understanding  • Description of actual event  • Thoughts during  • Feelings during  • Pain points during  • Surprises during  • Patient needs  • Sensitive comment/topic  • General comments / questions |
| Awareness/referral to clinical trials | • Initial expectation or understanding  • Description of actual event  • Thoughts during  • Feelings during  • Pain points during  • Surprises during  • Patient needs  • Sensitive comment/topic  • General comments / questions |
| Screening and medical preparations | • Initial expectation or understanding  • Description of actual event  • Thoughts during  • Feelings during  • Pain points during  • Surprises during  • Patient needs  • Sensitive comment/topic  • General comments / questions |
| Education and consent | • Initial expectation or understanding  • Description of actual event  • Thoughts during  • Feelings during  • Pain points during  • Surprises during  • Patient needs  • Sensitive comment/topic  • General comments / questions |

## Session 2: Preparation and dosing

Experiences before preparation, preparation sessions, anticipatory period before dosing, dosing day, and the immediate post-dose period before the first integration session.

| **Subsection** | **Discussion prompts** |
| --- | --- |
| Before preparation | • Initial expectation or understanding  • Description of actual event  • Thoughts during  • Feelings during  • Pain points during  • Surprises during  • Patient needs  • Sensitive comment/topic  • General comments / questions |
| Preparation sessions | • Initial expectation or understanding  • Description of actual event  • Thoughts during  • Feelings during  • Pain points during  • Surprises during  • Patient needs  • Sensitive comment/topic  • General comments / questions |
| Before dosing | • Initial expectation or understanding  • Description of actual event  • Thoughts during  • Feelings during  • Pain points during  • Surprises during  • Patient needs  • Sensitive comment/topic  • General comments / questions |
| Dosing | • Initial expectation or understanding  • Description of actual event  • Thoughts during  • Feelings during  • Pain points during  • Surprises during  • Patient needs  • Sensitive comment/topic  • General comments / questions |
| Post-dose before integration session 1 | • Initial expectation or understanding  • Description of actual event  • Thoughts during  • Feelings during  • Pain points during  • Surprises during  • Patient needs  • Sensitive comment/topic  • General comments / questions |

## Session 3: Integration and post-study phase

Integration sessions, experiences after integration, study ending, relapse, recovery and longer-term support needs.

| **Subsection** | **Discussion prompts** |
| --- | --- |
| Integration sessions | • Initial expectation or understanding  • Description of actual event  • Thoughts during  • Feelings during  • Pain points during  • Surprises during  • Patient needs  • Sensitive comment/topic  • General comments / questions |
| After the integration sessions | • Initial expectation or understanding  • Description of actual event  • Thoughts during  • Feelings during  • Pain points during  • Surprises during  • Patient needs  • Sensitive comment/topic  • General comments / questions |
| After study ending | • Initial expectation or understanding  • Description of actual event  • Thoughts during  • Feelings during  • Pain points during  • Surprises during  • Patient needs  • Sensitive comment/topic  • General comments / questions |
| Experiences of relapse and recovery | • Initial expectation or understanding  • Description of actual event  • Thoughts during  • Feelings during  • Pain points during  • Surprises during  • Patient needs  • Sensitive comment/topic  • General comments / questions |
| Open discussion / additional issues | • Initial expectation or understanding  • Description of actual event  • Thoughts during  • Feelings during  • Pain points during  • Surprises during  • Patient needs  • Sensitive comment/topic  • General comments / questions |
