# Supplementary material for: Participant experiences and recommendations for psychedelic clinical trials and future treatment delivery: a qualitative study with experts by experience from the Psychedelic Participant Advocacy Network
Source: Front Psychiatry. 2026 Oct 14;17:1682810. doi: 10.3389/fpsyt.2026.1682810 (PMC13713624; doi:10.3389/fpsyt.2026.1682810)
Supplement: Supplementary file 2 [file SupplementaryFile2.docx]

SM File 2: Focus Group Discussion Topic Summary Report

Supplementary material for: Participant experiences and recommendations for psychedelic clinical trials and future treatment delivery

Contents

Topic structure	4

Detailed topic summaries and quotations	5

1. Recruitment and screening	5

1.1. Managing expectations	5

1.1.1. Factors influencing expectations	5

1.1.1.1. Experiences with previous treatments or psychedelics	5

1.1.1.2. Opinions by healthcare professionals and support system	5

1.1.1.3. Cultural beliefs and media coverage on psychedelics	6

1.1.2. Challenges to expectation management during recruitment and screening	7

1.1.2.1. Uncertainty around eligibility	7

1.1.2.2. A prolonged screening process	7

1.1.3. Managing expectations through careful communication	7

1.1.3.1. Turning down the hype	7

1.1.3.2. Shifting the tone	8

1.1.3.3. Transparent timelines	8

1.2. Importance of adequate support during the screening	9

1.2.1. ‘Set and Setting’ starts with first contact	9

1.2.2. Support and sufficient time to complete scales and questionnaires	9

1.2.3. Communicating screen failures	9

1.2.4. Meeting disappointment	10

1.2.5. Signposting for non-eligible participants	11

1.3. Optimising Recruitment and Screening	11

1.3.1. Involving partners or support network in screening	11

1.3.2. Reimbursement for travel expenses	12

1.3.3. Information for GP and family	12

2. Preparation	13

2.1. Therapeutic alignment and building trust	13

2.1.1. Therapeutic alignment	13

2.1.1.1. Essential aspects of the therapeutic approach	13

2.1.2. Importance of building trust	14

2.1.2.1. Building trust takes time	15

2.1.2.2. Exploring personal history contributed to building trust	15

2.1.2.3. The entire team contributes to a trustworthy setting	16

2.1.2.4. Influence of the gender of the therapists	16

2.2. Learning to navigate the psychedelic experience	17

2.2.1. Balancing information with expectation management	17

2.2.2. Covering the range of possible experiences	18

2.2.3. Lower doses do not always mean less intense experiences	19

2.2.4. Research team setting the wrong expectations	19

2.2.5. Communicating that all experiences are welcome	19

2.2.6. Explaining the role of the guide	20

2.2.7. Feeling like letting down guides or research teams	21

2.2.8. Practical tools to learn to navigate the dosing experience	21

2.2.9. Breathing exercises and guided visualisation	22

2.2.10. Clarity around the use of touch and session boundaries	23

2.2.11. Familiarisation with the dosing room prior to dosing	24

2.3. Optimising the preparation phase	24

2.3.1. Challenges with antidepressant medication withdrawal	24

2.3.2. Creating the right setting/protected time	25

2.3.3. Scales and assessments are burdensome	26

2.3.4. Bringing meaningful objects into the dosing room	27

2.3.5. Leaflets for GP and support system	28

2.3.6. Involve partner or support system in preparation	29

2.3.7. Paying for travel expenses	29

3. Dosing day	30

3.1. Navigating the experience	30

3.1.1. Trust in guides and team helped to be able to let go of control during the dosing session	30

3.1.2. Difficulties with letting go of control	30

3.1.3. Moving through difficult emotions	31

3.1.4. Using humour to cope with darkness	32

3.1.5. The use of touch	32

3.1.6. Staying with the process and ‘going back in’	33

3.1.7. Freedom of expression during dosing day	34

3.1.8. Worrying about the guides or therapists	35

3.2. Influence of the setting	36

3.2.1. Sensitive and aware staff	36

3.2.2. Influence of the setting	38

3.2.3. The drama of the dosing ritual	40

3.2.4. Food during dosing	40

3.2.5. Minimising disruptions	41

3.3. Optimising dosing	43

3.3.1. Desire for multiple doses	43

3.3.2. First dose as preparation for a second dose	43

3.3.3. Support person pick-up post-dose	43

4. Integration	44

4.1. Processing changes and integration over time	44

4.2. Importance of support during integration	47

4.2.1. Not enough support	47

4.2.2. Reassurance and guidance	48

4.2.3. Working with disappointment	48

4.2.4. Reframing disappointment	48

4.2.5. Appropriate study ending	49

4.2.6. Locating agency in the participant	49

4.3. Optimising integration	50

4.3.1. Guided journaling	50

4.3.2. Drawing and visual expression	50

4.3.3. Listening to the playlist and guided recordings	51

4.3.4. Access to recordings or summaries	51

4.3.5. Returning to practices that previously felt unhelpful	52

4.3.6. Resources for sustained integration	52

4.3.7. Continuity of care after trial participation	52

4.3.8. Briefing the support network	53

5. After the study ends	54

5.1. Challenges after study ending	54

5.1.1. Anxiety about returning symptoms	54

5.1.2. Desire to access the study intervention	54

5.1.3. Finding a therapist for further integration	54

5.1.4. Seeking other psychedelic studies	55

5.1.5. Thoughts about self-medicating	55

5.2. Support after the study ending	56

5.2.1. Importance of peer support	56

5.2.2. Involving the whole family	57

5.3. Optimising the post-study period	57

5.3.1. Support networks and continued integration	57

# Topic structure

| **Study phase** | **Main topics** |
| --- | --- |
| Recruitment and screening | Managing expectations; Importance of adequate support during the screening; Optimising Recruitment and Screening |
| Preparation | Therapeutic alignment and building trust; Learning to navigate the psychedelic experience; Optimising the preparation phase |
| Dosing day | Navigating the experience; Influence of the setting; Optimising dosing |
| Integration | Processing changes and integration over time; Importance of support during integration; Optimising integration |
| After the study ends | Challenges after study ending; Support after study ending; Optimising the post-study period |

# Detailed topic summaries and quotations

# 1. Recruitment and screening

## 1.1. Managing expectations

Participants discussed the need to manage expectations during recruitment and screening, especially around eligibility, timelines, and the risks and benefits of participation.

### 1.1.1. Factors influencing expectations

#### 1.1.1.1. Experiences with previous treatments or psychedelics

All participants had previous experiences with failed treatments for their mental health difficulties, which influenced their expectations and hopes about the psychedelic studies.

Two participants of the focus group had been part of the first clinical trial with psilocybin for TRD (CH, 2016), and commented on the scarcity of information in the public domain at the time of their participation, and the difficulty in finding out about the study.

“I only found the trial due to months of research online looking at different ways that might help my depression, because the GP … basically said they couldn't do anything for me… it took a lot of digging, then, which it probably doesn't now… on a positive level, maybe people are more aware that these trials are available now, which is good. On a negative level. Yeah, there's, there's the hype to counter as well…”

“at the time when I went in… there wasn't really that much information to sort of give you any kind of expectation or, you know, is this gonna work for me, because you read these stories now that people have been cured… it's the panacea kind of thing. But there wasn't that when when we took part, it was, you know, still very, very early days.” (P4)

Their interest in participating in the study was primarily driven by a sense of having exhausted available treatment options and despite the lack of published clinical efficacy data for psychedelics, or positive media attention at the time of their participation influencing their expectations about outcomes, both participants mentioned perceiving the study as a ‘last resort’.

"it was sort of tried everything else. And, you know, this was sort of like a last resort almost.. I wasn't convinced that it was going to solve any big problem. I just thought it would be worth a go" (P4)

“I was in a very desperate place at that time. And I didn't want to pin everything on the trial, but I was very much in that state of mind, where if this doesn't work, what am I gonna do? You know, as like, I tried not to put all of my hope on it, but I did, I do think I still pinned quite a lot on it.” (P2)

“There's just one last thing I want to say about expectation, because I had previous psychedelic experience. And the last one I had was negative, that skewed my expectations in maybe a bit of a negative way. It created a lot of fear. But I was so desperate for something to work, that I was willing to sort of take that plunge, but I think I had more fear than somebody would have done if they were psychedelicly naive.” (P2)

#### 1.1.1.2. Opinions by healthcare professionals and support system

Although all participants felt supported by their partner to take part in the studies, some participants experienced judgement by friends or family members and preferred to be discreet about their participation prior to the study.

“I told as few people as I possibly could before the trial, because I didn't want their opinions to guide or sway or affect my experiences. And I think I told I told my wife, my sister and a close friend, my close friend had told his wife, and she said, He fed this back to me, which is exactly the sort of feedback I didn't want. She was like, "Oh, well, you know why, when the boat is already rocking, why is he rocking the boat even more" you know? And it was out of concern, but that was the sort of thing I really wanted to avoid hearing to be honest.” (P2)

The participants experiences with medical professional’s support and opinions about their participation on the studies varied significantly, with some participants experiencing their GP advising against stopping their antidepressant medication or potentially having ethical concerns about referring patients, perhaps influenced by stigma around psychedelics.

“When I've contacted my GP about it, he was saying, he didn't think it was a good idea. And I said, "Well, look, you know, I really want to take part in this trial". And he said I can't really agree to stop the antidepressants this suddenly, you need longer.” (P4)

I wonder how much stigma is still attached? And how, even though these trials are completely legal, GPs would have ethical concerns around referring people? I've heard such varied feedback from people that have taken part in these trials, and sometimes you're lucky, and other times people are just like: “you're playing with fire”. (P2)

Other participants had similar impressions of preconceptions amongst some clinicians, such as therapists and psychiatrists that psychedelic interventions might lack credibility.

“I think there's also a sort of, probably a perception amongst a lot of the clinical community that it's a bit woo and that it's not really, like, serious. It's not, you know, if you sort of said someone "I've just spent, you know, the last few months doing group therapy at the Tavistock they probably think, ‘oh, wow, that sounds sounds really valuable’. If you said, ‘I just did a psychedelic trial’, I think they probably still think it's a little bit hippyish, a bit odd, a bit off the wall and wouldn't necessarily equate it with the same sort of, yeah, therapeutic impact, I suppose. I certainly could kind of slightly detect that a little bit when I told people afterwards, but I've been doing, you know some people were like like, ‘allright, okey, interesting... ‘ you know?” (P3)

One of the participants felt that publication of trial results in reputable medical journals might help to shift perceptions amongst medical professionals, illustrated by one of her family members who’s a psychiatrist.

“For me, it's the moment it's published in the BMJ or the lancet. My sister, who used to be very anti psychedelics, and she's a psychiatrist. But the moment it was published in The Lancet or the BMJ even she started saying, ‘Oh, it looks interesting’” (P1)

#### 1.1.1.3. Cultural beliefs and media coverage on psychedelics

All participants agreed that a substantial increase in publicly available information on psychedelics as seen in the recent years, has inevitably affected how participants approach psychedelic studies, and deliberated over the optimal level of pre-trial information, indicating the difficulty accessing reliable information about psychedelics and the potential influence on expectations.

"Is it better to go in knowing the bare minimum? Or is it better to go into these trials... with a lot of knowledge?" (P4)

Participants commented on their conscious effort to enter the experience without preconceived notions to manage their expectations.

"I worked quite hard to hold myself back from excessively googling information about trials and psychedelics and I had no prior experience of psychedelics, my partner does. And I even made sure that I didn't ask him about it, I really tried to make sure that I wasn't exposing myself to any information that could overly condition, my expectation of what might happen. " (P3).

P1 suggested that the challenge for study teams now lies in achieving informed consent that is balanced—providing enough information for participants to make an educated decision about their participation while avoiding the creation of preconceived notions that could steer their experiences in a certain direction.

“I think it's hard to avoid the lot of knowledge, increasingly, as the trials are running, there's going to be a bit of an expectancy bias, people are really sort of like, “oh, this is gonna work’… is that sort of like informed consent, like, how much do you say, to help people know what to expect without either guiding them or shaping the experience? It is a tricky balance.” (P1)

Despite the growing evidence based and positive media reporting about psychedelic research, participants entering psychedelic studies may still experience anxieties that might be rooted in historical cultural stigma, illustrated by P3 who had not used psychedelics prior to her participation in the study, and found herself both scientifically curious yet also influenced by ingrained fears around the dangers of psychedelic use.

" For me, it was both a driver and a fear as I was completely naive. But my only association I guess with with psychedelics was a sort of kind of childish fear, you know, I sort of am I was very interested, since doing the trial has had, you know, read and watched a lot more about the sort of history of psychedelics, particularly a popular book about psychedelics. And I think I certainly was one of those people who had been somehow influenced by this sort of cultural idea that you the risk of taking a sort of psychedelic with you that you'd lose your mind forever, or you jumped a building or something like that, you know, and kind of did have this slight fear that, is there a risk that I will lose my mind even more actually become madder, like that's, that's a slight fear. What am I getting myself into.?" (P4).

### 1.1.2. Challenges to expectation management during recruitment and screening

#### 1.1.2.1. Uncertainty around eligibility

All the participants expressed that they found the screening process for their trials stressful, due to prolonged uncertainty around eligibility, and P1 remarked that at times it felt like screening teams were trying to exclude participants from the study rather than include them, which keeps participants, who in most cases don’t have access to full inclusion or exclusion criteria, guessing about what the team is looking for.

I certainly felt during some of the interviews I had with them that I was trying to make my case. But it’s a fine line. I was trying to second guess, “What is it that they’re looking for?” They’re looking for someone who needs this, but is not so unwell that they would be taking a massive risk. (P1)

Other participants had similar experiences of finding themselves guessing about which responses to screening questions could exclude them from the study that they were hoping to get onto, reflecting the tension between demonstrating a need for the trial and not presenting with a risk profile that could preclude participation.

“I certainly felt during some of the interviews I had with them that I was trying to state my case. But it’s a it’s a fine line, because I would also like, well, I don’t you know, I was trying to second guess.. Well, what is it that they’re looking for? They’re also looking for someone who needs this, and but also is not so unwell, that they would be taking a massive risk by now, it’s a bit of a, it’s a bit of a fine line, really.” (P3)

These experiences underline how fears about being excluded from the study can lead participants to minimise reporting certain symptoms, which could then contribute to anxiety about these symptoms manifesting during the study or particularly in the dosing room—a setting where trust and safety are important.

“we’ve got a lot to learn from that recruitment (...) including 48 hours out, I’m getting a call saying that they’re missing a signed piece of paperwork from my GP, and I can’t go ahead unless they have that signed a piece of paper… I phoned the GP and they said, “well, it takes 72 hours and we’ll do this.” I’m like “I don’t have 72 hours.” Yeah, I just burst into tears.. “like somebody’s got to help me.” And I just went to the practice and I was like, at the counter… Can someone just sign this paper? And they went the extra mile … at the end of the day at 9pm… it was just chaotic. I shouldn’t have had the recruitment team (…) making It my problem (…) that just really spun me out actually.” (P1)

#### 1.1.2.2. A prolonged screening process

Whilst taxing, the extensive screening process may also raise participants hopes and expectations regarding the trials potential impact, as the regular contact with the study team contributes to increased anticipation, emotional investment and developing relationships. P3 describes growing enthusiasm and expectations throughout the screening process.

“And so my screening seemed to kind of go on longer than... because I was given some information about, you know, what the kind of screening process would look like, literally, oh, you’ll have this session and this session, and this meeting, and this meeting, and then we’ll decide. And then mine just kind of kept on going. So again, there was this kind of anxiety, like, I know, I kind of the longer it went on the more I was like “I really want to do this”. It started off with a kind of “Oh, wouldn’t just be interesting?” and then, you know, few weeks, four months online, you’re like, actually, this could be incredible.” (P3)

My desire and desperation to take part in the trial grew during the recruitment process, because I just sensed that this was going to be very different to anything I’d ever experienced before. And that increased my appetite even more. By the time I’d spent a couple of months going through the screening process, I was desperate to be accepted, and I would have been absolutely crushed if I had been refused. (P3)

### 1.1.3. Managing expectations through careful communication

During the focus groups, participants emphasised the role of communication throughout the phases of the study, but especially around the screening process. Effective communication, characterized by clarity, consistency, and empathy, was deemed essential, taking into account the impact of the dynamics on the mental health of the trial participants.

#### 1.1.3.1. Turning down the hype

Participants commented on the necessity for the research team to temper expectations, suggesting a need to balance hope with realistic outcomes. This management is important to prevent participants from developing unrealistic expectations about the trial's potential benefits, which could influence their experiences and responses to treatment.

“I think whoever's, you know, the people who were looking after the trial who were interviewing the people at the screening sessions, I think they need to maybe, I don't know how they could do it, but try and turn down the hype a bit. Just trying to, you know, this could work, it might, you know, not trying just to put the just put the people on level footing, so they don't have sort of unrealistic expectations or, you know, because I think that would drive part of your experience.” (P4)

#### 1.1.3.2. Shifting the tone

A shift in the tone of communication was noted as participants moved from the recruitment phase to the preparation stage, illustrating how expectations can be managed through careful communication on an implicit level. Initially, interactions were more formal, focused on assessing the suitability of candidates without creating false hopes.

“in my experience, conversations shifted ever so slightly.. I think whilst I was still in that recruitment phase, I kind of could tell that it was still very much like ‘we are just trying to work out whether you're the right person for this’, and it was still a little bit, it was just a slight distance… they have to be cautious because they don't know yet whether they're accepting you. So there is a point in time, I guess, when they make the decision to actually accept you for trial, then conversations become a little bit more practical become a lot more sort of thinking about tools… I just I noticed a slight shift, I guess. And you've got to be careful that you're not giving anything away in that recruitment stage and getting people's hopes up falsely. Just to be cautious about that.” (P3)

#### 1.1.3.3. Transparent timelines

The experience of awaiting a response from psychedelic study teams during the screening process has been a significant source of stress for participants. The often prolonged and opaque timelines can exacerbate the conditions of individuals already struggling with mental health issues, emphasising the need for acknowledgment and expectation setting in terms of timelines.

I think there's scope for better communications. I applied and it was nine months before I heard anything… We’re reaching out in desperation, so just even an acknowledgement or setting expectations in terms of timescales would be really helpful. (P1)

“It was similar for me, as well, as it took ages to get to the to, I think I got an initial response, saying, "Oh, you'll hear back from us." And then that is what took so maybe that took a few weeks to get that email back. And then I had nothing for … about nine months. And really, you know, you're dealing with depression, and anxiety. So that kind of.. and you're desperate. And none of that really helps you with that. It can make it worse, you know, when you're waiting…” (P2)

Participants agreed that a schedule should be communicated to applicants, providing approximate timescales for the screening process, to mitigate the anxiety of waiting without information. Such clear and transparent communication is important to managing expectations and reducing the uncertainty that can compound existing mental health difficulties.

“I think this should be like a schedule sent out, you know, like, once you've applied this is, it's going to take this long for us to get to your screening approximate, you know, approximately. So you're not just sat there sat there, like month after month thinking, Well, you know, they're gonna reply, or what's what's happening, and I can't remember how long I think mine was about six or seven months. And it was a, it was a long, wait to find out.” (P4)

However, when participants finally did hear back, they were invited for a screening shortly after hearing back from the research team, and the sudden shift from a prolonged wait to rapid engagement with the study felt overwhelming for some, as they shifted from a state of anticipation to immediate involvement in the study's demands.

“…but when it did finally happen… and it was really, really, then it was really quick, like the turnaround was really fast. And it was like the opposite end of that kind of scale of like waiting, waiting, waiting to Oh, God, this is like, it's all happening now.” (P2)

“So yeah, just … having an idea of the timescale, the flow, how things should work. I mean, that should be really part of the part of the model, hopefully, because you're dealing with people's welfare, people's mental health, whatever condition they're living with, you know, that whole process can, can make things worse in some ways.” (P2)

## 1.2. Importance of adequate support during the screening

### 1.2.1. ‘Set and Setting’ starts with first contact

Participants highlighted first impressions, noting that the process of creating a supportive and trustworthy setting begins the moment potential participants first interact with the research team. This could be during a phone call, a meeting, or even through routine communications such as emails.

Someone was mentioning the setting and how important that is to the experience on the dosing days, I think it's crucial that people understand that the creation of that setting begins with the first contact with the potential participants. They may or may not end up on trial, but if they do, this is the beginning of your relationship, your trust building. It starts even when you're just going through some boring paperwork. (P3)

Further, participants suggested that recruitment and training programs for research teams should explicitly focus on the development of trust from these first interactions. They pointed out that every aspect of communication, even standard procedural exchanges, contributes to the building of this relationship.

“I think the other thing that would be valuable to be incorporated into any training for the recruitment team is that it's just an understanding that your relationship, and you have got this as a team, trust-building, with your relationship with your participants starts from the first contact. So for anyone that you're meeting, it's remembering that you know, this person walks into the room or joins your call, it's your first part, first time meeting them, they may or may not end up on trial, but if they do, this is the beginning of your relationship, in your trust building. And I, you know, I partly attribute the value I got from the dosing, eventually, to how deep I was able to go and I linked that to the trust I felt and the safety I felt in the room which I linked to the guides and the the trust that I did build in them through the conversations which was started all those months earlier without but was something about the way they spoke to me, their compassion, their sensitivity, and it starts with those contacts. It starts even when you're just going through some boring paperwork, like everything about the trial.” (P3)

Poor responsiveness or perceived indifference could leave participants feeling neglected or anxious. Participants described early communication as part of trust-building, not as a purely administrative stage.

“Everything has meaning and it's understanding that the patients or participants whether their consciousness of conscious of it or not, are making meaning of the basis of everything so like email is a perfect example you send an email saying I haven't heard anything in a while I just want to check progress on my screening. If noone will get back to you for several weeks, no one acknowledges your email, - you as a participant feel abandoned, rejected. You interpret that as a lack care, interpret it as a lack of interest, it increases anxiety. You don't want to push it in case am I being too pushy? Its meaning making, constantly happening.” (P3)

### 1.2.2. Support and sufficient time to complete scales and questionnaires

The screening process for research studies can be emotionally taxing for participants. The nature of the questions often delves into sensitive areas, potentially bringing up challenging emotions.

“it's about the whole team understanding that it's not a tick box exercise, there is an emotional load. And therefore, you know, the next time you have a meeting with them, you ask the question, or how you know, the service. Okay, how are you getting on with them? You know, just like reflecting back, I understand because you that might be a bit taxing and draining, Did anything come up for you?”(P3)

Additionally, flexibility in the timing of completing surveys and scales can be beneficial for participants' mental health. Allowing for a buffer period acknowledges the unpredictability of life events and reduces additional stress for the participants.

“building in a bit of a buffer as well, that when you need those surveys in, I was generally pretty good at getting them in on time. But there were a few situations where, you know, life interrupted and they were a bit late. But luckily, the team were very they were like, it's okay, we didn't need them quite soon. But, you know, we've got a bit of time. And that was helpful just to know, that wasn't, I wasn't being sent the survey with an expectation it has to be done to be done.” (P3)

### 1.2.3. Communicating screen failures

Some of the participants of the focus group had been going through the screening process for studies for which they were not deemed eligible, and recounted their disappointment, but also worries about the reasons for not getting into the study. When they sought help from the screening teams, they found it was difficult to get detailed information about the reason for failing the screening.

It felt like a very distant, administrative team, rather than a team of caring people that wanted to look after you and make sure that you were you were okay….I knew why I'd failed screening, but not the detail, not the nuance… and I needed that information to know not to apply for other similar trials, for instance, and it took ages to get that out of them. I was obviously very disappointed, not to be able to take part in the trial, but that was all compounded by the sheer admin. It just felt very chaotic. (P2)

Participants valued when the study team took the time to explain the reasons behind a screen failure, articulating why certain criteria could not be met. This level of detail, communicated with empathy, not only serves to clarify the diligent nature of the screening but can also reinforce the importance of safety and suitability for the study.

“Clarity, consistency, really just communicating with you, you know, on a level having a conversation with you. I think it was, we had various emails going back and forth. And it was different people in the team, so there was no consistent thread. But I think it was he got to a point where even they were like, ‘right, well, we need to actually speak to him’, it was only a five minute conversation, I think, but to have a conversation about it, and the clarity that I needed. And when I was really disappointed at least I wasn't sitting there with the anxiety of like, oh, it could have been this, it could have been that. Really, really difficult.” (P2)

Participants also highlighted the need for this communication to be therapeutically informed, comparing it to delivering bad news with compassion.

“I think that should be really therapeutically informed, it needs to be the same as you know, "what kind of conversation would you have with someone if you were delivering bad news?" That's really on. It's not just a kind of: "Oh, we're sorry. Sorry about that. You're not on trial. That's the end of this. Here's some signposting. Goodbye". It's we've got time I'm giving you, you know, an hour, let's spend some time talking about how that's landing you. Yeah. Why? And like, wherever you go, you know, where you go from here. What can we do to ground yourself?” (P3)

The participants' understanding of this thoroughness underpins their trust in the process and, when coupled with clear communication about the reasons for exclusion, highlights the treatment team's dedication to patient well-being and trial integrity.

### 1.2.4. Meeting disappointment

Participants underlined the importance of sensitive communication and support for those who are not deemed eligible for research studies. The emotional impact of rejection can be significant, especially for individuals with severe mental health issues who may view the opportunity to participate in a trial as a last resort.

Rejecting a candidate from a study should not be a mere directive to return to prior mental health support, particularly when such avenues may have been exhausted. Instead, it is important to validate the applicant's feelings of disappointment and reinforce that this is not necessarily the end of their options.

Moreover, the team should be mindful of the fact that for many with mental health struggles, being declined can feel like a deep personal rejection, furthering negative self-perception and potentially worsening their mental health.

Participants highlighted the critical importance of addressing the disappointment of participants who do not pass the screening process.

I think you have a duty of care even from the very beginnings of that recruiting process, you're working with people who have mental health issues… People are coming to you in a state of desperation of feeling like it's a last resort… you have to be sensitive to the impact of disappointment if people aren't accepted. (P3)

“I would really be thinking about how can I sensitively deliver this news if possible… you actually really do own time to sit through sit and think about their next steps as well. Again, it's completely unrealistic to say, ‘oh, yeah, no, just just go back to your mental health team’… you can encourage that, but the reality is, for a lot of people, they may have already tried that, which is precisely why they've looked into the trial in the first place.” (P3)

“I think it's reinforcing messages around fully validating the disappointment they may feel, validating the fact that they may have perceived this as being their last option, but trying to reiterate that it doesn't necessarily has to be their last option, just reinforcing the fact that they're still on a journey, and that there's still the possibility of recovery in other directions, and that this may have opened up ideas for what a recovery journey might look like. But it doesn't have to be, you know, doesn't have to be that the door has closed forever… empower people to begin that thinking around self-management in the same way that you'd want to if person was accepted onto the trial.” (P3)

“This is this is where you, you really need to understand the kind of mentality that a lot of people struggle with, who have mental health issues where someone declining you for the offer of something that you know, could be supportive, can feel like a very intense personal rejection, and, by implication, a suggestion that your needs don't matter. Yeah. And I know, that may sound like a stretch to you, but I've got, you've got to understand the mindset that someone who's depressed and anxious will be in. And hate, you know, going through conversations where you you're sharing personal history of sharing the struggles, you're sharing the mental health and history of contact services, and then at the end of it saying "thanks, but you're not eligible. Go on your way." You know, the kind of the, it can, what that can, the way that it can land with someone who's in a depressive mindset is, you know, we don't really care. "Your condition isn't serious enough. It's not concerning enough, you yourself aren't a value to us," like, all these things can start to feed into your own negative mindset.” (P3)

### 1.2.5. Signposting for non-eligible participants

An essential part of the sensitivity about the potential disappointment of not being deemed eligible for the study is understanding the sense of desperation based on having tried other available treatments, and the sense that there is ‘nothing out there’ that can help participants, which again contributes to high hopes for study participation.

“I mean, if you had all the money in the world, yeah, provide anyone that fails screening with an hour long therapy session, you know, aftercare support, it's, it's all good and well, to say, "we will refer you back to your NHS services, but for most of us, they don't exist". Yeah. But I know that's, yeah, money wise, that's probably impossible..” (P2)

When patients fail the screening for clinical trials, it's important they're not left without options. Sign-posting to other services or charities ensures that these individuals continue to receive the support and care they need.

“So you've got to just work out how to convey those kinds of tools to people and potentially some signposting you know, if they have if they are coming to you with particularly particular diagnosed issues, can you signpost to charities that might be able to support or for people if they may need to talk to someone else?” (P3)

Many patients may not be aware of services available to them, especially if they have previously relied primarily on primary care or public services, which may be limited or inaccessible. By providing information about other organizations and support systems, researchers can help participants navigate their options and find alternative sources of help.

“I think it'll, it'll, it's hard to get on these trials. And I was thinking to what you were saying about recovery journey, I think part of the message is, if you don't get on to these trials, you know, just some kind of awareness of the movement in the industry, and then on route to being properly offered medications. And, you know, if you don't get on to the trial, there's still, you know, down the line, just just to know that it's not like your only chance at ever accessing legal treatment.” (P1)

Although this should be done carefully not to create false hope, it could be appropriate for researchers to offer the perspective that as research moves forward, there migh be potential future availability of treatments that may not be accessible now.

“I think yeah, sowing some kind of real hope, not false hope, you know, not not just kind words and good intentions. But yeah, retaining that hope, when you're screened out is really, really important. I think for me, it's like I didn't go into that screening process with a sense of entitlement.” (P2)

## 1.3. Optimising Recruitment and Screening

### 1.3.1. Involving partners or support network in screening

Incorporating a participant's support network, such as significant others, into the screening process from its inception is an approach that recognizes the link of individual well-being and interpersonal relationships. In one instance, a participant's partner was actively involved in the screening, offering insight not only at the commencement but also throughout subsequent phases of the trial, including post-intervention evaluations.

“my partner was kind of also involved in that screening, Just just just to sort of make clear, I suppose that like, my partner was sort of slightly involved in that screening process as well. So they sort of interviewed him and they interviewed him throughout all as as kind of part of the way of evaluating if he was effectively also part of the kind of evaluation on on the impact of the trial too, so sort of later stage they interviewed him, I think, to discuss whether he'd noticed any changes since since taking part in the doses. So he was kind of part of that kind of pre during post evaluation as well, which I think was quite interesting.” (P3)

### 1.3.2. Reimbursement for travel expenses

“I couldn't have taken part, you know, if that hadn't been the sort of travel reimbursement? You know, and I've made that clear. And that's why I was sort of pessimistic about me being allowed to take part in the trial. What Yeah, that did come through. (…). And I think that sort of restricts things a lot for potential participants. Because obviously, you know, like, six, six, lots of trains or return train travel to London, does add up to quite a substantial amount.” (P4)

Reimbursement of travel expenses is a critical factor in ensuring accessibility and minimising barriers for participants in clinical trials. Participants have voiced that while reimbursement is helpful, the requirement to pay costs upfront can be prohibitive, especially when substantial amounts are involved and when reimbursement processes are lengthy or complex.

“And they were saying, "Oh, well, we'll pay your expenses". And I said, Well, that's great. And then they said, but you'll have to pay it all yourself, and then claim it back. And I said, Well, how long does it take to get that back? And they said it could take up to about six months. And it was something like 800-900 pound or something? And so, you know, in total. And in the end, a senior investigator paid at all for me with the understanding that, you know, I would claim it back and then reimburse him once I had claimed it back, I would pay him back. And that was a bit of a bit of a mess, to say the least. But it it didn't, you know, it did allow me to to part but it added to the sort of the stress and the pressure of you know, getting being prepared on time kind of thing.” (P4)

A more streamlined system for managing travel costs, such as providing prepaid travel cards or directly arranging transportation, like taxis, could alleviate the financial and administrative burden on participants. Such measures would enhance the overall accessibility of the trial by removing upfront financial barriers and simplifying the process of engaging with the trial.

“it's all part of accessibility, I think, facilitating access to the trial and I feel strongly participants shouldn't be expected to pay for their own travel. it wasn't an issue for me, because I live in London, so I didn't really have far to go. And on the dosing days, they, they paid taxies, they arranged that all themselves, so I wouldn't I, you know, it's a real pain in the ass as well, having to kind of claim expenses back, especially with persons who are not familiar with it. I, I also am not 100% keen on people, the onus on people having to take responsibility of that themselves, you know, yeah, trial response, if, you know, effectively, like, if, if you're thinking about it, in terms of the clinical trial, you know, people are voluntarily offering to take part in these things. So it's important to think about how to facilitate that access.” (P3)

### 1.3.3. Information for GP and family

The provision of comprehensive information to the families and support networks of clinical trial participants, as well as to their primary healthcare providers, is a important element in establishing a holistic support structure. It enables partners and family members to understand the intricacies of the trial, including its objectives, procedures, potential benefits, and associated risks, which is particularly important when the trials involve interventions that are less familiar to the public, such as those with psychedelics.

“You've just made me think I think there's something even before the trial for the partners, but there's maybe within the recruitment kind of process or information, there's information for your partners, because I would be more cautious of my partner doing something than me doing it with the because you want to protect them, don't you? So I think there's probably something in that as well around what information can be shared with partners so that they understand the process of it?” (P2)

Additionally, equipping general practitioners with detailed information ensures that they can more effectively monitor and support the participant's health, both during and after the trial. Such measures not only facilitate a collaborative care model but also underscore the necessity of transparent communication channels among all stakeholders, ultimately contributing to the participant's sense of safety and well-being throughout the research process.

“It's definitely the information is the key element. I mean, my my partner had experience with psychedelics himself. So he wasn't, if anything, he was the one who desperately wanted me to do this, because he could really sense just how powerfully helpful it had the potential to be. So I think he was, you know, I'm sure he was sent him some kind of information. I really can't remember what I think it was probably slightly more around like, guidance on how best to be supportive to me in the aftermath. Yeah, I think information sheets are useful.” (P3)

# 2. Preparation

## 2.1. Therapeutic alignment and building trust

All participants had navigated lengthy and often arduous journeys through the mental health care system due to the treatment-resistant nature of their conditions and had engaged with various forms of psychotherapy prior to their participation in the psychedelic studies. They reflected on the similarities and differences between the therapeutic approach of their guides during the preparation sessions versus the previous experiences they had with ‘traditional’ therapy.

I have had contact with mental health services for 18 years, I've been in and out all kinds of different psychiatric institutions, I've been in inpatient units, I've been on multiple forms of medication. I've done CBT, dialectical behavioural therapy, MBT… I've done so many different things, which are all NICE guidelines approved, traditional forms of mental health treatment. And over the years, I have found all those experiences increasingly frustrating, they were scratching the surface, they were constantly putting a sticking plaster on the most enormous deep wound, and it was just not doing the job. So the idea that I will be trying something so radically different, is very appealing. (P3)

### 2.1.1. Therapeutic alignment

The psychological support offered during the preparation sessions of the psychedelic study was reported by participants to share similarities with traditional therapy in terms of the engagement with a qualified psychologist and the exploration of emotionally charged personal history. This included the sharing of painful experiences and current challenges, met with the expected therapeutic responses such as compassionate reflection, validation, and skilled questioning.

“They did feel similar to therapy in that I was sharing with someone who was a psychologist or some kind of psychological qualification, can't remember the exact qualification. I was sharing with them, painful, emotive historical stuff of my childhood or my current challenges. And they were very skillful in doing the usual sort of compassionate reflection, reflective questions, reflecting back my feeling, validation, all the things you'd expect from a skilled therapist, I suppose the difference is there wasn't necessarily a great deal of time given to try to unpick and analyse. And, you know, it wasn't really like that. But it certainly felt therapeutic, because it would have been pretty unprofessional, I think, and unethical just to ask me to dredge up a load of emotional stuff, and then to just say, "Okay, thanks. Alright, see you next week," you know? There was some level to try and help me deal with that.” (P3)

Participants also reported a sense of ease and openness in discussing personal matters, suggesting that the preparation phase was conducted in a manner that was understanding and non-intrusive. The experience was described as relaxed and devoid of pressure, contributing positively to setting the stage for what would transpire during the dosing sessions.

“And I don't think I was I certainly felt at ease and able to discuss sort of deeply personal matters with them. And it's, I'm not sure I'd, you know, I've looked back on this before, and I was thinking, Well, you know, what will? What was what was different? And I think it's just, you know, that they were listening to it, and the were understanding of the situation, or situations, and they didn't sort of push me or, you know, I didn't feel like I was wasting anybody's time it was, it was very relaxed. You know, and I felt that was quite useful to set some background, you know, to what was going to take place.” (P4)

#### 2.1.1.1. Essential aspects of the therapeutic approach

“And it was nice just to, you know, I felt like I was being listened to when, you know, they were taken on board, what my problems and problems were, rather than trying to force fit them in, we're in something, you know, that they thought kind of thing.” (P4)

The non-directive yet patient-centered approach adopted in the psychological support sessions of the psychedelic study was highlighted by participants as fundamentally different from their past experiences with therapy. The guides focused on creating a personalised space, where participants felt heard and their issues were not conformed to a predefined therapeutic model.

Participants noted the absence of directives or interpretative insights commonly found in traditional therapy, such as being guided to explore specific psychological constructs or relational dynamics. The facilitators were described as companions in the process, akin to guides with knowledge of the terrain but without imposing a direction.

“And the reason it didn't feel like therapy to me is because they were guides. I didn't see them as therapists, they were a supporter walking alongside me here, not offering a cure, or a therapeutic intervention. They were guides, facilitators, supports, alongside me, people who have maybe a bit of a sense of the territory, they have a map, but they you know, they're not telling me where to go or what to do. There was no expectation, this wasn't going to be a big trip, a life transforming trip, "this is your one chance".. this was just a process that we were engaging in. And it's a process that starts a thing. It's the start of a process. So for me, like my psychedelic experience now is about an ongoing relationship, and a further deepening with this, you know, the same kind of guide, almost like an apprenticeship or lineage, you know, you become part of something much bigger.” (P1)

The approach was supportive rather than curative, empowering participants with the insight that they were the agents of their own healing—a stark contrast to the dependency dynamic often experienced in conventional medical care.

“I suppose what I was trying to say was it felt therapeutically supportive. But they weren't directive. They weren't making interpretations. They weren't saying, "Oh, well, I think this is actually all about your mom. And you really ought to be looking at this and looking at that, and joining the dots in this way". And I suppose, you know, if we're thinking about preparation, you've got to understand that anything you'd want to achieve as a guide within the dosing room, you need to think about that has ramifications for how you behave way, way, way before because everything you do in terms of interaction with with the client group, will be sowing the seeds for what happens later on, because of the way that psychedelics will just draw on your previous experiences.” (P3)

The most important insight throughout my process was that I'm the source of my own healing. I don't have to rely on others to give me the answers. That was the opposite of what I'd experienced in mainstream medical care, where I would constantly be thinking of therapists, doctors, mental health nurses, as the holders of some kind of magical answer that was going to cure me, going to fix me, that they were the authority figures that I was constantly deferring to. And the experience on the trial was so remarkably different to that. (P3)

To enable patients to have that kind of insight, they need to be operating as a guide, right from the beginning. They can't just leave it to when you're in the room that you take over. They would never tell me where to go. If I sat up and asked them a question like, "Oh, my God, I don't know what's going on", they were very skilful at not being overly direct. That kind of guiding role has got to be started at the prep stage. Because otherwise, if I've been constantly thinking of them in the same kind of therapist modality, it would risk feeling too similar to other mainstream experiences I've had. I would have casted them in that role of they hold all the answers. (P3)

“about continuity, you know, like, if you do do subsequent trials, I was really lucky, I had the same guide in both trials. I mean, it does get better than that, actually. So you know, and it was absolutely, and the comparisons that we drew between the two experiences, it was absolutely a deepening, and there could have been all lost if it was a completely new set of probably equally as amazing guides. So I could build trust with and I was really gifted with the opportunity to go even deeper, and with with the same guide, which again, makes me feel about the importance of having that sort of continuity and ongoing support.” (P1)

### 2.1.2. Importance of building trust

I think it was a reflection that the preparation was done really well, I had developed that trust. And even on the first dosing, it became really intense. I think I had let myself go I, I felt that trust. (P3)

Participants described trust as central to preparation and dosing. It was linked to their ability to disclose difficult material, feel safe during the session, and engage with challenging experiences.

During my dosings I went to some pretty dark and difficult places. I only did that because I felt safe enough to, because of the relationship I'd built up with the guides. That relationship started six or seven months prior to actually having the doses. The therapeutic relationship doesn't start the moment the person has taken a substance and is lying down on the couch, it starts way beyond before that. (P3)

#### 2.1.2.1. Building trust takes time

Building this trust, as participants noted, cannot be rushed—it is a gradual process that commences from the initial interactions during screening and continues to develop throughout the preparatory sessions. These early engagements, while not explicitly framed as therapeutic, often require a deep and vulnerable exploration of personal history to inform the guides on how to best support the participant during the dosing.

There is that time aspect of “I've only known you for two weeks, and here I am laying myself bare and being completely vulnerable in your presence”. For me, that's an issue anyway, because of what I went through as a child with the abuse, and all of that side of things. (P2)

Participants have expressed the desire for these secondary sitters to be present throughout the process, allowing the same level of trust to develop, therefore ensuring a uniformly supportive environment during the psychedelic experience.

“I had a brief introduction to my secondary sitter. And I didn't have that level of trust that I had with my main sit with her. And I know why you can argue, or maybe they're just there for when the other sitter goes to the toilet. I really think if it's possible, and if it's affordable, that secondary sitter needs to be in every session as well, if at all possible, because you need to have that same trust and that same bond with them.” (P2)

With the initial conversations, they're trying to build that important bond of trust between you and the team, within just two or three sessions. And it's not framed as a therapeutic session. It's framed more as getting to know you. But you go on a really deep dive, you dig up a hell of a lot of stuff, because that's how you give them an idea of how both you and they can best navigate what might come up during the sessions. (P2)

#### 2.1.2.2. Exploring personal history contributed to building trust

Exploring personal history during preparation sessions serves a dual purpose in the context of psychedelic psychotherapy. Firstly, it aids in the establishment of a meaningful intention for the upcoming dosing session.

“It felt like that the purpose of having these deep conversations was in the interests of working towards an intention setting type conversation, which is another thing we did prior to the doses. And I suppose what you'd want to say to people is: in order for us to collectively support you to identify a really powerful and meaningful intention, it's useful for us to collect together get a good idea of the background, what you're working with, where you've come from what you're dealing with inside. And in order to do that, it may mean that we have conversations which are potentially painful, bring up difficult things.” (P3)

Secondly, and perhaps more fundamentally, these explorations build a relational container of trust. Participants expressed that by sharing their life stories and critical personal details, a sense of safety and connection with the guide was cultivated.

“But the first trial, the prep was really intense dives, you know, you're kind of trying to convey everything about your life that's important in quite a short amount of time. And it's very, very intense, and it wasn't framed as therapy. It was framed more as a chat, really, but it's kind of like, you're trying to go, here's my life story. This is everything. These are the key things about me, you need to know, that may come up. This is how, you know, here's a map basically. So it was quite intense. Although I did like, you know, there's a member of the study team, I think a member of the study team was the main person who I had contact with at the start. And he was really supportive and really nice. Yeah, so those were quite intense. But I felt like they were really trying to develop that trust. And that was so important to do, and that safety that you desperately need to feel as well within that environment.” (P4)

The trust established through these preparatory discussions assures the participant that they are not navigating the intensity of the dosing session alone but are accompanied by a guide who is not only knowledgeable about their background but also deeply invested in their well-being.

“For me, I felt very bonded or trusting of both of them. And that was certainly useful that I saw them as a kind of joint unit. And then when I think about some of the conversations we had, I suppose, you know, they were quite emotionally stirring conversations. I was so keen to get as much out of the experience as possible, that I was quite willing to be very open, you know, I told him, everything really, and went to the difficult places, even in our prep conversations. I'm a kind of person as well, if I'm going to do something, I will really fully commit myself to it. So I don't like to do things by halves. And no, they felt very, like trustworthy people. So I was quite willing to be open.” (P3)

#### 2.1.2.3. The entire team contributes to a trustworthy setting

In the context of psychedelic studies, the cultivation of trust extends beyond the direct therapeutic team to encompass all individuals involved in the participant’s experience, including the recruitment and screening teams. Participants highlighted the importance of feeling connected and comfortable with various team members, not just the primary guide or therapist.

“I think what I'm hearing, again, is that that trust is built not just with the therapeutic team, but like the whole recruitment team. You know, as I said, if I did my second trial first, I don't think I actually would have gone through with it. Because I knew the team I was working with, it's like I just needed, you know, muddle through this chaos. It's not indicative of the experience. I just need to get to the experience and sort of like, not think too much about this. But if that was my first experience, I probably would have dropped out, I probably wouldn't have stayed. So that, you know, the importance of training the recruitment team and the screening team, you know, the the non the non therapeutic team on the process is super important.” (P1)

“set and setting and creating the container that to me, it's actually not just about the clinical team, your therapeutic team. I think the recruitment team, I think all contact starts meeting me because I had such a diabolical recruitment. And I mean, even when I went in on the day, I rang, it was in Yeah, it was very Yeah, so I did one at a commercial site and one an academic site and the experiences were very, very different. So you get to the commercial site and, and you I've been funnelled through an entrance of there must have been about another 100 people going in at the same time for a different trial. They were healthy volunteers. And I get there and that the person at the door is checking everybody in says, you're an, an outpatient, and I'm like, "no, no, I've got a suitcase. I'm definitely an inpatient. I've got my suitcase". "No, no, I don't have you as checking in for overnight stay", "like I am definitely checking in for overnight stay". And I was like, this is just insane. I just want to walk away I just like every every and this is after the paperwork bungle. I'm getting bungles at the door to get in and being told I'm not coming in. I'm not being admitted. And like, I'm definitely being admitted, I'm like, why am I even having this conversation. And to me, you know, we're it not for the therapeutic team, my two guides who, I had the same guide on both trials. And then And then obviously, my, the psychiatrist was new, but just as incredible. Were it not for the fact that I had such a good therapeutic team, I would have run a mile. The this feeling of how the whole process was being managed, was was horrendous.” (P1)

The initial encounters with the research team can set the tone for the entire trial experience. One participant mentioned that familiarity with the team from a previous trial helped navigate the administrative chaos of a subsequent trial.

“I felt closer to the psychiatrist than I did to him. There was just more of a natural kind of rapport and bond there. And he was also key in helping me reframe the fact that the second trial didn't work would be the way I would have framed it immediately after. He was key in doing that for me as well. I'm really glad he was there, though, at least there was someone in the team that I had that bond that that trust with.” (P2)

Moreover, participants noted the disparity in trust levels between primary guides, with whom they spent considerable time, and secondary sitters, who were present for practical reasons but with whom they had limited interaction.

“It's interesting what you said about having that comfort with the whole team. And just thinking about the one trial, my second, my second trial, I would say that I had a lot more trust, built a lot more trust, I was a lot more comfortable with my psychiatrist and various other members of the team than I was with my secondary sitter, or whatever, chaperone, whatever you want to call them. They're there when the other person wants to go to the toilet or whatever. And I had barely any time with her to get to know her to build that trust. And I really think that, yeah, I had that trust for several other people within the team, but not her. And, yeah, that was really, that was really important, as well, I mean, the cold didn't help either. But it's like, she really did her best. Don't get me wrong, I don't dislike her. And she really, she still showed up for me to be there on the day. But I think they could have done a lot more, I think, ideally, to have her there throughout most of the sessions that I had with my primary guide, I think would have really helped me.” (P2)

#### 2.1.2.4. Influence of the gender of the therapists

Participants discussed the gender of facilitators as relevant for some people, particularly where there was a history of abuse or difficult parental relationships. They also noted that initial preferences could change once trust developed.

One participant shared an initial resistance to working with male therapists due to a strained relationship with their father. Despite these reservations, some male therapists proved to be significantly beneficial, challenging the participant’s preconceptions and contributing to personal growth.

“when I first went into mental health treatment, I was adamant that I was not going to benefit from any interaction with a man, I've not got very good relationship with my dad. And I was just basically like, Well, I'm just gonna have to work with female therapists. And I didn't have a choice, I had a male consultant doctor, who ended up being one of the most important people in my mental health journey ever. And some of the most important and helpful therapists I've ever had, were men. And they were men who in my first few sessions, I was like, you are going to irritate the hell out of me, not because of a man, but just who a man is one that had, he wound me up so much, something about him I just found so irritating. We ended up working together for two years in a hospital. And he was really important to me, it was a really important figure. And I can think of other situations where I've been in therapy, where there's been something about therapists, which has kind of rubbed me the wrong way. And actually, it's been kind of important, because there's, you know, why are they rubbing me the wrong way? And what is it about my lived experience that they're bringing out that actually needs to be worked through?” (P3)

Furthermore, participants acknowledged that therapy might involve discomfort with certain aspects of a therapist’s presence, which in itself could be an important part of the therapeutic work.

“those relationships evolve. If you have more than one dose as well, they evolve over time. I think I wouldn't have chosen the whole a full male support team for me at all, I would have gone right need females don't be just men because of what's happening past web. But like you said, that was part of my lesson was like trusting a group of very, you know, empathetic men to hold the space for me.” (P2)

“I'm not sure about this one, because there's part of me, which thinks, yes, people should have a right to say, you know, let's imagine a situation where someone has a history of sexual abuse, right, that's from a male and they say, I would rather work with a female guide, because I think at this point, it's just going to be too disturbing, for me to go into this space with a man I I'm not comfortable.” (P3)

“psychological safety to to, if you can develop the trust, then you can have an open conversation about, I'm struggling in our relationship and something about you that's, and that happened with me. So I was very close to my guides, but I struggle a lot with with self confidence. And there was, there was something about one of my guides, particularly comparing myself to her in a really like, self critical way that actually one point just got really intrusive. And they were so perceptive, they, they kind of picked up on it. And we had a really great conversation about it. And it was really helpful. It was really important. And that's my stuff. That's my shit. You know, you could also argue, well, it was kind of good that I had someone like that, yeah.” (P3)

## 2.2. Learning to navigate the psychedelic experience

### 2.2.1. Balancing information with expectation management

Participants in psychedelic studies highlighted the delicate balance required when conveying information about the study during the preparation phase. They stressed the importance of not setting specific expectations about the dosage or the potential intensity of the experience.

“They really gave try to give an accurate picture and try not to, but I think, again, the influence comes externally, you know, the media articles. They're not as balanced. But I did think that my clinical team were quite good at trying to say: ‘it might not be like that’”(P3)

Participants were aware that suggestions during preparation could shape expectations. They valued neutral information that acknowledged a range of possible experiences without implying what should happen.

“I think it's problematic when maybe people are putting ideas in your head. And I was thinking this earlier, actually, when P4, you were talking about how you've been told, "Oh, prepare yourself, this one's going to be a massive dose" like, that would have really screwed with my head. I mean, I was not told, I was never told.. never given any indication at all about what the doses were going to be like they always said that you might have a profound or quite significant experience, but you might not feel very much. And they said that before every dose, so I never had a sense of whether it's going to be a small dose or large dose, or what my experience might be. Because I think when you start anticipating and expecting and forcing something, I mean, I'm not an expert, but to me, it seems like that's where it can become problematic.” (P2)

This careful management of information serves to create an open space for the experience to unfold naturally, without preconceived notions of what the journey should entail. It also reflects an understanding that the psychedelic experience is highly personal and subjective, and pre-dosing suggestions or biases could unduly sway the outcomes.

### 2.2.2. Covering the range of possible experiences

Preparation included discussion of the possible range of effects, from little or no subjective change to intense or highly meaningful experiences.

“they gave us an overview of, without being too prescriptive and overview of the type and range of experience that would be possible. Including no experience for some people and a transformative experience for other people, you know, any experiences is an experience ultimately, even if it's a non-experience experience, and I think that really helped, especially if you're on a trial that has a placebo potential as well. Yes. So it's really important to say that there's a whole range of experiences including a placebo.” (P1)

“my experience of that was the the same, that that didn't try and influence in any way, really, you know, in terms of what to expect. It was just sort of, you know, if this happens, then, you know, I guess if you start sort of panicking, we're here to help you. You know, and it was just sort of things that that may crop up but not actual experiences, just sort of, you know, like it was more sort of medical really and psychological but no, that definitely didn't influence me too much and I think that's that's vital, really, because I think what people say beforehand, you can take in with you sometimes, into these experiences.” (P4)

Participants noted that the clinical team informed them of the possibility of a placebo experience or one that may not meet their initial hopes. This approach was seen as helpful, particularly in trials with a placebo element, as it prepared participants for any outcome.

“I was completely naieve to psychedelics and just trying to think back to how sort of the guide talked about it. I mean, looking back again, I think they were careful not to say.. "they didn't say you will see this, you will experience this, you will hear this, you will feel this." .. they spoke a little bit more obliquely about the potential way in which psychedelic substances can impact on I guess, the flexibility of the mind, I suppose, like it was much more like, the reason we are researching it is because that we are interested in the possibility of these these substances, having an impact on your plasticity and helping to, you know, my disorder is is is a lot about control, it's about obsessiveness, It's about very self-destructive patterns of behaviour, It's about very rigid patterns of thought, It's about entrenched ways of being. So within the trial that I was taking part in, the sort of angle in which they were trying to prepare me was very much around: the reason why we are interested in the potentially of these substances, is around allowing a level of flexibility, a level of liberation in your ability to make certain choices or in relationship with yourself. So it was a bit more generalised, which I think is really important. So the key thing is they weren't saying: "You will close your eyes, and you will see visions, and everything will be prisms and colours", they weren't sort of telling me what sort of experience I would have. They were more talking on that kind of deeper level about the potential.” (P3)

The guidance provided aimed to help participants understand that while they may have desires for their experience, such as substantial insights or cures, the reality may be different, and that in itself is valuable.

Moreover, participants appreciated when guides discussed the psychedelic experience in terms of its potential for increasing cognitive flexibility and neuroplasticity, especially when relating to personal issues like control and rigid thought patterns.

“think in my trials, they definitely spoke to the neuroplasticity and the cognitive flexibility, absolutely, which is kind of the sciency part you were mentioning, but mine also definitely mentioned the potential for mystical experience. So for me, they, you know, there was definitely mention that this is part of the range of the experience, and that can have an impact. So, and I think it felt important that that was was named I think, like, like P3. I mean, I think you can't avoid the psilocybin information at the moment. It's just out there, I think, because that was different for my DMT trial, because it's, I think, not as much out there at the moment.” (P1)

If you go into it thinking: “I'm going to be cured of my problem”, than it's a really hard experience to have, if you don't get that outcome. I think if you're holding in mind that there may be something of value to be got from whatever experience you encounter, that can be a helpful reframe. Not at all to suggest that bad experiences don't occur, they do, but it's just trying to set the person up for having a method, before they even do the experience, for reframing it if it's a challenging one. I think providing people with that tool as early as possible is absolutely essential. (P3)

### 2.2.3. Lower doses do not always mean less intense experiences

Participants in psychedelic studies have observed that the assumption of higher doses leading to more intense experiences is not always accurate. Their experiences highlight that the subjective intensity and therapeutic value of a psychedelic experience do not necessarily correlate with the dosage.

One participant described having a very intense emotional experience at a low dose, suggesting that dose level alone did not determine subjective intensity.

It's really important to not equate higher doses necessarily with more intense experiences. I had three doses. The first session was the lowest dose but I had an extremely intense experience on what was definitely a low dose. There was very minimal visual change. If I took my blindfold off, the room look pretty normal, but I actually had one of the most emotionally intense experiences in my entire life and released a huge amount of trauma during that time. I was literally screaming and howling and sobbing - like an immense amount of distress. I think I went quite deep and I think that says a lot about the trust I felt in my sitters and about the into the preparation and intention setting you've done beforehand. (P3)

Another participant recounted a contrasting experience where a higher dose of 25 milligrams, expected to be more intense, was underwhelming compared to a transformative experience with a 10-milligram dose.

“Yes, 10 milligrammes was what changed everything. And then the 25 milligramme was a quite a disappointment, because, you know, I expected something even more profound or something even stronger kind of thing. But it was very, very mild indeed, whenever the 25 milligrammes but that is because of the timing, you know, I was exactly a week afterwards. And I don't think that given enough time in between the dosing, I mean, it works for some people, but I was reading an article, I can't remember the science behind it, but it is a thing where people build up a tolerance fairly quickly. So that I think there needs to be a bigger gap between sort of initial and secondary dose. It was definitely more profound, the first one on the lower doors.” (P4)

A third perspective cautioned against equating a more intense experience with greater therapeutic value.

These accounts show that dose, individual history, set, setting and readiness were experienced as interacting factors. Participants therefore cautioned against equating higher dose with greater therapeutic value.

Just because the experience is more intense it doesn't necessarily mean it's a better or a more healing experience. Bearing in mind some people really need an intense experience, so they can let go and get through the resistance. But from a healing perspective, during my DMT experience, I didn't know which way was up for most of that, it was surreal. My psilocybin experiences are more autobiographical. I have a sense of me and space and time and what's happening and actually being able to revisit those experiences with a sense of which way is up. One should be careful not to fall into thinking that more intense is always better. (P1)

### 2.2.4. Research team setting the wrong expectations

A participant in one of the early psychedelic studies recounted a disappointing experience with their second dose, which lacked the expected intensity. This lack of effect was particularly disheartening due to the high expectations set by the research team during the preparation phase.

“It's pointless for me to talk about how the second dose was, there was just hardly anything there, I didn't feel anything. I just remembered, you know, way back in my sort of the teen years, when I'd had experienced psychedelics then, I remember the, the old sort of guy of the group, you know, the most experienced one. He was saying, you know, take all you want, you know, it's this is non addictive. Because, you know, within a week, they have no effect kind of thing. And I don't, you know, I don't know whether that played any part of it. But I was very disappointed in the second one, and, and some of it was because of the preparation between the two. The preparation was, oh, this is going to be so powerful. You got to prepare yourself. And, and it was a total anti-climax. And the study lead, did say in a meeting again a year later, that they changed that, they weren't going to do the week in between, because there was somebody else that had, you know, a negative experience on the second dose as well.” (P4)

### 2.2.5. Communicating that all experiences are welcome

Participants emphasised that all experiences should be treated as welcome and potentially useful for learning, including difficult, disappointing or low-intensity experiences.

One participant highlighted that the willingness of the research team to hear about all aspects of the experience—"The Good, The Bad, and The Ugly"—is fundamental for learning. This open-minded stance communicates to participants that their experiences, no matter the nature, are of value and contribute to the collective understanding of psychedelics.

“It's just a messaging system It's in the prep, and and it's get beyond just a kind of, oh, there's no such thing as a bad. It's like, how do you really help participant feel like, we're really keen to hear every part of your experience, The Good, the Bad, and The Ugly, because through that we'll learn and that's really valuable.” (P1)

Another participant pointed out the dual nature of this message: every experience provides learning opportunities, which are as valuable for the participant's personal development as they are for the study's outcomes.

“There's a dual message with that: it's not just like, every experience is valuable in terms of research, but every experience is a valuable learning. And I think this is a I have the luxury of saying that because, you know, my experiences were, although incredibly painful and emotionally intense. But you know, that was I wouldn't look back at them and say, of course, that was wrong or anything like that, you know, got good feelings about them. And that's, that's a privileged position to be in. But I I do feel like even if it hadn't gone... or if I didn't have a strong effect from it, or if I... if it is utterly terrifying, there'd be something to learn and that's where the good integration comes by.” (P4)

Furthermore, a participant mentioned the long-term perspective, suggesting that the true measure of the psychedelic experience's success might not be apparent immediately post-trip but may manifest over weeks or months.

The outcome isn't just about the dose, it's not about the trip, it's about outcomes convening in a week, a month, two months, three months time… because I could easily think that without any aftercare, the trial is a failure. “That doesn't work for me, I'm back to nothing working for me again”. But ultimately, it's served me really, really well. (P2)

### 2.2.6. Explaining the role of the guide

Understanding the role of the guide is an important facet of the preparation for a psychedelic experience. Participants stressed the significance of knowing not only how guides would interact with them but also what guides would be doing during the dosing session itself.

One participant recalled discussions where the guides explained their roles, including possible note-taking, but emphasised that they would be present and attentive throughout. This participant also acknowledged the potential for anxiety among guides concerning the impact of their actions and reminded that there is room for flexibility and unforeseen circumstances.

“But now that you mentioned it I do remember that there were some conversations where they would explain their role, and I think they also explained, "we may be, you know, at times writing notes, or, but we're here with you. I suppose one other thing that I want to say, I am mindful that, you know, if I was listening to some of the things that" we're all saying, as a potential guide, I might start to feel increasingly anxious, like, "Oh, my God, I like if I just touch someone in the wrong way, I'm going to absolutely ruin their experience" and I suppose one thing I want to say is that, like, there's always the chance that something might not quite go right, or there might be an unexpected situation.” (P3)

Another participant pointed out a gap in the preparatory information, specifically around what the guides would be doing while the participant was deeply immersed in the experience.

I got told a lot about what they would be doing to me and how they would touch me. They used a cabin crew analogy, that they were there to support me with whatever I needed, “We're helping you on your journey”. But I didn't know what they would be doing while I was journeying. That's the only missing piece. I found my facilitator at one point using a phone which felt quite unnerving. But reading, writing, taking notes felt okay… just mentioning that. (P1)

Moreover, the role of the guide was likened to a doula in childbirth, where a balance between attentive support and allowing space for the natural unfolding process is important.

“I think mentioning that as part of the preparation for what the sitters will be doing during the journey is actually an important point as part of the facilitator training. Because I've got mixed feelings about this, I'm so happy you prompted this because I hadn't thought about it. It's kind of like when you're giving birth and you have a doula the best doulas won't be nervously looking at you all the time. Like I felt a bit like I'm, when my sitters when I took my mask off, and they're looking at me to see if I need anything, I felt like I was a specimen in a petri dish. And I know it's science. And I thought, yeah, but I just need a bit more cocooning. I don't like them to look at me so much, right? So when I'm reflecting on the birth process, which to me, it's not too dissimilar process to this, you know, you almost need somebody who's I don't think they should be on their phones. Absolutely not. But I think somebody's just reading a book, and when they see you, they come away from the book and say they'll be just doing something gentle, knitting. To me, it's like almost like, because that almost gives me confidence. I think in the birth process, again, the medicalization of birth, you know, everyone's on top of you. And what you need is somebody who's just holding space and doing something gentle and nourishing. And allows the process to unfold because that's what's happening both with birth and with psychedelics, right, there's a process unfolding. And if you need something, they'll step in, you know, but actually, just creating that space has an intelligence. And you're doing your thing, and this is unfolding, as it should, as opposed to sort of urgency that if you just take your mask off this, people were looking as if something was wrong. And I'm like, "is something wrong? No, I'm, I'm fine." So I'm just yeah, just allowing that in terms of the training, it is such a subtlety of like, this is a process of unfolding and instilling that confidence in people.” (P1)

Participants described the facilitator role as involving both active support and unobtrusive presence. This balance was seen as an important part of facilitator preparation.

### 2.2.7. Feeling like letting down guides or research teams

One participant reflected on their experience in a well-organized second trial, noting the emphasis placed on setting an intention for the psychedelic session. Despite the guide's efforts to help them articulate a clear intention, the participant, feeling deeply troubled at the time, could only express a desire to "feel less shit." This inability to define a more specific intention led to a sense of disappointment, as the participant felt they had let their guide down.

“The second trial was very different. It felt more organised in many ways, and there was a lot of focus. And there needs to be a lot of focus on intention. And I had conversations with my main guide, and he really tried to get an intention out of me, but I was in such a hole, all I could come up with was, I want to feel less shit. And that is not a defined, a well defined intention, you know, and I really felt like I'd kind of let him down by not being able to provide that.” (P2)

### 2.2.8. Practical tools to learn to navigate the dosing experience

“But the other thing that they did, which felt different to therapy, but also was absolutely a method in sort of preparing me for the dosing was that we started to do breathing exercises together.” (P3)

Participants in psychedelic studies have highlighted the incorporation of practical tools during the preparation stage as important for navigating the dosing experience. A key component of this preparation was the introduction and practice of techniques to manage potential challenging moments during the psychedelic experience.

One participant specifically noted the introduction of breathing exercises as a distinctive method that differentiated the preparation from traditional therapy. This technique was practiced together with the guide, which not only served as a relaxation method but also equipped the participant with a coping mechanism to be used during the dosing session.

Another participant described the preparation as a two-pronged approach. On one hand, there were discussions about personal challenges and areas to work on, which set the stage for the dosing experience.

If we had a preparation conversation, they would often start it or end it with this very simple breathing technique, guided breathwork. And there was a kind of mantra, which was something along the lines of "I am here now", as a grounding technique. It introduced me to a technique to help me get through challenging moments during the doses. There was a particular moment during one of the doses where I became very stressed out and anxious. And one of them very quietly reminded me of the technique to bring myself back to a place of safety. So I thought that was fascinating that they were beginning to just provide me with some tools that I could later use by planting seeds. (P3)

By actively practicing these techniques, rather than simply mentioning them, participants were able to familiarize themselves with tools that could aid in navigating both the expected and unexpected aspects of their psychedelic journeys.

### 2.2.9. Breathing exercises and guided visualisation

“In terms of the actual prep, both of the trials had different guided visualisations a facilitator developed both and it was companied with sort of talking around the the experience, what I liked, is that it wasn't prescriptive, and hence that delicate balance of giving enough information, that you have an informed consent process, you know, kind of what to expect, but not so much information that, uh, guides or shapes or influences your experience.” (P1)

In the context of psychedelic research, participant accounts have highlighted the importance of preparatory practices such as breathing exercises and guided visualizations. These methods are not only ancillary; they form a important part of the pre-dosing regimen, serving to equip individuals with psychological tools to better navigate the complex terrain of psychedelic experiences.

“I mean, looking back that was so skillful with it, because I think they managed to do it in such a way. So it's not to make think: "Oh, my God, it's going to be terrifying. And I'm going to need to be doing endless breathing exercises". But it was just a sort of, like, you know, if things get difficult, this is a thing you can come back to you can always come back to your breath. That was a thing that was a phrase to use, but always come back to a breath, stuff like that, which is then when you're in the moment, they might then whisper in your ear, your resume and you're like, it's it's just linking everything together.” (P3)

Doing a body scan and releasing tensions in the body, especially for DMT which is a very visceral experience, was a really supportive tool. It’s important to put these things in, these little seeds, these tools that you can use to support yourself. Being given them as part of the preparation helps.” (P1)

“But the other two recordings, the voice was was one of my guides, which I found just again, super comforting, hearing the voice. It's just planting the seeds of security. So again, that when in the room, if things are getting difficult, and you hear that person's voice in your ear, they didn't interfere a great deal, but at times, you know, they would lean over and maybe say something to help me feel settled again. And I just am convinced that you know, I think it was having the familiarity of that voice is really powerful. You know, we also had a visualisation similar to what P1 was mentioning. I certainly did that on the day of the dosing, they sort of talked me through that visualisation. I think it was the one of the diver going into the depths of the ocean and being in the dark. But imagine eventually finding the surface, that kind of thing.” (P3)

Breathing exercises, including body scans, have been described as important for fostering a tranquil and present state, enabling participants to attune to their physiological responses and reduce any surfacing distress. Notably, these practices were not cursory mentions within the preparation phase but were actively engaged with, creating a sense of readiness and resilience.

“I just want to really emphasise a space, that kind of core part preparation is effectively arming the participant with some sort of inner techniques that they might be able to call upon. If things get difficult during the dosing, I think that is covered in our theme. But, you know, for me, it was really helpful that once I was accepted onto the trial, every time I had a meeting with the guides, for whatever endless conversation we had, there will always start with a brief breathing exercise, with repeating a kind of mantra, I was quite early on, given access to some kind of Guided Breathing things I could listen to. And like, it's fascinating. It's kind of the power of suggestibility isn't it? They weren't, they were sort of saying, Oh, this is just the way that we want to do this. And like, this is a really sort of checking in here, let's just check in with how you are, let's do some breathing. And, like, obviously, it was useful during the dose when it will get overwhelming with anxiety-provoking. I think the mantra for us was something like: "I'm here, now", - what's so simple, it was just about presence. And you know, that that was a tool to help me have these open and frank conversations that was really the primary the tool to help you to dosing when it got really frightening. So there's something about beginning to get the participants practising that kind of thing. And, you know, again, it's understanding that power of suggestibility, the seeds that you're already planting within people before they've actually got the dosing understanding that they have the resources within them to cope with things get frightening to, you know, understanding that when something dark and frightening emerges during the dosing, they can go through it, you know, it's not something they have to run away from the going through is the way to sort of face that all that kind of things, and lots of ways you can prepare people for that kind of mindset.” (P3)

However, the intensity of these visualizations necessitates careful consideration. Participants have recounted instances where the vividness and suggested adversity within visualizations contributed to a sense of apprehension rather than preparedness.

“And on that point, I think the second it's really, really a hard one, I keep tooing and froing on this on the second visualisation for the DMT experience, is like as a visualisation is a lot more challenging than going on a pearl dive, right? It was very much you're going up this very steep hill and it had a downward spiral. But you come out into the light at the end. But at that that idea of descent or it's quite a it's quite a it was quite a huge visual visualisation, it was kind of kind of intense visualisation. And my first dosing was a placebo. And I had a lot of.. not adverse effects. But my goodness, my heart rate went through the roof. I had like some serious like anxiety going. And I think it was partially because of the anticipation.” (P1)

“That's where I get torn because if you do such an intense visualisation, the the DMT experience is in incredibly intense, so I think I would have been sort of unprepared in some level if it wasn't as intense a visualisation. This wasn't a you know, gentle submersion to the bottom of the ocean with an anchor this this really does blow the door off the rails. So if I didn't have that kind of visualisation,with positive bits to it, I mean, it wasn't the negative visualisation. There were beautiful flower archers, and there's hope and then the jewels to find in the in the caves. It was very nourishing but I think I wonder if maybe it's just about the intensity of the experience compared to other psychedelics.” (P1)

The challenge for researchers is to strike an equilibrium in the preparation process.

Together, participants argued for a non-judgemental approach to the full range of possible psychedelic experiences, with integration used to explore what the experience might mean rather than to force a positive interpretation.

### 2.2.10. Clarity around the use of touch and session boundaries

The introduction and clarification of touch in the context of psychedelic therapy preparation is a important aspect of the preparatory phase, as evidenced by participant reflections. The deliberate demonstration and discussion of touch by therapists prior to dosing serve a twofold purpose: first, it preempts any potential alarm or discomfort that could arise from unexpected physical contact during a heightened state of consciousness; and second, it delineates the therapeutic boundaries within the unique setting of psychedelic therapy.

Participants spoke to the necessity of understanding what forms of touch were permissible and to what extent they could engage with their therapists physically. This interaction departs significantly from traditional therapy dynamics, where physical contact is typically non-existent.

“I remember now that you brought it up, just before I actually took the psilocybin I remember one of them explaining. Yeah, she literally demonstrated on on me how they would make contact to get my attention, what the touch would be like, If we need to get your attention for any reason, this is what we'll do. She explained, you know, we don't want to alarm you, so what we'll do is we'll gently touch your elbow or something. I can't remember what it was, but she literally demonstrated it.” (P3)

Discussions about session boundaries were also deemed essential. Participants appreciated being briefed on their ability to request assistance or engage in touch, which allowed for a more open and expressive therapeutic interaction.

“Even if you're you as therapists are trained, it's useful for participants to have a chance to talk about that, ya know, what we're allowed to do? The other, it's useful to know that we're allowed to ask for help, or we're allowed to ask for, you know, I was if I had a really helpful conversation around touch, and I thought it was useful for me to know that I could reach out for them. And I was allowed to do that. Because that's different to your standard therapy. So yeah, you know, if you have experience with mental health services in the more traditional medical sense, it is very clear that I'm not allowed to reach across the room and ask for my therapist's hand, and that's different in the psychedelic space. So even knowing that is useful. Oh, okay, I'm allowed to be a little bit freer and more expressive. Yes. Even changing the room. I mean, I definitely didn't get and I wouldn't have thought you'd be about to, you know, co-create the room in any sense, because I didn't know I would have thought my mind. And I still kind of do think that that kind of needs to be standardised.” (P3)

Further, participants' accounts highlight the value of transparency and communication about the physical environment, including the participant's ability to move and express themselves freely during the session.

To be given that permission is vital for me. During my first dosing, I was frozen on the bed, didn't feel I could roll over, I was worried about wetting myself, embarrassing myself somehow. Thankfully there was a second dose. “Whatever comes up, just do what you have to do”. And I moved so much on that bed, I didn't get up and dance or anything, but foetal I was, not worried about how I might be judged or perceived by people in the room. And the increasing trust helps with that. They really helped me (by saying) "wet yourself, it's fine", and I didn't, but it was like getting rid of another bit of resistance for me. “I can't let go fully, because otherwise, what might happen?” (P2)

### 2.2.11. Familiarisation with the dosing room prior to dosing

The participants' experiences emphasize the critical nature of familiarisation with the dosing environment as an integral part of the preparation for a psychedelic experience. Being introduced to the dosing room is not just a perfunctory walkthrough but a important step in reducing anticipatory anxiety and establishing a setting that feels secure and familiar, akin to a dress rehearsal for the impending dosing session.

“I would absolutely echo the point about doing the prep sessions in the in the dosing room, I think the importance of creating familiarity with the space. Some of the same, there wasn't the sort of journey music but some sort of initial music just again, to create familiarity with the space, see the the headphones, the the eye masks. So I think that all goes towards creating a sense of safety. Because you can have, while you might not might not know what to expect from the actual journey, you have a sort of framework within which it's going to be happening. So I think that was really, really important.” (P1)

A comprehensive orientation to the dosing room serves to acquaint participants with the sensory components they will encounter, from the tactile sensation of weighted blankets to the auditory landscape created by music and guided visualizations. Such introductions are seen as important in mitigating the overwhelming nature of novel stimuli during the sensitive psychedelic state.

“So I think the prep sessions with the therapists in the room were invaluable, I think it's a given I think, there's not much to deny how valuable those are. I quite enjoyed the sense of humour and having a fake plant on top of the IV infusor during the dosing day, because it also tried to blend the very clinical bulky piece of equipment into the rest of the sort of natural theme of the room with beautiful soft furnishings, the wallpaper, natural scenes. It was nice to try to blend the clinical stuff in as well, just to show that while it's a clinical trial, we're still trying to soften the medicalized aspects.” (P1)

Participants also stress the importance of knowing the touch protocol, as unexpected physical sensations can provoke anxiety.

You're being put in a hypersensitive, extremely vulnerable state and you need to trust that you can be that vulnerable. But every detail is amplified. So getting the room right, making sure that the person is introduced to the elements first as well. I wasn't introduced to the weighted blanket that they introduced during my session, and I hated it. (P2)

Furthermore, a detailed explanation of each element in the room, including those with therapeutic intentions, like the weighted blanket, is considered important.

“I was introduced to the room, which was helped really, really helpful like P1 mentioned, being able to have been in that space and to have everything around you. But there were certain elements that I wasn't introduced to, like this really like this weighted blanket. And when that was introduced, kind of I think I was told about it, but I never felt it on me. And when it was put on me during the session, I felt like I was being crushed, and I felt really restricted. I know it's meant to be calming. But I felt really.. yeah, it made me feel really panicky to be honest. So, again, those details are small details like introducing someone to the room, introduce them to the elements within that room as well, as much as you can, you know?”(P2)

## 2.3. Optimising the preparation phase

### 2.3.1. Challenges with antidepressant medication withdrawal

The discontinuation of antidepressants in preparation for participation in psychedelic studies posed significant challenges for participants, with reports highlighting both the psychological and physiological impact of abrupt cessation. In the initial trial, participants recounted experiences of inadequate support and communication, as one participant was abruptly advised to stop antidepressant use close to the dosing date, resulting in adverse effects such as night terrors.

“not that long before I had the dosing they've contacted me for a checkup to go through certain things, and one of them was the they asked me: "have you stopped taking antidepressants?" and I don't remember reading anything about having to stop those... That's maybe my fault, I don't know who to blame for that. But they said "oh, you need to stop taking them. Pretty much now kind of thing. It's supposed to be six weeks before and I think it was four weeks before. And then they said we'll contact your doctor and get you some zopiclone prescribed to help you sleep". And that was unusual. I mean, it's going to be unusual for everybody coming off of medication. And the night before the dosing I had a night terror, which really shook me for the following morning. And I explained what had happened. And you know whether it was a good idea to go ahead, because it was really quite visual, this this terror. I even recorded it on my phone because you know, I wanted them to hear the details in case it was significant in any way. And they said no, just go ahead with it, you know that you should be fine, and everything was fine. But I felt anxious. You know, in case, I just had this sort of feeling that some kind of visual from the night before that sort nightmare thing would come into the session itself. Fortunately, it didn't. And that was unusual. So I think it's definitely worthwhile checking that everybody's, crossed the I's and dotted the T's with the antidepressants. It was a bit bit nerve wracking.” (P4)

Another participant described an experience where the tapering process was communicated but still proved to be too abrupt, leading to withdrawal symptoms that conflated with the effects of the psilocybin, obscuring the ability to distinguish between the two. These experiences suggest a necessity for individualized tapering schedules and more detailed management of withdrawal to mitigate confounding factors in the study's outcomes.

“they did mention it to me, and they did made sure I tapered off my antidepressants. I'd still say that, even in my case, it was too abrupt, I think. So I had quite a lot of withdrawal issues, the sort of hot flushes and the sort of weird kind of waves of heat going through my body and stuff. And that kind of meant that because there were two doses on my first trial, 10 milligramme and 25, milligramme. After the 10 milligramme dose, I wasn't sure if the way I was feeling was because of the withdrawal or the ongoing withdrawal from antidepressants, or whether it was the effects of the psilocybin. I think, honestly, it was a big mix of the two. But I think to have a bit more distance for those withdrawal symptoms to calm down more would have been preferable. I know you then run the risk of taking people off far too soon and leaving them hanging the yeah, there must be there must be a better way I think.” (P2)

In contrast, a more recent study demonstrated improvements in managing the tapering process, with participants reporting that the cessation of antidepressants was well-articulated and managed.

“Like felt like my tapering off of my antidepressants felt fairly well managed. It certainly felt like it was reiterated multiple, multiple times. So I was very, very, it was not a surprise. I knew right from the very outset that I was going to have to come up with antidepressants and that would have to be carefully managed. So it was something that I knew for a while?” (P3)

The varied experiences highlight a important aspect of psychedelic research protocols: the need for carefully managed transition periods off medications.

### 2.3.2. Creating the right setting/protected time

In preparing for a psychedelic experience within a research study, the setting in which participants engage in preparatory sessions is of important importance. Participants discussed the advantages and disadvantages of remote versus in-person preparation, underlining the importance of having protected time to prepare, acknowledging the depth and potential emotional intensity of these sessions.

“But it did mean that these were conversations that were, you know, they were challenging, and I was trying to fit them in amongst work. So I think it's something to be mindful of, to let people know, like, this isn't just a sort of, you know, a casual chat, it could be that you end up talking about quite difficult things, and people should factor that into how they plan their time around it. You know, over time, I learned, actually, let's not schedule a meeting straight in after these chats because they bring stuff up.” (P3)

Participants emphasised the benefit of remote sessions in terms of convenience, especially when trying to balance participation with work and other responsibilities. Remote sessions allowed for flexibility and accessibility, with participants noting that initial screening and preparation could be done without the burden of travel, which could be especially demanding for those coming from a distance.

“Almost all of my meetings were online until we actually went to the centre until quite close to the actual dosing, when I started to having to have some scans and have a final kind of in person session. But the rest of it all online.” (P3)

“I think it was okay. I'd had some experience of doing counselling sessions online, which I'd always been really sceptical about, but I, I had had counselling online and had found that actually, I was able to still develop an element of connection with the counsellor. In my case, I, I trusted the team, a lot. I mean, I really, really gave my trust to them. So for me, I don't think the virtual element did impact. And eventually I did meet them in person. And we did have conversations that took place in person. I couldn't speak for others, though. I mean, it is a factor to to be aware of, and I think my preference would have been, if I'd been able to sort of see people in person, that would have been preferable, but that would have been a lot more tricky to manage, in the context of all my other responsibilities. I work so like, you know, if I had if I had a sort of one hour screening interview, for example and if I'd had to do that in person that would have been much, much harder to fit in, around work, I would have to take more time off. And I certainly did have to take time off for the in person sessions, which meant of travelling to go to the other side of London, spending a significant amount of time at the centre. So it is a balancing act with with other responsibilities in your life.” (P3)

Remote sessions reduced travel and scheduling burden. In-person sessions were still valued as dosing approached because they helped participants meet the team, see the room and build familiarity with the setting.

“Like, we've been used to this kind of online way of working for the last couple of years now. So. But it was, it was great when I got to kind of meet them in person and attend the centre to do the tests and stuff like that. I did a lot of my kind of all my screening took place via online sessions, initial prep was online sessions as well.” (P3)

There was a general consensus that a blended approach might offer the best of both worlds: initial remote sessions to ease logistical demands, followed by in-person visits to solidify trust and comfort with the environment and the research team.

“I definitely think it's a blend should be offered online. I think as you get closer to the dosing day, they become more in person. A lot of my initial calls were on Zoom, initial screening calls with the study site were on the phone, so there wasn't even a face. So I think that the burden of getting to these things, depending on what state you're in as well, could be really hard for people to get in and out of centres, also the sort of distances that might be covered depending on where the trials being held. So I think doing them online, didn't feel detrimental. To me, I think they felt like a necessary first step. But I think absolutely the importance of then, as you go further down the process, doing them in person getting a feel for the room, getting a feel for sort of the interpersonal dynamics in the flesh is absolutely important. So I think both can work hand in hand.” (P1)

“I absolutely agree with P1 know what to say on that. I mean, we'd never had the virtual meetings. So it was quite, you know, quite a task to travel down to London for sort of an hour and a half meeting and travel all the way back to know up north to get afterwards. Virtual meetings would have helped with that, but I agree, you know, the closer it gets towards the event I think there should be more face to face.” (P4)

### 2.3.3. Scales and assessments are burdensome

“once you pass the screening, do you then get bombarded with scale after scale after scale off the scale? And I really think P1, and I have discussed this before, you know about the the burden of that and you almost have to be managing a bit to carry that administrative burden. If you are deep in crisis, I don't think you'd be able to carry that.” (P2)

In the preparation phase of psychedelic research studies, participants often face a barrage of scales and questionnaires designed to measure various psychological states and traits. These evaluative tools, while important for gathering data, can become a burdensome task for participants, particularly those who are already navigating mental health crises.

Participants reflected on the administrative load of these tools, with some feeling overwhelmed by the sheer volume of scales to be completed. This aspect of preparation seemed particularly taxing, potentially exacerbating the difficulties of those in deep emotional distress, as the administrative burden could add another layer of stress to their existing struggles.

“I think, like between the first and second trial, this first trial, it was like they sent me loads of attachments on an email and here's another five, whatever. And it was a bit chaotic. Whereas during the second trial, you were in the room with someone or they sat you down with a tablet that was not functioning very well, but stil, sat you down with a tablet. So you were in the space at the time doing it there. So I think knowing you had that support around you there was someone in the room or just outside the room, I think that was a much better approach. It was still a lot of work, but you didn't then have to try and muster up the energy and the time to do it outside the visits.” (P2)

Comparisons between trials revealed that the method of administering these scales could significantly affect the participant's experience.

The emotional impact of completing these scales was also highlighted. Participants reported that the process could be not just tedious but also emotionally triggering.

“On the sort of the digital scales and evaluators, the thing that was a bit frustrating for me is that it would say like, we anticipate it will take you 40 minutes to complete but it always took me well over an hour, because I just like I would think a lot about the questions. It always takes me way longer than they anticipated it would. One practical thing that was handy was that there was a sort of feature on these online surveys where you could press to save answers halfway through and come back to it later, which for me, on many occasions were essential.” (P3)

“But the other thing I would say about them is not only are they sort of tedious and tiring, but they can also be emotionally stirring as well. I often, I felt quite depressed after them, because, so some of the scales I had to fill include questionnaires that I have been filling out since I was 19 and first diagnosed with my mental health condition. So I've been filling out versions of those, those questionnaires for like 18 years, and realising that you're ticking the same boxes that are like, "yes, I massively struggle with this. Yes, I'm actually struggling, yes, this must have been happening".. it's just quite depressing. It's quite hard. So I'm not in any way saying that we shouldn't have had to fill those out, I fully accept that it's part of the trial. But yeah, don't underestimate that, they're not nothing, it's not just a sort of totally unemotional tick box exercise. Like, there is an emotional resonance to ticking numbers on a scale, which is evaluating how mentally unwell you are like, it's significant. So, you know, I often felt a notice in my mood after sitting for an hour completing these forms.” (P3)

Participants accepted the need for repeated scales and assessments, but recommended that research teams recognise the emotional and administrative burden these can create.

### 2.3.4. Bringing meaningful objects into the dosing room

Advantages highlighted by participants included the ability to co-create the room with familiar touchstones, which served as grounding objects during the dosing sessions. These items often held significant symbolic meaning and became an integral part of the intention setting process.

“I don't know whether you were invited to do this, but I was invited to bring objects with me to my doses, which were kind of grounding objects that I could. So I could also effectively co-create the room. I took some I took a few photographs, I took a couple of objects that were really important to me, I took a blanket.. I sort of added things to the room that could be familiar touchstones I guess at various points. And we spoke about why the objects were meaningful to me so I've had quite a lot of symbolic meaning. So they were just a further tool in intention setting and grounding” (P3)

Moreover, these objects provided comfort and familiarity in an otherwise clinical and unfamiliar environment, helping to navigate moments of intensity during the dosing. The presence of personal objects could serve as a reminder of the world outside the dosing experience, potentially fostering a feeling of safety and emotional support.

However, there were also disadvantages or challenges associated with this practice. For some participants, the suggestion to bring personal items felt forced or contrived, particularly when they were struggling to identify with a clear intention or when in a state of desperation.

“The second trial we were encouraged to take objects for this, but I was in because I was so desperate, and I was like, my intention was to feel less shit rather than having something defined, and I felt like I was really trying too hard. And I took these two stuffed rabbits that I'd had since I was a kid one since the day I was born, and one since I was a few years old. And I don't know, it all felt... It was the whole experience... It just felt really contrived. And it was like, what do these mean to me, they do have some meaning, but I felt like I was trying to force meaning onto them. Yeah, I can see how helpful it could be.. experiences since then have shown me how important it is to have those things that you're genuinely have that connection to your heart and do have that true meaning. But in that situation, everything was like, tried hard and contrived. So I encourage people to have things in the room that they genuinely feel connected to. They suggested a stuffed toy, and I think that's why I went with it. But I didn't really connect with it.” (P2)

One participant shared an innovative approach by bringing in a laser pod to project patterns onto the ceiling, which they found relaxing.

“I asked beforehand it didn't offer you know that I could bring my own things but at the time I had a laser pod which could project laser patterns on the ceiling and this kind of thing. And I took it along mainly for me to relax the night before but then I thought well it'll probably help me relax in the room. So I took it there and they thought it was great. And then after I've had my dosing I donated it to them basically. I said you know if you want to use this, because it relaxed some of the therapists in there to listen to music and watching this thing on the ceiling.” (P4)

“And I think as far as objects go, it's just about.. to me, I think the guidance was either things that are kind of meaningful to you, or in some way relate to your intention, or which could be useful in helping you to feel safe and comforted. So I took a jumper that belongs to my partner, for example, because I knew that that would be a thing I could wear of him that helped me feel safe in the room. But I also took a photograph of myself as a child, which was really helpful, because a lot of my process and working through was around connecting with my inner child, trying to feel some compassion for my younger self. And there were moments when I would, you know, take the blindfold off during the doses, look up, see the photo, and it would really help me sort of touch in with what I was working through during the doses. So for me that the kind of the relationship with objects in the room was, was really, really significant. There was a lot that kind of helped contribute to the journey that I was on. But the key is, it's got to mean something in the first place.” (P3)

### 2.3.5. Leaflets for GP and support system

Participants in psychedelic research have highlighted the significance of more comprehensive inclusion of their general practitioners (GPs) and support networks during the preparation phase of studies. This approach is viewed as essential for several key reasons.

Regarding GPs, participants have pointed out that enhanced communication between the research team and the participants' primary care providers would greatly improve the continuity of care. By relaying information from the trial back to the GP, it ensures that the primary healthcare provider is well-informed about the treatments and progress within the study, facilitating better post-trial care.

“Yeah, that's a really good point. A suggestion maybe as well, to refer information back to the GP? Because it wasn't that long after taking part in the trial, I've been to see my GP for a matter totally unrelated to the depression. And I asked him if he'd heard anything, you know, you've got any information? And he said, no need to know, really very little clue as to, you know, what happened and your results and that kind of thing. And so, you know, maybe sharing some information back from the trial to the GP as well. You know, he was impressed that there was a great result, but he had no information as to really what had happened. Other than, you know, Googling and that kind of thing.” (P4)

“I think it's hard for them to keep current as well, I didn't think you know, they need to know. And I don't know how that is, maybe it's something that gets sent out to GP's, when trials are being organised to say, for any, if you have any patients that meet these criteria, we're doing these trials, so that they can refer because goodness they want to do something. And you know, if there's something that can be done, maybe then if they're a bit more informed of the thing of the opportunities, but yeah, absolutely wasn't through my GP, although my GP has been incredibly supportive and told me when he saw it in the journal published in the journals.” (P1)

When it comes to personal support systems, the feedback from participants indicates that it's important for partners and family members, who play an essential supportive role, to be well-prepared for what the participant may undergo.

“My husband was involved as well, you know, it had some interviews and things, but I think what may be missing, especially for people who are psychedelic naive, is actually almost a, a partner information leaflet, I think, what will be really helpful, which we don't get, you get all the patient information leaflets, but you know, something for the family of the person around, you know, going through the trial. Because I think, especially if those people are psychedelic, naive, they, you know, you come out having had this insane experience, which is life changing. And people don't know how to respond to you, you're your own people, your family. So maybe just some kind... Yeah, I always think it's a bit of a gap that we don't have something to give to the family of the people go through the process. Because as we've heard, how, how much you rely on them in terms of being your support person, and how much space they'll be holding for you that if they have a tool as well, or just some kind of convenient reference. It could help as well with positive outcomes from the trial.” (P1)

Additionally, providing educational materials to family members can manage expectations for the post-trial period, including any emotional or behavioural changes the participant may display.

“I think the video was like, a bit of information for loved ones about what their loved one was about to go through what they might expect to see in the days and weeks afterwards. I think it was kind of guidance around "Look, do not be surprised if you know, your partner, daughter, friend, whoever is particularly emotional, seems a little bit sort of wrapped up in certain things that they are going through, perhaps seems a little disconnected or detached", and some kind of guidance for them on how best to be a supportive person.” (P3)

“I think information well.. like P3 got basically... some kind of, about, you know, what to expect or things to look out for? Yeah, that would have been, you know, I think useful. Because, you know, I was asked a lot of questions, and I didn't really have the answers, you know, from my partner about what things would be like? In terms of the, the sort of the partner, picking the person up after the trial, that wasn't an option. And in a way, it almost sounds selfish, I think I preferred that. Because I think if my partner had been there straight after the session, I would have felt the need to try and explain everything and, you know, give a running commentary on what just happened, rather than reflect on it. You know, and learn the lessons kind of thing. That would just be my take, I mean, obviously, it's, you know, horses for courses, everyone's different, but that seemed to work better for me.” (P4)

### 2.3.6. Involve partner or support system in preparation

In the context of psychedelic research, participants' experiences emphasize the significant role of a partner or support person in the preparation phase. The requirement for a participant to nominate a supportive partner is not just a procedural formality but serves as a critical component of the therapeutic process.

The involvement of a support partner begins with understanding the study's scope and potential outcomes. In some cases, this includes joint sessions with the participant and the research team, where expectations are set, and support roles are clearly defined.

Moreover, these partners provide the research team with valuable observational insights that may not be immediately apparent to the therapists themselves. They can help monitor and relay behavioural changes or shifts in the participant's well-being post-trial, contributing to the overall evaluation of the psychedelic experience's impact.

While some partners, familiar with psychedelic experiences, may find this role within their realm of understanding, others without such background could benefit significantly from preparatory guidance.

“one of the kind of the criteria was that you had to have someone who was willing to be named as a support partner in the whole process, and that they had to be willing to not only be interviewed, but could be on hand during the trial, they would be interviewed during the trial. And that was a crucial element. So he was also interviewed by them. I think we even had a session where it was two of us together to talk about what my support needs might be. So that was a really key element and actually, in my experience was enormously beneficial. So he was totally up to speed with the whole pro assess he was on hand, he picked me up in the dosing days, he and the therapist discussed what support might look like in the aftermath. So that was a key part.” (P3)

“I think he also met with the guides and kind of just had a conversation. Part of his role, I suppose was not only in terms of like, helping to be a support figure. But also, I think his role was also to help them further evaluate the impact of a trial on me. Just so that he could also kind of let them know, or how have you observed any changes in P3's behaviour? What's your assessment of how the experience has impacted? I know that he was asked kind of both questions. But he was certainly he was certainly given prep. I think for him, it wasn't terribly surprising, because he's had experience with psychedelics himself in the past. So he was actually an ideal support partner, because he was not in the slightest bit surprised by anything I went through or how I was afterwards. For those that didn't get that, for their partners or people that were close to them, do you think that that would have helped? How did the people close to you feel about you doing the trial? And what would have been helpful for them?” (P3)

### 2.3.7. Paying for travel expenses

Participants discussed payment and travel reimbursement as matters of access and fairness. While payment was not the main reason for taking part, unpaid participation could exclude people with less flexibility or fewer resources.

From the participant perspectives, trials that did not offer payment or cover expenses limited the demographic diversity of the participants. The concern raised suggests that without such compensation, participation is often restricted to those who have the means to afford the associated costs, inadvertently excluding individuals from lower socioeconomic backgrounds who may not have the resources to participate without financial assistance.

Participants acknowledge the substantial time commitment required to participate in trials, often necessitating taking time off from work or using personal days, which can be burdensome or impossible for some.

“Well, and not just expenses, so getting paid to participate. While I think it's probably the right thing to do, I think when you're looking at a clinical population, the main driver isn't financial at that point, you know, they are absolutely there to try and deal with whatever they are working with. So while I think it's the right thing to do, I mean, the one trial didn't pay, you know, so it's you, therefore, it's the end said, you're pretty desperate, you want something to help.” (P1)

The population of trial participants is still very narrow, as demographically, and that needs to change. By organisations offering to pay for the actual trial itself, you would hope that makes it more affordable for more people to be able to take part. It takes a lot of time to take part in a trial, all have different appointments, etc. If you're on a low income, or you've got family commitments, I think you'd probably be priced out… (P2)

“I had to take time off work to take part in longer sessions, particularly on not only the dosing days, but any long days, we're had to go and have scans and things like that. And I'm fortunate to be able to do that. But I used up my annual leave to do that. I wasn't, I wasn't interested in any financial remuneration, I was absolutely desperate for some, some form of clinical intervention that that would have an impact on my on my health, but certainly raise a really important point about the accessibility, other people may not have a position where they're able to take that time. Yeah.” (P3)

# 3. Dosing day

## 3.1. Navigating the experience

The principle of "trust, let go, be open," often conveyed as a guiding mantra to those undergoing psychedelic experiences, encapsulates competencies that are inherently challenging for TRD patients. This highlights the critical need for an elaborate and patient-centric preparation phase.

### 3.1.1. Trust in guides and team helped to be able to let go of control during the dosing session

Participants linked trust in the guides to being able to let go of control during dosing. This trust was built through repeated contact before the session.

I think it was a reflection that the preparation was done really well, I had developed that trust. And even on the first dosing, it became really intense. I think I had let myself go I, I felt that trust. (P3)

Participants link their ability to immerse deeply into the experience with the level of trust and safety they feel in the treatment setting. Such trust is built incrementally, beginning with the initial contacts during screening and continuing through to the interactions during preparatory sessions.

You have to form a deep bond for the treatments to work properly. So you can let go fully and surrender to it. And allow yourself to be that vulnerable. (P2)

Interestingly, the bond formed is acknowledged as intense and substantial, which leads to a paradoxical situation post-trial where the participant must grapple with the potential loss of this deep connection once the study concludes. The transition from being deeply connected to possibly having no further contact highlights the unique dynamics of therapeutic relationships within psychedelic research.

“And I, you know, I partly attribute the value I got from the dosing, eventually, to how deep I was able to go and I linked that to the trust I felt and the safety I felt in the room which I linked to the guides and the the trust that I did build in them through the conversations which was started all those months earlier without but was something about the way they spoke to me, their compassion, their sensitivity, and it starts with those contacts. It starts even when you're just going through some boring paperwork, like everything about the trial.” (P3)

In essence, the degree of trust in the guides is a important part of the participant's experience, deeply influencing their ability to engage with the psychedelic experience fully and the overall therapeutic outcome.

### 3.1.2. Difficulties with letting go of control

In the context of psychedelic experiences, the ability to let go of control is often important for a participant's therapeutic journey. A participant describes being frozen on the bed during their first dose, paralyzed by the fear of losing control and facing potential embarrassment in front of the guides.

The importance of addressing this fear is highlighted in the participant's subsequent experience. After being assured by the guides that it was acceptable to respond naturally to the body's needs — even to the point of wetting the bed — the participant was able to move freely and let go of the fear of judgement.

To be given that permission is vital for me. During my first dosing, I was frozen on the bed, didn't feel I could roll over, I was worried about wetting myself, embarrassing myself somehow. Thankfully there was a second dose. “Whatever comes up, just do what you have to do”. And I moved so much on that bed, I didn't get up and dance or anything, but foetal I was, not worried about how I might be judged or perceived by people in the room. And the increasing trust helps with that. They really helped me (by saying) "wet yourself, it's fine", and I didn't, but it was like getting rid of another bit of resistance for me. “I can't let go fully, because otherwise, what might happen?” (P2)

For this participant, increased trust helped them move through resistance and engage more fully with the experience.

### 3.1.3. Moving through difficult emotions

Navigating through difficult emotions and facing fears head-on can be an essential aspect of the psychedelic experience. Participants in psychedelic research studies have highlighted the value of moving through uncomfortable experiences rather than avoiding them.

“I remember on my last dosing, I was really struggling with the idea of it being the end. I was really struggling, I didn't want it to come to an end. I was very anxious about about ending. And that became a real theme during the actual dosing, particularly for the first portion of it was very anxiety driven. I mean, I became quite terrified, I couldn't let go, very confused about what's happening. And at one point, took my blindfold off, and I just turned to them: I can't do this, I don't know what to do, I was so overwhelmed. And I can't remember the exact words, but one of them said something along the lines of what would it be like to just go through it, face it, ie, rather than trying to turn away from the anxiety just literally like to hurl yourself at it. And that's what I did, I put the blindfold on, and it was a bit like running into a storm. And it was extraordinary, because I then had this experience of like riding right through it and obviously coming out the other side, which is what you'd expect with the trajectory of psychedelics, but it was such a useful piece of advice.” (P3)

The encouragement from guides to face fears rather than retreat from them is likened to committing to the full ride of a roller coaster – once it begins, you are committed to the experience until the end. This metaphor serves to prepare participants for the full spectrum of sensations and emotions that may arise during a session, encouraging them to lean into the experience rather than resisting it, which can lead to a negative or challenging experience.

One of my guides compared the experience to a roller coaster and was saying that people don’t get on a roller coaster and then start panicking halfway up and ask for the roller coaster to be shut down. You’ve just got a flow, you just go with the experience. And you’ll probably have a negative experience if you try to stop things. I thought that was a really good analogy and got me used to the idea that I just have to go with what comes up. (P4)

Support from the research team can be important when participants encounter difficult emotions. For example, one participant was surprised by a sudden outpouring of emotion during the session, leading to tears.

“Cry was remember, I mean, I didn't expect it, it just seemed to come out of nowhere. There was a part of it, which was really weird encounter. And I felt like they didn't know how to deal with us. There was, you know, the kind of backing off a bit and when they were talking to me, physically, that was sort of, you know, you know, we do a kind of thing and just kind of try and calm yourself down, calm down, and it's okay to cry and then which was helpful. But I felt like there would have been an experience in that respect. Maybe because I have not been there before, I don't know.” (P4)

“I think even been a few weeks after you benefit not only the light, but, you know, having I think, yeah, when I bawled my eyes out. Yeah, they just offered me a tissue, and we just kind of present that and they weren't trying to stop me. They didn't look scared. Of what do we do this? I was probably just a few more people along than you. They probably talked about it.” (P2)

Participants felt that study teams should be prepared for a wide range of emotional responses and should support participants to move through difficult material when it arose.

### 3.1.4. Using humour to cope with darkness

Navigating the intense and sometimes ominous landscapes of a psychedelic experience can be a formidable endeavor. For one participant, a sudden shift in perspective—courtesy of humor—became a important tool in coping with the emerging darkness during their dosing session.

The distortion of time during such experiences often makes them even more disconcerting, leaving the person uncertain if moments were stretching into hours. In anticipation of a follow-up conversation with their guide, the participant was curious to uncover the objective duration of this daunting episode.

Interestingly, it was the introduction of humor that dispelled the ominous air. Upon expressing fears of perpetual insanity and longing for normalcy, the guide's timely jest—"Well, you know, when you find somebody who's normal, then just let me know"—pierced through the solemnity of the moment.

“But during the second trial, I was like, my, my guided turned into the devil, and it was just horrible, it was just really like dark. And I was scared for my wellbeing and scared, I was going to hurt myself or hurt someone else, or whatever. And I don't know how long this period lasted, because you have no concept of time often. I'm going to be seeing my main guy tomorrow, actually, and I really want to ask him that question: "how long did that last?". Because he might say, five minutes, he might say three hours. But he just introduced some humour. He was like, right, what are you scared of? And I said, scared of going mad or never being normal again. And he had been taking me really seriously up to that point. And that kind of, sort of maintained the gravity of the situation. And at that point, he just said, Well, you know, when you find somebody who's normal, then just just let me know. And it was just like, it's not the best joke in the world, but it just lightened things. It was just like, ah humour. And after that, that kind of transformed everything.” (P2)

Humour was discussed as one way facilitators could reduce fear and help participants stay connected during difficult moments, when used sensitively.

### 3.1.5. The use of touch

Touch was discussed as a substantial part of some participants’ dosing experiences. Participants differed in their needs and preferences, but agreed that touch required clear consent and careful boundaries.

For one participant, physical touch—or rather, the lack of it—surfaced as a gap in the clinical trials. They contrasted this with a retreat experience where touch was more freely given, highlighting the value of physical comfort in navigating the psychedelic journey.

I think touch is incredibly important and I wasn't offered enough touch during the studies. I'm so pleased to hear that the anorexia study was different. While I can understand why there's less touch potentially in clinical trials, I genuinely think it's missing. (P1)

I also think the touch element of psychedelics is important. Coming back to the birth analogy, so much of this pain is preverbal and in some ways, it is about reparenting and holding. Touch is so powerful. (P1)

Another participant echoed the sentiment, advocating for touch as an essential component due to its cognitive and physical processing facets. They recounted how comforting gestures from the guides, like a hand on the back or shoulder, played a symbolic role in navigating through themes of re-parenting and inner child work.

I just think touch is essential because we process things cognitively, but we process things physically as well. And I don't think there's enough understanding of the symbolic impact of what being in our bodies means, how we carry our trauma within our bodies, how the body might be aware of symbolising. A lot of my psychedelic experiences were related to themes of parenting, inner-child work, mothering, all of that stuff. So, moments where the sitters were comforting me by having a hand on my back, on my shoulder, was part of this symbolic process of working through that. (P3)

Addressing the safeguarding aspect, a participant highlighted the delicate balance required when navigating touch in a therapeutic setting.

“I do think it's important to, to, you know, have these safeguards in place. And if that means we can have more touch, all the better. Because I do think the touch I was explained was very much hand holding, my guide and I even spoke about it, because I did get a hug. And after it's all you know, she's very much said we don't hug you know, but I really needed a hug after that DMT journey, and she felt it. And she read the room, right, because she is very attuned and very sensitive. But yeah, if she's any other therapist who maybe didn't know me from a previous trial, or maybe she'd feel unsafe to do that, even though that's what she knows I need at that time. So you need to safeguard the therapists, right?” (P1)

The participants also discussed the complexity of touch, particularly for those who struggle with physical contact due to past trauma.

I really struggle with touch and living in my head rather than in my body because of what my body's been through. That's been my refuge, I've been so disconnected. So for me it’s important to agree beforehand the parameters of what that touch may entail, to have a conversation with someone: “what are the boundaries?” (P2)

“And there's another layer to it, which again, some people may feel super uncomfortable with, but there was a moment during one of my doses where I don't want to go into details, but I had my blindfold slightly removed. And I was looking directly at one of my sitters, and I thought she was someone else, you know, it was in the depth really depth of my journey and and she represented another figure to me. And I reached out and I stroked her hair. Because it was just of what I was processing. And she didn't flinch. And she allowed me to do that. And that's crossing a certain boundary, to a certain extent, but it was really important to me because I had a very intense, feeling compassion towards her, she was no longer that person, she was someone completely different. And, again, that was a really tender and important moment for me. And it didn't last long, I didn't do anything other than just reach out and stroke her hair. But, yeah, all of these things are incredibly important. And I believe that I was filmed, but I think it's a testament to how discreet it was. I barely even remember the camera or anything about it. I do remember them saying we are filming this, and we're recording them. It was certainly so unobtrusive that it didn't interfere.” (P3)

“So it was incredibly intense, I think I went quite deep and I think that says a lot about the trust I felt in my sitters and about the into the preparation and intention setting you've done beforehand. and they were quite active in terms of how they physically comforted me, which was really important. At one point, I was curled over on the bed, and they were either side of me just with their hands on my back. And I honestly, I don't think I could have felt safe to go that deep and to really face the trauma that I was I was facing if I hadn't had them physically helping me to do that. And they were so again, I just, I think they're remarkable, they were so sensitive about when it was right to step back and let me be on my own with it, and when to come in and be physically containing and present literally hand on me, helping me to physically handle what I was experiencing. And then went to take a seat and let me just, you know, curl up on my own. So yeah, just just I'm aware, sometimes when we're talking, we're talking about kind of going into these very, very profound, very visual, you know, your typical psychedelic experience, but it's certainly the case that people can, can go very deep and have very, very intense experiences on on low doses, too.” (P3)

It's not just the moment of the touch, but when the touch is removed.. I have this memory of this intense experience of being comforted in the depths of my pain, and how much I needed that, I needed their companionship whilst I was going through that. But at a certain point they removed it, where I understood symbolically, I had to retreat back into myself, and that part of my journey was understanding that ultimately, I had to carry it on my own. So there's something very important about what the touch represents. (P3)

“Yeah, actually, that will be everyone's experience was a bit more than potentially other people might be on that experience. But yeah, even more coming out the experience really wanting to, coming back to the touch thing. I really needed a hug. But not only that, it was part of it. But I got and I really needed a really needed that hug. So yeah, just reaching those agreements and what more can be done touched on to cry. Yeah.” (P1)

### 3.1.6. Staying with the process and ‘going back in’

Participants described the end of the acute drug effects as a phase that could still contain insight and meaning. They valued encouragement to stay with the process rather than move too quickly into analysis.

One participant recounts the advice of a seasoned figure in psychedelic therapy who urged them to re-engage with the experience, despite feeling that they had returned to a normal state of consciousness. This guidance was important; the individual might have otherwise defaulted to an analytical mode, potentially curtailing the depth of the experience.

He looked at me and he said: "You should go back in. The medicine is still coursing through you." I'm feeling perfectly in the room and agree, "Well, okay, maybe I do that" and I put the eye-mask back on. I could have continued speaking and started my usual process of controlling and analysing, boxing it up. But he was absolutely right, there were further deepenings and epiphanies to be had. (P1)

Continuing to use the eye-mask and headphones helped some participants remain inwardly focused as the session was ending.

“I mean, I'm a big believer in, while there's strength to be had from the objects in the room, the lights on the ceiling, I'm a big believer in actually just keep going and keep going. And I really, really believe in the importance of the eye-mask and the earphones, that inward focussed attention, and then the space and the objects, you know, very much helpful for the integration. So I found that comment and being invited in really, really powerful.” (P1)

Participants also reflected on the dosing room itself. They described the room and its objects as part of the experience, rather than as neutral background.

“I want to say about the interaction with the space is that, like, it's absolutely fundamental in helping the person to, you know, be in a kind of the right headspace. But I think also, it's important to be mindful, how much is the person taking the blindfold off and coming back into the space, because that might be a way of disconnecting from their journey, if it's getting a bit overwhelming. My guides, I found were very helpful in, there were quite a few moments, I would lift the blindfold off, and I would have a moment of connecting with the room, I would even speak to them a little bit. But I think they were quite, you know, they would then very gently, perhaps nudge me towards maybe putting the blindfold back going back in. Because I think sometimes, you know, it's a different experience. If you take the blindfold off and your eyes open and you're back in the room, it's a different experience to then being fully under the blindfold fully amassed. And I think is important to just be aware and sensitive to how much the person is bringing themselves back in the room to avoide or is it helpful for them to maybe go back in under the blindfold?” (P3)

The discussion extends to the implications for guide training.

“hat's interesting from a training point of view, P3, because again, I think very much with the sort of depression trials, it's very much about put the eye mask on and go in go in go in and through, even through it's very much I certainly was given permission to open up and use the space, but I distinctly remember taking it off and trying to intellectualise and start the sense maker. But the message was, go back and go back and use the medicine while you still have it. Trying to make sense of it. So I think that's to a large extent, why so many people are lying down because it's hard to dance around with your, your eyes blindfolded, I guess.” (P1)

### 3.1.7. Freedom of expression during dosing day

Freedom of expression during dosing was discussed as important. Participants wanted to know in advance whether movement, sound, emotional expression or requests for support were acceptable.

One participant recounts the explicit permission given by the facilitators to move and behave as they felt inclined within the confines of the session room. Such autonomy, they found, was important in alleviating their day-to-day preoccupations about societal expectations and behavior.

“I think also, I guess, something that I think was useful for me was being told that I could, within reason, behave any way I wanted to within that room. So, in my day to day life, I tend to worry quite a lot, you know, what's expected of me in this situation? How am I expected to occupy this space, should I just be sitting quietly in a corner? It's something that I'm hyper aware of. So when they introduced me to the room, and they sort of showed me that there was a sort of, you know, clinical bed that could be adjusted, and we worked out what felt like a good sort of angle, but they also explained, you can get off it, if you want, you can move, and that's exactly your call to do that. And that was really helpful for me.” (P3)

This participant's experience stands in contrast to the commonly depicted portrayal of individuals lying motionless on the bed during psychedelic sessions. It raises the question of whether participants are adequately informed of their freedom to move, suggesting that the therapeutic benefits may be enhanced by actively communicating the availability of this option.

“And actually, I did, I moved a lot during my doses, I actually danced at one point, actually, on every dose, and I danced quite a bit. That was very healing for me to actually, to have that sense of liberation in my body. I didn't watch these before the dosing, but since I've done a trial, I've watched all sorts of documentaries that exist out there about about psychedelic trials. And I'm fascinated to see people just lying. So still in the bed, and that's totally fine, if that's if that's how people have experienced their journey. But I also wonder, where they told that they can move around if they want to, because I found that, you know, the whole thing felt very, very physical for me. So I do think, you know, it's helpful to give people a sense of empowerment with them that this is your space, you know, to use as, as you feel called to, obviously, within safety limits.” (P3)

Participants described how explicit permission to move, express emotion and not worry about being judged helped them engage more fully with the session.

I was scared to roll over, I felt frozen. I felt like I couldn't turn around, because I was worried about being rude to the people on whichever side of the bed. It was really a symbol of how I navigate life. Worry about upsetting people, worrying about saying the wrong thing, all of this constant anxiety… My general trajectory with all of my psilocybin experiences has been resistance, terror, fear… Resistance really is what I now see my depression as, it’s resistance to thoughts, feelings, emotions, and the more I push, the tighter I am within that grip, the more brittle and restrictive it is. (P2)

Once I got through the dark phase, I'd looked my dad in the eye, faced down my demons and I got to a stage where I was like, "I can deal with anything". It was blissfully happy. It was like: “I can deal with sadness, I can deal with pain, I can deal with all of these different emotions, and approach them with openness”, which was the opposite of depression, where you're constantly pushing back against so many different feelings and emotions that you have. And that carried on to afterwards, for several months, that openness. The seed for that was sown with the permission to do whatever I needed to do. (P2)

It stays with me to this day, and it’s only such a simple phrase. I was a bit concerned about: “am I doing the right thing, I’m not set up in the right position…?” And one of the guides said: “it’s your day” and that resonated so much with me. It’s time for you to explore, it’s all about you. Previous to that, I’ve been trying to keep so many people happy in situations, I’m not really thinking about myself. And when they said that: “it’s your day”, I thought: “yeah, it is. It is my day, I can relax”. (P4)

### 3.1.8. Worrying about the guides or therapists

In the context of participating in psychedelic research, individuals with treatment-resistant depression (TRD) often carry the weight of high expectations, not only towards the therapeutic outcomes but also in their roles as study participants. As reported by several participants, there is a pervasive concern about meeting the perceived demands of the scientific process and not wanting to fall short of the expectations set by the research team.

“Because I think you do, you'll end up worrying, you worry about, you know, the science of it, you worry that you don't do the right thing, or don't do enough for.. or opt out of things that are really important, or, you know, so, you know, given that reassurance is important.”(P1)

One participant articulated a deep-seated fear of failing to contribute meaningfully to the science or "spoiling" the trial, reflecting a broader anxiety that they have contended with throughout life. This is compounded by the gravity and novelty of the experience, wherein participants feel they are part of something significant and groundbreaking.

“the gravitas of it.. you know, going through you, I mean really builds up, you feel you're contributing to something. So if you're opting out of something, it feels, you know, it can make you feel quite bad if you don't get that reassurance.” (P1)

Reassurance, as such, becomes an essential component of the therapeutic framework, offering participants the affirmation that there is no singular 'correct' way to navigate the experience.

The reassurance is key. I was terrified that I was going to somehow spoil it all. It felt like such an incredible opportunity. And I was paranoid that I was going to somehow not do enough to make the most of the opportunity. That's a very familiar anxiety that I carried around with me my whole life. But I did need some reassurance from people that there isn't a right or wrong way of doing it. You're encouraged to engage with the whole process. (P2)

Furthermore, a sense of responsibility can become overwhelming, as exemplified by another participant's recount of feeling as if they had let the team down during the one trial.

I really felt like I let everyone down on one of the trials, I felt like there was so much expectation. although I think I imposed that expectation. And I was like, "this has to go right", and obviously, I've benefited the first time, so I was desperate for that lifting of the depression and anxiety again. (P2)

“the reframing afterwards was vitally important to me, for the rest of my life really, being here now. If that experience hadn't been reframed, I've just written it off and gone "This doesn't work at worked once, I was lucky, but never again".” (P2)

## 3.2. Influence of the setting

### 3.2.1. Sensitive and aware staff

This theme was discussed at length and the participants expressed that staff attitudes and behaviours had a major influence on their experiences.

Participants emphasised that all staff in the room contributed to the setting, not only the main facilitators. Staff conduct, tone and continuity were therefore seen as relevant to participant safety.

A participant highlighted the necessity for staff to approach each interaction with the utmost care, likening the environment to a neonatal ward where sensitivity is important. The process transcends mere medicine administration; it is a choreographed encounter where every element—spatial dynamics, gestures, and dialogueue—carries weight.

“And yeah, I would just say that because, yeah, that that sensitivity, I think which we weave throughout the entire patient journey about that this isn't just a normal medicine administration. There is something about the space and the dialogueue and the intensity that maybe the staff are feeling, you know, like coming into the space in a sort of gentle way. Is can't be over. and emphasise that every step of the process kind of feels like, like a neonatal ward. In some respects, you need to really be dealing with everything with kid gloves. You don't want to freak the baby out. You know. So it's that sensitivity because it isn't just the "wheel, the trolley in, plug it in, administer some meds". So I'd say that was probably my, my biggest thing about pre dosing prep and dosing.” (P1)

“And I suppose that the sort of key thing about the experience, the dosing is just, I guess, I'd really encourage you to think about, like the choreography of it, the drama of it, in terms of like, every gesture, every aspect has meaning. So it's like just being I mean, personally, I think that in trauma informed trauma informed therapy, therapists should have that level of awareness anyway, about the meaning of every aspect of the room and how you behave with the patient. But generally speaking, in therapy, people aren't necessarily thinking about, you know, where should I put the chair? Or, you know, at what point should I close the door? Or, if I'm giving them a prescription, how should I do that. But in this setting, you need to be attentive to all of that, because it all has symbolic meaning, particularly once the individual has then taken the dose. So I found it very interesting, for example, that the psilocybin capsules were given to me in a in a really beautiful little carved wooden bowl. So again, it's like, this is not popping a pill, you're not opening a little kinds of boxes or bottles that you'd get from pharmacy, this was, you know, felt like there was a nod to spirituality.” (P3)

Discussions also surfaced regarding the importance of pre-dosing introductions to the setting's various elements, suggesting that a well-informed participant is more likely to have a constructive experience. This preparation enables participants to have clarity about what to expect and alleviates anxieties related to navigating the psychedelic state.

“I think just ensuring people are introduced to it beforehand, and the individual elements that will come into play during dosing, I think that's really, really important. Yeah. I think a lot of what we've spoken about with how the guides behave or whatever, I think it's just rather than, as P3 mentioned, sort of being really anxious about getting it wrong, or whatever, I think it's just, it just really speaks to the fact that if you get the right people, you know, with the right kind of level of sensitivity and the right experience of how to deal with people in sort of hyper sensitive and hyper vulnerable states, I think that's really the key with all of it.” (P2)

“they are the signs outside exam rooms "keep quiet, you know, exam in progress kind of thing" I think those are pretty much essential because I think there was about twice maybe there was some shouting going on outside. And it turns out that they were actually shouting at the sitters that were with me because they've left a mess in one of the rooms or something. And one of the nurses was shouting at them. So you know, a keep quiet sign or something like that, or words to the stuff beforehand, kind of thing that, you know, be quiet when he got past this room, because it was a bit alarming to hear people shouting and what have you.” (P4)

“I spent a lot of the majority of dose with my blindfold on but there were moments where I sort of lifted it up and occasionally if I lifted it up, and I remember lifting it up and seeing sitters like doing something else and like, like I completely understand, like, there's a long time to just sit there with someone who is not really doing much. But at times I found that a bit jarring. I was like it's slightly like it's really difficult to put this into words but you know when you have that sort of feeling where you're like, "oh my god, what is this? Like? What is like I don't understand what this is like, Hold on Why are people put writing notes?" like this weird, this disconnect between what's going on in your mind and then this sudden, jarring facing with reality. And someone I think at one point she was on her phone and like, I was like, "oh my god", and luckily, they were amazing because they immediately saw I had my blindfold off, and they would come to me and they would soothe me. And I mean, I really don't have any complaints about my sitters at all. I think they were extraordinary. But I suppose it's just being aware of your behaviour in that setting. And just being super mindful of like just not disrupting the person anyway at all. I imagine making this job super challenging. But nevertheless, even something really tiny does have the potential to, you know, really quite significantly disrupt or confuse or alarm the person who's going through the journey.” (P3)

“I think mentioning that as part of the preparation for what the sitters will be doing during the journey is actually an important point as part of the facilitator training. Because I've got mixed feelings about this, I'm so happy you prompted this because I hadn't thought about it. It's kind of like when you're giving birth and you have a doula the best doulas won't be nervously looking at you all the time. Like I felt a bit like I'm, when my sitters when I took my mask off, and they're looking at me to see if I need anything, I felt like I was a specimen in a petri dish. And I know it's science. And I thought, yeah, but I just need a bit more cocooning. I don't like them to look at me so much, right? So when I'm reflecting on the birth process, which to me, it's not too dissimilar process to this, you know, you almost need somebody who's I don't think they should be on their phones. Absolutely not. But I think somebody's just reading a book, and when they see you, they come away from the book and say they'll be just doing something gentle, knitting. To me, it's like almost like, because that almost gives me confidence. I think in the birth process, again, the medicalization of birth, you know, everyone's on top of you. And what you need is somebody who's just holding space and doing something gentle and nourishing. And allows the process to unfold because that's what's happening both with birth and with psychedelics, right, there's a process unfolding. And if you need something, they'll step in, you know, but actually, just creating that space has an intelligence. And you're doing your thing, and this is unfolding, as it should, as opposed to sort of urgency that if you just take your mask off this, people were looking as if something was wrong. And I'm like, "is something wrong? No, I'm, I'm fine." So I'm just yeah, just allowing that in terms of the training, it is such a subtlety of like, this is a process of unfolding and instilling that confidence in people.” (P1)

“I also want to pick on the sort of disruptions, My bed was quite close to the wall. So the sitter, there was I, my two sisters on the one side, and in the DMT trial, and as such on either side in the one trial, and the bed was quite close to the wall. So every time a sitter got up to go to the toilet, have a cup of tea move, just move, which they can do, because it's a long process, but every time they moved they bumped against the bed. And I actually found that that yeah, quite quite disruptive, because then I'm worrying where people go, "Is it lunchtime? Do I need to be going somewhere? Should I be eating?" you know? Just because somebody bumped the bed and you all of a sudden spin out because there's movement and you're not moving? So just space around the bed so that you don't bump it. And the room itself.. I knew everybody in the room that was with me for the journey. Obviously my two sisters, but with the the DMT trial, I also had a lot of clinical, like the medical team in the room with me.” (P1)

“I had a really magnificent nurse who was helping with the first dosing and prep and everything. And she was really magnificent if I could track her down, I absolutely wanted to say thanks again. I mean, I did at the time, but I don't think she realised the impact she had because again, she wasn't there as a sitter. And when they told me she was not going to be there for the second dosing, I think I looked visibly a bit spun out like why won't you be there? And she actually was an annual leave, she actually changed. And it was just for no particular reason, it was just rotas. She actually changed her shifts. And I think maybe that's why I feel so indebted to her. She actually went over and above just to make sure she was the same person in the room with me on dosing day. And actually, that should be a standard I think even the clinical team while you're so focused on having the same sitters, I think we underestimate the impact of the clinical team in the room.” (P1)

“We had another chap can't remember his name at the moment who sort of oversaw the team and he was in the room and felt really comfortable with him because I knew him and you know, I think it's really important to know that the the clinical team in the room as well if they it's more than just the sitter's and, and yeah, again just emphasising what people are going through because even on the DMT experience i i had a very visceral experience and I was really on comfortable in my body but acutely aware I had all these IV lines. So when I tried to move, I can move now and I moved my arm across me just to try hold myself because I was like, I don't know it's crumbling a bit. And, and this lovely, incredible nurse straightened my arm out, because she's obviously drawing blood. So I'm not sure what she's doing. But it felt like quite a quick abrupt movement, which felt me made me feel like I did wrong, I shouldn't have done that, and what's going on, and you know, but I can't not move my arm, because I'm really uncomfortable. And so then I like, bent my head in and then afterwards Graham's, like, when you bend to head in, do you remember that? Yes, I remember that. And I knew because I knew that we're no IV lines on my head. So that was my body that could like sort of move. And I didn't wanna move my legs because I thought that would get too messy with blankets and lines and things. So just a real awareness of what my body was in at that time. And while it was okay, I don't think it was a bad experience, if my arm had just been straightened out a bit more gently, because I was, yeah, I guess you assume the person's not in the room, like, cognitively. But I was and you know if it had hurt, I wouldn't have been able to say, because I was not in the room verbally. I wouldn't be able to talk at that point. But I like I had awareness. So I think always assume, especially with psychedelics, that the person has awareness, because you just don't know.” (P1)

“Just for the sitters as well, be careful with some of your responses. I was having all these crazy, deep, deep thoughts about, you know, the universe, atoms and this kind of thing. And I said to one of the sitters, you know, I still can't get my head around what goes on inside atoms, because I knew he was, you know, scientist kind of guy, and we're having a little chat about that, and then he said, "but of course, you know, we might be living inside an atom now" and then that just absolutely fried my brain completely to the point where I couldn't physically speak. And he said "it's okay, put the blindfold back on".” (P4)

“Something about external interruptions. So like I obviously had the fire alarms going off, but they also had noises from outside the dosing room. In DMT trial there were signs, even though there was a double door to the treatment room at the study site, so it was really quite loud bangs keeping the rest of the unit quiet. Not even fire alarms, something about other world - intrusions. Were going into the suggestion shoe. Tick what's the best one?” (P1)

### 3.2.2. Influence of the setting

Participants described the physical setting as important because small sensory and procedural details could become more salient during dosing.

Participants expressed that the decoration and transformation of the dosing room held significant importance. One described the transformation of a familiar clinical room into a serene, forest-themed space complete with spiritual objects and personalised grounding elements.

“And then once I kind of gone through that part, then I was taken into the room, which had obviously been completely transformed, and perhaps at the dosing, and I've been in that room. Previously, I've been in it for the other prep sessions. I've been in it when I had my EEG. So it was quite sort of striking to see the whole place transformed. And they really went to great lengths to you know, just make it like this amazing space, it was beautiful. Actually, they've done a lot to the place, they have this incredible screen, which I guess they had printed specifically for the trial, which was, you imagine, like a kind of standing folding screen. But it was a beautiful photograph of like a forest glade, that had been printed over the entire screen, which was remarkable I mean, that was one of the most powerful aspects from actually looking at it when I took my blindfold off during the dosing. So there's also like salt lamps, and, you know, lighting been dimmed. Lots of interesting sort of mystical spiritual objects. And obviously, I've been encouraged to bring my own grounding objects, which I could then put around the room as well.” (P3)

“And before I actually took the psilocybin, we again, went through some breathing exercises, they read me a poem, we did a bit of kind of guided visualisation, just a little bit to help get me into that sort of mentality. And the playlist was already playing, so the room was already filled with music.” (P3)

You're being put in a hypersensitive, extremely vulnerable state and you need to trust that you can be that vulnerable. But every detail is amplified. So getting the room right, making sure that the person is introduced to the elements first as well. I wasn't introduced to the weighted blanket that they introduced during my session, and I hated it. (P2)

“I think one thing with the preparation, but it also goes throughout the whole dose was that my secondary sitter on the second trial had a cold. So on a on a really sort of, yeah, on a positive front, she still turned up, she was still there, she was still there to sit for me the day wasn't cancelled, it wasn't, it all still went ahead. But the fact that there was somebody sitting next to the bed sniffing every 30 seconds or so. And I knew she was ill. So I was worried about her welfare as well. And just like, it really kind of affected my ability to let go. And I also managed to get her cold as well. So that period afterwards, and then I'm jumping ahead now but that period afterwards where I was hoping things would seem more alive and I'd be feeling more I'd it was like the cork was in the bottle because you know, the numbing of the cold. So yeah, it's finding ways around that it's very difficult. I mean, you don't want to be kind of letting people down. But then in that situation, maybe a delay would have been preferable. You know? Yeah.” (P2)

However, the participants also highlighted the challenges of navigating an environment where every detail is heightened. An unexpected element, such as the weight of a blanket, could evolve from a benign detail into a source of significant discomfort, highlighting the importance of familiarizing participants with all elements of the setting beforehand.

The journey to and from the restroom also emerged as a noteworthy aspect of the psychedelic experience.

“And it was interesting as well, I think coming back from trips to the loo, it would take me a minute to like, yeah, it just it obviously disrupts well, actually disruptoing is an unfair word, because it's all part of experience. In some ways. There were moments with my loo trips that felt like adventures, and then I'd come back to the room and I'd be like, "Oh, wow, and now I'm back in it again", like it all became part of the journey. But I guess what I'm trying to say it's yeah, just you'll need this kind of level of attention to details, I guess.” (P4)

“And then I suppose the other thing I kind of want to say about this is you may also need to be really mindful about how to support the individual, like if they need to go to the loo. That was a really big thing for me. So obviously, you have a level of control over over the room itself. So they've completely transformed this room. But this was a room in a clinical research unit. So outside of that room, there were people walking around with kind of nursing uniforms on, it wasn't a hospital, but it had a very clinical look to it. And I had to use the bathroom, at least twice, I think, during the course of my dosing, which meant they had to escort me out of that room down the corridor to a bathroom. And, you know, obviously I was still very heavily under the influence of psychedelic. And that entire experience was quite interesting, quite overwhelming in some ways. I have lots of experience of being an inpatient in hospital, both psychiatric hospital and medical hospitals. So certainly, I think on my first dosing, when I first had to go to the loo, I had a real horror moment where for a minute, I thought I was back in hospital in psychiatric hospital, I thought I've been admitted. So I was a bit freaked out. And they had to kind of just really like, almost like form a little... I don't know, I can't explain it but remember that both therapists like, but like on either side of me just protecting me and shielding me, they encouraged me to kind of keep my blindfold on. So I was sort of trying to like shuffle down this corridor. And obviously, key thing as well, they had a special piece of like cardboard that they, they went into the bathroom before me to block the mirror, which is like essential. So again, just these all these protective measures that they were trying to put in place to help me not feel too disturbed by, you know, seemingly, you wouldn't normally think anything of it, a patient going to the loo and individual going smooth during during that consultation. But this is a big, big deal as a part of the experience.” (P3)

“I had to take that horrible sterile walk into this clinical brightly lit white. Yeah, yeah, it was it was tricky.” (P2)

“Yeah, I had to leave the room go down the corridor to the end and turn. The thing that was jarring for me, it was obviously they, you know, you create the dosing room to be incredible, perfectly set up for the setting, but they can't transform the entire unit. And the thing that was certainly on the first time I think I started it was quite jarring because it reminded me being in hospital. And I've been in hospital both my mental health but also physical health. So that was a slight I have moment of like a slight horror because it was all like it was just a bit disorientated. So there's just about practicalities, obviously covering mirrors.” (P3)

“I think also bearing in mind that a medical context can be can be resonant and triggering for people for all kinds of other reasons. So I've, I've also had cancer. So for me, having MRI scans.. I know I mentioned earlier that I actually quite enjoy being inside them, but they were also very, was very, very resonant of when I had cancer, which was about five or six years ago, and there was a lot about some of the more medical procedures involved in the trial.. it was just, it was fine, it was manageable, I'm used to having experiences that trigger me, it's it's not an uncommon thing, but again, it's just something to be mindful of, you know, people may have a whole host of different encounters with very medical settings and being tested, being scanned. Like P4 said, we have other associations with the experience of being in something like an MRI scanner, which is very, it's very unusual that we are in a scanner, having things mapped for us sort of, you know, in that kind of inquiry, in the interests of some kind of trial, a normal association is that they are looking for something that might be wrong. Even just the associations with a hosptial.. I've been an inpatient in psychiatric hospitals as well. So on the dosing themselves, when you're a lot more open and susceptible sort of surroundings, the moments were I had to go to the loo, for example, I was a bit freaked out because you come out of the protection of your little cocoon, and you're sort of walking down this corridor, and there was a lot that was bringing back memories of times, I've been an impatient. So again, it's just understanding the implications of the environment.” (P3)

“I just had a penny dropped moment for me because I have a lot of sort of physical trauma in my family and extensive hospitalizations. My dad was in ICU for 18 months. And I remember the first time going in and my mom had brain cancer. So it so packed up with lots of clinical experiences.” (P1)

My concern was that something might go medically wrong. But when I got there, I realised that the place looked like a hospital and I thought: “well, if anything does go wrong, I’m in the right place” and that was a relief. (P4)

### 3.2.3. The drama of the dosing ritual

Participants described the dosing session as highly choreographed, with meaning attached to small gestures, objects and transitions.

This was seen as especially important in psychedelic sessions, where participants may be more sensitive to the environment than in ordinary clinical encounters.

“And I suppose that the sort of key thing about the experience, the dosing is just, I guess, I'd really encourage you to think about, like the choreography of it, the drama of it, in terms of like, every gesture, every aspect has meaning. So it's like just being I mean, personally, I think that in trauma informed trauma informed therapy, therapists should have that level of awareness anyway, about the meaning of every aspect of the room and how you behave with the patient. But generally speaking, in therapy, people aren't necessarily thinking about, you know, where should I put the chair? Or, you know, at what point should I close the door? Or, if I'm giving them a prescription, how should I do that. But in this setting, you need to be attentive to all of that, because it all has symbolic meaning, particularly once the individual has then taken the dose. So I found it very interesting, for example, that the psilocybin capsules were given to me in a in a really beautiful little carved wooden bowl. So again, it's like, this is not popping a pill, you're not opening a little kinds of boxes or bottles that you'd get from pharmacy, this was, you know, felt like there was a nod to spirituality.” (P3)

The participant's reflection on the dosing ritual highlights the potential for such deliberate acts to invoke a sense of spirituality and intentionality. This attention to detail can enhance the therapeutic encounter, creating a context that fosters a mindset open to the transformative possibilities of the psychedelic experience.

### 3.2.4. Food during dosing

In the eating disorder study, food during dosing was discussed as a meaningful part of the session rather than a routine practical detail.

The participant described being invited to approach fear foods differently during the session, which they experienced as part of the therapeutic work.

“My guides were just extraordinary, I still feel really kind of overwhelmed and emotional thinking about them actually. Because they were they were just they were so amazing. But I did feel like they were sort of preparing me I guess for what their role was going to be during that that whole situation. And I think for me as well because my trial was for treatment resistant anorexia, food was involved in in the dosing as well and which is possibly slightly different to other people. I had brought, you know, I was encouraged not only to bring grounding objects, but I was also encouraged to bring some small food items that were maybe slightly more challenging for me or that might I might perceive to be sort of fear foods. And as the as the trial went on, the things I challenged myself with were increasingly more challenging because I felt more able to do that and actually ate those foods during the dosing while I was still.. it was extraordinary actually, and quite a remarkably healing experience with the food during the dosing. And obviously, my guides were very involved in, in helping with that and fetching the food or. So they weren't just sort of passive companions, there was a level to which they were involoved. But again, so sensitively done, I guess, just not being directive, not telling me what to do. Just being there to help me sort of, you know, work out what what direction I was going in.” (P3)

Another participant, P2, recounted the joy of tasting berries during the session, suggesting that even simple foods can acquire an extraordinary quality and appeal in the psychedelic state.

“I had lunch during, they gave me some berries, something really basic for those berries and the best berries I've ever had in my life.” (P2)

“eah, it's not I think by and large the feedback we get is you know like the beauty of like blackberries and like the fractal nature of the even the beauty of like sweet fresh fruit yes is what I think a lot of the psychedelic trial participants really value.” (P1)

“I ordered off the canteen menu, my lunch and I ordered something and suggested the veggie option not even recommended but she was so spot on. So just having something like really light and in DMT trial I had and because of my dosing ran late and my my food was kept on the on the canteen counter with a plastic lid dehydrated and that was just horrendous. There weren't very many options. It was a chicken breast, which looked like it could bounce off the plate.” (P1)

Contrastingly, P1 recounts a less favorable episode where the food offered post-dosing failed to meet expectations, both in presentation and choice.

### 3.2.5. Minimising disruptions

The imperative to minimise disruptions during psychedelic dosing sessions is a theme of important concern for participants, as it directly impacts the continuity and depth of their experiential journey. Participant P4 articulated the intrusion of having importants checked hourly, a necessary but potentially jarring procedure that, while important for safety, momentarily detaches participants from their immersive state.

“In terms of disruption, I was brought out every hour for my vitals, which was not always disruptive but I was wondering if that's still happening in the trials these days?” (P4)

“You can be in another universe kind of thing, and then you sort of get tapped on your arm. And you know, I was at a great point and now you're waking me up and checking the pressure on this kind of thing. You can sink back into it afterwards but still.” (P4)

“And then I suppose the other thing I kind of want to say about this is you may also need to be really mindful about how to support the individual, like if they need to go to the loo. That was a really big thing for me. So obviously, you have a level of control over over the room itself. So they've completely transformed this room. But this was a room in a clinical research unit. So outside of that room, there were people walking around with kind of nursing uniforms on, it wasn't a hospital, but it had a very clinical look to it. And I had to use the bathroom, at least twice, I think, during the course of my dosing, which meant they had to escort me out of that room down the corridor to a bathroom. And, you know, obviously I was still very heavily under the influence of psychedelic. And that entire experience was quite interesting, quite overwhelming in some ways. I have lots of experience of being an inpatient in hospital, both psychiatric hospital and medical hospitals. So certainly, I think on my first dosing, when I first had to go to the loo, I had a real horror moment where for a minute, I thought I was back in hospital in psychiatric hospital, I thought I've been admitted. So I was a bit freaked out. And they had to kind of just really like, almost like form a little... I don't know, I can't explain it but remember that both therapists like, but like on either side of me just protecting me and shielding me, they encouraged me to kind of keep my blindfold on. So I was sort of trying to like shuffle down this corridor. And obviously, key thing as well, they had a special piece of like cardboard that they, they went into the bathroom before me to block the mirror, which is like essential. So again, just these all these protective measures that they were trying to put in place to help me not feel too disturbed by, you know, seemingly, you wouldn't normally think anything of it, a patient going to the loo and individual going smooth during during that consultation. But this is a big, big deal as a part of the experience.” (P3)

“I had to take that horrible sterile walk into this clinical brightly lit white. Yeah, yeah, it was it was tricky.” (P2)

The journey to the restroom presents a significant shift in setting that can disrupt the participant’s state of mind. Participant P3 vividly recounts the delicate navigation and the essential attention to detail required when escorting participants, such as covering mirrors to prevent jarring self-confrontation and maintaining a protective presence.

Overhearing discussions among the clinical team emerged as a source of inadvertent disturbance, with Participant P1 highlighting the anxiety induced by overhearing logistical concerns.

“And one of the things I overheard the clinical medical team, sort of thinking about how far back they can push the screening and I thought it was quite anxiety inducing because I realised they had a window within which they had a dose, I think it was a two hour window. So I was overhearing these panicky conversations about how long this headache will last. So I'm like, "okay, maybe it's fine. I'm sure I'm fine. I'll be fine. Like, geez, you can't really go through that experience of this kind of headache. This is not going to be fine", you know. But I really feel enough pressure for the sake of science to like not have a headache that lasts longer than 120 minutes, right? Because it'll influence influence dosing and science and the whole world will fall apart because I have a headache. So that is that I think if that conversation happened out of earshot, it would have been helpful too.” (P1)

“I think my biggest learning in this is about communication. I actually on my first day, which ended up being the placebo, woke up with a really severe migraine. And so I was given a little bit of extra time to try see how that would play out. And so the clinical team and the doctors were really supportive and saying that they couldn't give me any pain relief. And you know, just see how we get on. But I think the the there was a lot overhead, a lot of conversation, quite a bit of pressure to try and get over this migraine, about how long you know how long things need to be out of the fridge? And what happens if we go beyond that window? And what happens if the first IV line doesn't work and how quickly it needs to go? Because it obviously there's a lot of science in how quickly things are administered, how long the extended state lasts. And so I guess, on some level, I felt a little bit stressed about this not being ready on time and being headachy. And can I get over it? And actually, it all worked out really well. Because all truth be told, I probably I mean, I then agreed to it fully. No one forced me into it, but my headache hadn't gone. And actually, it was a placebo, which was fine. And then I could have some pain relief afterwards.” (P1)

“And the importance of not overhearing these clinical discussions during dosing.. it was hard enough having these before. Obviously, those conversations need to happen, but behind the screen or out of the room.” (P1)

Caffeine withdrawal was discussed as another example of how ordinary physical factors could affect the dosing day.

“At the same time, I have to say, I mean, it's not, it's not, I'm not blaming anybody because it was such a new trial. But it did feel like You know, if things hadn't been maybe thought out or planned correctly, I mean, it was only sort of minor things. And there was little disagreements between.. you know, I was told, for example, not to have any caffeine that morning or anything that sort of psychoactive, which was fair enough. And come around about one o'clock in the afternoon, I started feeling really drowsy. And asked if I could have a, you know, a coffee or a tea or something. And there was sort of, you know, one person was saying, yes, you know, he can and the other person was saying, No, I don't think he should. Right. So if there was little times like that, and I thought, well, you know, maybe that should have been sorted out beforehand kind of thing. Because I got the impression it was just sort of two different ideas of what what should happen. I also got very hungry, and, you know, I pointed this out, and they didn't really want me to eat anything. One person didn't. And the other person said, I should you know, that that kind of thing. But I think that was because it was well, you know, it was one of the early ones that probably learned from those kinds of things now. What Yeah. So preparation, I would say overall, very good. But you know, a few little things might needed ironing out.” (P4)

“And similar to P4, I'm not around caffeine, but actually my migraine was caused by caffeine withdrawal, actually. And then I didn't drink caffeine after that is like "I'm not going to do that again", because it was intense.” (P1)

Unexpected events, such as music failure or alarms, were seen as difficult but not always avoidable. Participants emphasised the importance of calm and skilled responses from facilitators.

“The key thing isn't that you can't mitigate against every possible disruption never ever happening. Like surprising. Like I said, I think I gave the example of random in the headphones, cut out the music, stuff like that shocking, you know, completely bizarre thing to happen. It's how you then how the therapist skill to deal with it mitigates the level of damage, you can't... You can't deliver this, this treatment in a hermetically sealed box where there's not any disruptions whatsoever.” (P3)

“Or, in my case, something that happened during one of the doses is that the music suddenly cuts out, which was terrifying. I felt like I'd been literally dropped into a dark well, I was right in the depths of my of my journey, and the music just stopped. And it was some sort of issue with the Bluetooth connection I was having these headphones on. And, you know, it never happened before. You know, they they spoke to me later, where they would be, I think they were mortified that that happened. Remarkably, they fixed it really quickly, and they were just super sort of compassionate and competent to help me, you know, I was so confused about what was going on. So, you know, things happen. And that might not be ideal. And I just think it's about people feeling prepared to, you know, it was obviously a sort of alarming situation for them, but they managed to keep their stress levels down for me, they've managed to kind of deal with it in quite a practical, swift way. And to help me come back to a place of like, comfort and safety again, and totally understood exactly how frightening it would be in my shoes for this music to suddenly disappear.” (P3)

“On the point about dealing with the unexpected: I had a fire alarm while I was just post-peak at this point, but I was still very much in the depths of it. And my two guides handled it impeccably, one guide left the room to figure out what's going on and came back and said everything's fine. And speaking to her after the trial, you know, she had like genuine concerns. "So how do we get you walking out if we need to evacuate the building now and where's the assembly point and how do I get you to an assembly point". So, I mean, there was some genuine concerns for them but held so safely like I just looked at them and they said it was okay and I really felt it was okay because it was portrayed in a calsm way. The fact that the one guide left to go find out was also assuring because then you know, somebody's on it, I don't have to do this thing. It then it went off a second time, at which point I thought the universe is just having a laugh. I actually then had fits of giggles at that point that the ridiculousness of the situation.” (P1)

“Yeah, yeah, similar with me. But that was one of them was kind of busy trying to get the music again. The other one was always with me, it was just like, "Okay, we're working on it", - like, you know, just exactly how it's how you support the person in the context that disruption, but the key thing, so it's not just what to do, or like what to avoid, but when it comes up how to deal with it.” (P3)

## 3.3. Optimising dosing

### 3.3.1. Desire for multiple doses

The participant’s account emphasizes a substantial engagement with the psychedelic experience, highlighting a yearning for further exploration and healing within a structured trial setting. Despite not encountering visual phenomena, the emotional depth of the experiences, beginning with a low dose and escalating in intensity with subsequent higher doses, signifies the potential for incremental therapeutic impact.

As time progresses, the participant's eagerness to participate in another trial emerges not solely from a desire for repeated doses but also from the holistic therapeutic scaffold that trials provide, including integration sessions and support structures that facilitate the processing of these substantial experiences.

As the months have gone on, I've been really keen to get onto another trial, but mainly because it's not just about the doses, but everything that can go hand in hand with that in terms of integration, the access to support around helping to process that. For me, the desire to do it again is a feeling like there's more work to be done. And I don't feel able or have the resources to do that on my own. And I'm even more convinced that traditional mental health services aren't the most impactful route for me. So that's my motivation to want to do it again. (P3)

The participant reveals a discernment that the path to addressing their needs may lie outside conventional mental health services.

### 3.3.2. First dose as preparation for a second dose

Participants described trust as developing over time. Some only recognised after the session how much trust had still been missing at the start.

For another participant, the transition from the first to the second dose was important; permission to freely express themselves without fear of judgement or embarrassment allowed them to fully immerse in the experience, moving from a state of resistance to surrender. The reassurance provided between sessions—symbolized by the encouragement to accept whatever arises, even to the point of wetting themselves—proved to be critical in alleviating fears and inhibitions.

“There's something that one study clinician, who again, I was very blessed to have in the room, as one of my guides, said to me, probably about a year after. And he was like, Oh, you didn't trust us after the first session, or before the first session? You didn't trust us? And I thought, you know, I'm really, I felt terrible that that had come across, you know, and I was just like, oh, no, that's you did all you could, but he was probably right, you know, took that first session, and then the conversation between sessions for that, really that vital trust to be there. So I went from resisting to letting go. And I think that's why the first trial worked for me really?” (P2)

To be given that permission is vital for me. During my first dosing, I was frozen on the bed, didn't feel I could roll over, I was worried about wetting myself, embarrassing myself somehow. Thankfully there was a second dose. “Whatever comes up, just do what you have to do”. And I moved so much on that bed, I didn't get up and dance or anything, but foetal I was, not worried about how I might be judged or perceived by people in the room. And the increasing trust helps with that. They really helped me (by saying) "wet yourself, it's fine", and I didn't, but it was like getting rid of another bit of resistance for me. “I can't let go fully, because otherwise, what might happen?” (P2)

Together, these accounts suggest that trust may develop gradually across preparation, dosing and integration, rather than being present from the first session.

### 3.3.3. Support person pick-up post-dose

The participant's experience brings to light the logistical and emotional intricacies that accompany the post-dosing phase of psychedelic trials. While the insistence on a companion to facilitate a safe return journey highlights the rigorous safety protocols in place, it also inadvertently highlights the participants' dependency and potential inconvenience caused to their support systems.

For the participant, the situation posed a challenge but also presented a serendipitous comfort. The tube strike, although disruptive, offered an unexpected resolution by necessitating an overnight stay at the facility.

“I'm just thinking. So my first dose. My wife came to pick me up at the study site. And they insisted that somebody came to meet me, and took the taxi home. My second dose, which was the 25 milligramme dose of the high dose, I'm so glad there was a tube strike, which meant I had to stay on site overnight, the night before and the night after. And then I made my own way home, the day after the high dose.” (P2)

The account also shows how practical safety requirements can create emotional and logistical demands for participants and their support networks.

# 4. Integration

“I think tried to start making sense of my experiences really, really quickly, on the dosing day, as the effects began to wear off, I did want to talk about it, I did want to sort of share with them things that I had seen and experienced. And in some ways that was I think that was helpful and it was all part of the process.” (P3)

Participants reflected that integration—a critical component of the psychedelic experience—commences as soon as the dosing effects start to subside. For them, integration wasn't a separate phase but a continuation of the dosing day.

Another participant noted the internal pressure to ensure the experience was fruitful, which led to a feeling of urgency to "do a good job" in remembering and making sense of the journey. The guides played a important role here, balancing active listening with reassurance, emphasising that it wasn't necessary to force comprehension in the moment.

I felt a lot of anxiety about “what if I forget all this? What if it washes away?” And the sitters were just trying to reassure me “you don't need to make sense of it right now, we will come back to this and have an integration discussion the following day”. I think my tendency to want to work hard was good but was also coming from a place of anxiety and feeling pressured. They were quite good at reassuring me: “do whatever feels right, you can do some writing when you get home if you want to…” I was given some advice about things I may want to do. (P3)

Additionally, the journey home was seen as an integral part of the integration process. The structured and supported transition back into the everyday environment—such as a companion to accompany them and a taxi ride home—was a further extension of the care received during the trial.

“But I think it's quite interesting to sort of the like, I guess the conditions and the way in which the person is supposed to go home after the dosing, that's part of integration to my mind. So the me part of the plan was that my partner was going to come to the centre, would meet me and go back home with me in my taxi, which they paid for which was great. I barely spoke to him on the way back and again, I was reassured that you don't need to talk to him about it. Like I think I was trying to keep myself in that protective bubble. And when I got home I spoke to him a bit but I also was still quite in my own world, my own experience. I think I did a bit of writing to sort of jot down some stuff, but It still felt quite resonant. But yeah, we've been given advice beforehand about you know, avoiding social media and not getting immediately onto my phone. Not immediately communicating. being very mindful if I did want to watch TV being super mindful about like, what I expose myself to. I guess the advice I was given is that the real world might feel very, very jarring, a bit alarming. So be cautious and protective of yourself about what you're exposing yourself to in that that evening of the dosing. And I did follow that advice. I think it was good advice.” (P3)

## 4.1. Processing changes and integration over time

Participants described psychological changes that included reduced resistance, greater openness and shifts in how they understood their symptoms.

Changes in Depression

For one participant, depression came to be understood as resistance to thoughts, feelings and emotions, and the session provided a different experience of relating to these states.

One participant recalled being asked whether they felt depressed and realising that, at that moment, they did not.

I remember the question being posed, "are you depressed?' and I was worried about killing the fairy… I paused for quite a while… "No, I don't feel depressed" and that was amazing to be able to say that. (P2)

One participant detailed a personal revelation that psilocybin assisted in dissolving this resistance, facilitating a journey through darkness that led to a space of less restriction and greater openness.

“And that's key for me and I, I do see my depression as resistance now, really, largely, it's about trying not to feel something not so think certain ways not to be, you know, always trying to, yeah, you're constantly rigid, and you're pushing, you know, and psilocybin really helped me to kind of lose that resistance or to go through it, go through that darkness. So there was there was no tolerance issues with it for me on that. So it's, it's amazing. And that's why this research is so important, because look at mine and P4's experiences, they're so different.” (P2)

“My general trajectory with all of my psilocybin experiences has been like resistance, terror, fear, like during the second trial. Yeah, resistance really which is what I now see my depression as, its resistance to thoughts, feelings, emotions, and the more I push, the tighter I am within that grip, the more brittle and restrictive it is.” (P2)

The recountings illustrate a consistent pattern across the experiences—a pattern of initially facing resistance and fear that is emblematic of the struggle with depression.

I felt some lasting impact from the experience for quite a few months afterwards. It felt like the psilocybin was showing me a whole new journey of healing that I could go on. And although it had been incredibly profound and in some ways transformative in the short term, I had the sense that it was the beginning of the end of a journey, a useful window into how I might be able to work on the difficulties I have. But then the frustration and disappointment rushed in because I thought: “well, how do I do that?” It was just such an interesting, alternative approach to self-healing, with potential for continuing to be useful in that respect. (P3)

I felt a lot of grief at my trial ending because I trusted my guides so much, I felt very connected to them, they had accompanied me in the rawest expression of my emotion, they had witnessed aspects of myself that I just don't think other people have ever seen. So the trial coming to an end was quite a painful thing for me. They were cognizant of that. Part of my intention setting for my third dosing was: “This is the last one right? Think about letting go”. We were talking about it, I think that's the key thing, Things were still painful, it was hard, but I just I needed help to give a sense of ritual to the ending and closure. (P3)

The single most important piece of advice that I wasn't given, and it's been one of my key takeaways, is that this is the start of a process, and certainly not the cure. (P1)

“And the reason it didn't feel like therapy to me is because they were guides. I didn't see them as therapists, they were a supporter walking alongside me here, not offering a cure, or a therapeutic intervention. They were guides, facilitators, supports, alongside me, people who have maybe a bit of a sense of the territory, they have a map, but they you know, they're not telling me where to go or what to do. There was no expectation, this wasn't going to be a big trip, a life transforming trip, "this is your one chance".. this was just a process that we were engaging in. And it's a process that starts a thing. It's the start of a process. So for me, like my psychedelic experience now is about an ongoing relationship, and a further deepening with this, you know, the same kind of guide, almost like an apprenticeship or lineage, you know, you become part of something much bigger. So, and I think it started with that clinical experience. And I think what it's given to me, is that confidence to embark on on that sort of personal healing journey.” (P1)

We were speaking to somebody who was really struggling with the rate of change and equated it to crash dieting. It was such a massive change that they weren't able to integrate that degree of change. So resources and support addressing this are necessary because it is not a slow and steady process. I think for many of our participants, having support integrating the rate of change would be really helpful. (P1)

“After my last days, which was on the underground, whatever. I, I still haven't written about it. I always very much was I mean, it's like P3 was saying earlier, by writing it down. It's so you can connect to it. Great. But it's also because so you can grasp at it along the line. So this time round system was in April, I haven't written about it at all, I haven't formed a narrative around it really, because it was very much about just like be an hour you need to be in the moment, experience life as it is. Don't keep going back to that point, take what you've learned from it. Be that, live that? It's I don't know. It's a bit of an experiment, really. I do think journaling or teaching writing has always been a key way for me to express myself. So I do think it's really important, but I do think it's given me a little more freedom this time in some respects. And I had a conversation with a friend a few weeks back. And he was very much doing that. Oh, what about this? What about that? And I was like, I don't know yet. You know, and that's kind of the way we need to keep it. It's not in a box yet.” (P2)

“But I think it's quite interesting to sort of the like, I guess the conditions and the way in which the person is supposed to go home after the dosing, that's part of integration to my mind. So the me part of the plan was that my partner was going to come to the centre, would meet me and go back home with me in my taxi, which they paid for which was great. I barely spoke to him on the way back and again, I was reassured that you don't need to talk to him about it. Like I think I was trying to keep myself in that protective bubble. And when I got home I spoke to him a bit but I also was still quite in my own world, my own experience. I think I did a bit of writing to sort of jot down some stuff, but It still felt quite resonant. But yeah, we've been given advice beforehand about you know, avoiding social media and not getting immediately onto my phone. Not immediately communicating. being very mindful if I did want to watch TV being super mindful about like, what I expose myself to. I guess the advice I was given is that the real world might feel very, very jarring, a bit alarming. So be cautious and protective of yourself about what you're exposing yourself to in that that evening of the dosing. And I did follow that advice. I think it was good advice.” (P3)

“But yeah, I mean, I went back to my room was able to speak to my wife and that was that was great. I watched some nature programmes. I think on the little TV that was in the campus room. And that really helped me. It kind of makes sense because there was a session the day after. So I didn't have to travel the ages. You know, there and back just 24 hours later, but as P4 says, with integration, I had the playlist that they let us have access to and I listen to that pretty much daily. And yeah, the the journal that I'd written about it, or when I only really wrote about my high dose I think a week after that by going back to that I was able to kind of reconnect to those faces and places within myself, which really helped.” (P2)

Because there’s a lot of questions from all sorts of people, but where do you begin? “How do you think it’s helped you?” Because I was sort of midway through CBT at that time, which wasn’t going very well. And it was just endless questions from the CBT therapist, “what happened, how do you think it’s helped you in this?” (P4)

One of the best pieces of advice someone gave me was to not talk about it too quickly. And to feel I was getting permission not to answer the questions. Choose who you share your story with very carefully. That was incredibly useful advice. (P1)

“One of the things I want to just mention, I suppose, which is is something that I appreciated, while the trial is still going on, I was being given permission to not.. let me be clear, the way my trial was, I'd have a dose one day, and then the following day, we'd have to go back to the centre. And there were a few practical things that helped. They got me a taxi, which was a bit of a luxury because, you know, I could have got there myself, I live in London, it was, you know, would have been like an hour's journey. But they got me a taxi, which meant I could just still be very much in my own little safe bubble. And the team were very direct about saying, "Look, because when I had to go back the next day I had to have an EEG, yeah. And I guess I kind of often feel the pressure to like, make conversation and be really sociable, and like, oh, "how was your weekend?". And they were really good about saying, "Look, you don't have to talk to us", like you are allowed to just do what you want in this space. We'll be faffing around, doing the things, but if you want to chat to us, that's fine, but don't feel there's a pressure to, and that was so relieving, so I could just still continue to be tapping into how I was actually feeling.” (P3)

“I took annually leave in order to attend the visits and integration days. I believe, yeah, my line manager knew but I said I'm on a clinical trial without going in detail. I was quite cautious with who I talked about It.. mainly because I found that I was more likely to get a kind of baffled, baffled reaction, like, oh, you know, I think most people just think, "Oh, you're just going off and taking some drugs." I just I think it's I don't think we're quite, you know, in my experience, weren't able to quite make the connection between how strongly therapeutically impactful it could be and I was, I also didn't want them to burst my bubble by just, you know, I've had experiences of trying to describe my experiences and people just sort of visibly glazing over and that's also a bit for something that had been so incredibly powerful for you to see people just looking at you're like, "Oh, God, here we go. Yeah. You were a cheetah, okay." And it's like no, you don't really don't know what that means to me and how it felt. So was my cautious. I regret going back to work. My dosing was on a Thursday, I had integration on Friday but I also went back to work on a Monday and I really regret doing that. I think it was too soon. I remember those Mondays at work, just feeling like just not ready to be there, still really wanted to be connected to my experience, feeling like I was really in a different place. I've got loads I could say about integration, by the way, but are we carrying on?” (P3)

“And you might have time that you might want to take off as well, right, just the act of touching on it. You know, I kind of wish that someone had said to me, we could have said something along the lines of "other participants have found that going back to work on the Monday is a little tricky. And you may want to you know, it's not pushing people I just said you may want to consider actually, if you have the means at work, taking it a bit more, a couple more days, just to let you process...” (P3)

All I wanted to do was connect with nature and just feeling.. feeling the softness of the ground beneath my feet, feeling the breeze on the skin, my face. It was amazing. A dog running around… it's such a beautiful experience to do that.. Just looking at the clouds and just really being in that hyper present state still. In that whole moment of experiencing and fully feeling every emotion. (P2)

“This idea that was like post session or for psychedelic experience that you'd bring in kind of regresses to a kind of childlike state I didn't do in the session. I've always found that interesting. And I don't know whether there's anything to it or not, but I found myself watching kids shows, reading kids books and that kind of thing and loved it. Absolutely loved it. Scaredy Squirrel. thoroughly recommend.” (P4)

“Yeah, couching it in the neuroscience and actually saying, you know, you're in this, you are going to be in this period of increased psychological flexibility and neural rewiring to find these new ways of being, really opening that.” (P1)

## 4.2. Importance of support during integration

### 4.2.1. Not enough support

Participants felt that some studies ended with too little structured integration support. This was especially difficult when experiences were confusing, benefits were short-lived, or participants wanted to keep working with material that had emerged during dosing.

“The integration side on the one trial wasn't very good, I don't think. We didn't get any kind of sort of like you know, be careful of this, be careful that.. it was just more "you might feel a certain way, you might feel a bit confused the next day." Just the basics really. What's interesting about not not using social media and you know, being aware of what you're watching kind of thing, I think that would have been useful, you know, to say the people beforehand. And afterwards it didn't really feel like there was either there was much in the way of contacting them back you know if there was any issues that arose or, or anything like that. I felt very much like strangely enough when a bird first learns to fly, you're out you know, go and kind of deal with it. It was unusual, but it brought about experiences in of itself really. Because I wasn't given much guidance, and that sort of finding my own footing, which was good and bad, far that. I think the the partners need to be more involved with that, you know, if sort of if it's explained to them, you know, this might happen, or this is what's that may be like or, you know, the kind of things to expect because she really had very little idea about, you know how it was going to be when it came back.” (P4)

“I've heard from some other some other participants who had a really really tough time with things afterwards. You know, they were saying "I felt like I'd got to a point and I wanted to explore that further", and they were quite rude to me when I requested that I may be having another sort of treatment or a session in this kind of thing. They were told quite bluntly "No, that's it you know, you're aware of this" and but I think there's you know, that was what was 2015, so I think a lot of things have come on since then, by the sounds of it, but yeah, the integration could clearly be better. I don't know what P2 thinks about that, but it's certainly how I percieved it.” (P4)

“Yeah, the with the pilot study, there really was no onus on integration at all. So that said, they were, they were always available and relatively responsive via email. And kind of I could really share my dissent freely by my emails that I sent to them afterwards, you know, saying, Yeah, what do I do? How do I kind of keep hold with this? Yeah, and then eventually, how do I get another dose? You know, they're like, well, we can't give you that.” (P2)

“For myself, it was it was difficult, because as P2 was saying, I've been at the one trial. And after the trial had finished, I sort of met them the day after, it was all very much sort of like, this is goodbye kind of thing. Which was sort of nice, in a way, you know, because of this kind of thing. But it felt like that was it. And that from then on in I was, I was on my own, kind of thing. They had sort of given me a few hints about you know, how to deal with things going forward, but the next day, I was kind of disoriented anyway.” (P4)

“General points is more than more than we received would have been great. And I think also, you You know, those those two, I know they were pub meetings, but imagine if we'd had a group like that monthly? Yeah. Imagine, you know, that would have been so valuable.” (P2)

### 4.2.2. Reassurance and guidance

Participants valued reassurance that there was no single correct way to integrate the experience. Guidance was most helpful when it reduced pressure and supported participants to find their own workable practices.

There’s not necessarily a right or wrong version of integration. I found some writing useful, but I also danced, I used my photography a lot, went on walks.. There's all sorts of different things. And I think just encouraging and empowering the person to find out what feels intuitive and natural for them. The last thing you want is to turn it into a piece of work, you don't want to feel like set homework to do, because then you just stopped being engaged with it in the right way. (P2)

“But they knew that I felt I think I felt a real pressure for it to be useful. And I think the sort of the little bit in me that wants to do a good job and wants to like work at it and be a perfectionist and achieve and like all this sort of stuff, you know quite quickly was kicking in or, gosh, I must make sure that I remember all this and I felt a lot of anxiety about what if I forget all this? What if it washes away? And the sitters were just trying to reassure me you don't need to make sense of it right now. We will come back to this and either have an integration discussion the following day, and I think, you know, my tendency to want to really work really, really hard, was was good but also I think was coming from a place of anxiety and feeling pressured. So, again, they were quite good at whilst they listened, whilst they were very keen to hear about what I had experienced, and we're certainly active in that discussion... and I'm talking at the very very end of the dosing day, I think they're also quite good at just reassuring me like you don't have to.. do whatever feels right, you can do some writing when you get home if you want to.. I was given some advice about things I may want to do, but I was also very much encouraged, like, "don't feel pressured to try and actively hold on to this, trust that you'll hold on to the bits that you need to".” (P2)

### 4.2.3. Working with disappointment

Disappointment was discussed in relation to low-intensity experiences, perceived non-response, or return of symptoms. Participants described how this could feel like personal failure, particularly when they felt invested in the study and the team.

I'd been going through the difficulty, the darkness and going in and through that to the other side, being hyper aware and hyper present, but with my depression and anxiety coming back as I came back into the room, I was just disappointed. I just felt really uncomfortable generally, but I also felt that perfectionism and trying to get things right, I felt like I failed. I had this whole team of people piling that effort and energy into me to help me and it hadn't. My mindset at that point was it hadn't worked. (P2)

“But my biggest problem with the you know, the integration afterwards was that my second dose didn't have the, the effect, because they've been so close together. I felt like I'd missed out. Even though I'd had a great experience on the lower dose, it was absolutely fantastic, and it was life changing. They kept building up the "this is going to, you know, this is gonna be really strong, and it's, you know, this is where you're going to learn the most". And so I was quite disappointed in that, and I contacted them about it and said, "Look, is there any chance I could come back and have the proper second dose?" And that wasn't allowed, and that kind of thing, s I found a bit left down there” (P4)

“It's pointless for me to talk about how the second dose was, there was just hardly anything there, I didn't feel anything. I just remembered, you know, way back in my sort of the teen years, when I'd had experienced psychedelics then, I remember the, the old sort of guy of the group, you know, the most experienced one. He was saying, you know, take all you want, you know, it's this is non addictive. Because, you know, within a week, they have no effect kind of thing. And I don't, you know, I don't know whether that played any part of it. But I was very disappointed in the second one, and, and some of it was because of the preparation between the two. The preparation was, oh, this is going to be so powerful. You got to prepare yourself. And, and it was a total anti-climax. And the study lead, did say in a meeting again a year later, that they changed that, they weren't going to do the week in between, because there was somebody else that had, you know, a negative experience on the second dose as well.” (P4)

### 4.2.4. Reframing disappointment

Follow-up sessions could help participants reframe disappointment without dismissing it. The most helpful responses allowed grief, frustration and uncertainty, while keeping open the possibility that the experience might still matter over time.

This is where the session the day after was so important.. I don't think I'd be here talking about my experiences now without that session... it was a psychiatrist who really helped me to reframe it. He was like: "Last time you got a gift, you got an afterglow, and that was great, and you enjoyed that. This time, this is where the hard work begins." And I had been reminded of the insights from last time and I have new insights to work with from this time. It's very much like: “I didn't get what I wanted, but maybe I got what I needed”. And the session afterwards helped me to embark on a different therapeutic path by recommending some counsellors to me that dealt more with somatic work rather than trying to solve the problems of the mind with the mind. The team were vital in helping me with that. (P2)

“after the second trial, there was a lot more onus on integration. So we had the sessions afterwards, which were vital in me not seeing the second trial as a complete failure. You know, so that was huge. I think that's where integration really began was like the day after.” (P2)

### 4.2.5. Appropriate study ending

Ending the study could bring grief, loss or anxiety, particularly when participants had built strong trust with facilitators. Participants recommended that study ending be prepared for explicitly, rather than treated as an administrative endpoint.

The single most important piece of advice that I wasn't given, and it's been one of my key takeaways, is that this is the start of a process, and certainly not the cure. (P1)

“I'm gonna just add one other thing, which I guess slightly complicated, but I think at with, as with anything in this, it's also just always checking in about the reason why, why someone's asking because I can imagine a situation where if I didn't, I didn't do this. But if I have said to my therapists, or can I see that video, we want to see it, they would have said, let's explore why, because I was having quite a hard time accepting the whole thing to come to an end, grief was quite a big thing. And actually was, I was very paranoid and anxious that I was going to forget everything. And I was really worried about oh, what if this doesn't work, because I basically forget everything. And for me, asked him to watch the video would have been in the interests of I need to memorise this, I got a hold control, in a really controlling, grasping way, like, it went definitely would have helped me connect compassionately with myself as well, that would have been an added bonus. But if I'm honest with myself, it would have been very much in the interests of must remember, on the right note? Oh, yeah, like 12 to 3, I started dancing, you know, would have been that kind of Yeah. And actually, something I had to come to terms with is you've had the experience, trust you're gonna hold on to something. Like, there's something about the letting go process as well. So I just think it's important that therapists have, you know, a sort of sensitivity around, let's explore the reasons why let's, let's understand this is going to be helpful for you. We don't want to, we don't want to kind of collude with a less helpful urge. We want to kind of help this to be as here the therapeutic as possible, perhaps the healing thing for you as an individual is not seeing it? And is” (P3)

“It is really difficult. I knew I knew when my last session, we did a process. Yeah, I mean, it's, yeah, don't ya know? Because it didn't make it any easier. Knowing when that date was, it was like, Well, I've kind of made these deep bonds with these people. Yeah. And yeah, they were still accessible via email here and there. But it was, yeah, I wasn't at all prepared for that, for that disconnection.” (P2)

“Yeah, you have to form a deep bond for the treatments work properly. So you can let go fully, and, you know, surrender to it, really. And allow yourself to be that vulnerable. But yeah, I mean, by virtue of that you've built this trust with a team of people that are just gonna disappear out of your life, potentially. If they were going to be properly boundaries, they probably should. Yeah.” (P2)

“If it comes in the preparation stage, we're talking about need to turn inwards, this wisdom in your own healing and you can watch the ending and that is all forgettable. And you will turn inwards and you will be resolved. Give me things and that's again, the same message.” (P1)

“It's a difficult one. I felt like, I didn't need anything afterwards. And it was sort of a sad goodbye kind of thing. Yeah. And it's gonna be different for everybody. Totally understand that. But I felt I've benefited more from just been caught off point, kind of a sharp one. And because I felt massively confident, either I continue with anything. I just felt great afterwards. But everyone's different. So I mean, it could be, you know, the next if I did add another guy. Yeah, different dose. Different socials, or whatever.” (P4)

### 4.2.6. Locating agency in the participant

A recurring point was that support should help participants locate agency in themselves, rather than in the study team or the substance alone. Integration tools were valued when they supported self-trust and ongoing practice.

“If it comes in the preparation stage, we're talking about need to turn inwards, this wisdom in your own healing and you can watch the ending and that is all forgettable. And you will turn inwards and you will be resolved. Give me things and that's again, the same message.” (P1)

“I suspect, as well, I think the difficulty, and I wonder whether certain trials have gotten better at this language, is that I think, certainly on my trial, the sort of narrative was very much around, "this is your journey, we are here to help guide you know," ultimately, even with integration, and I was given a lot of support with integration, but even with that, you know, the onus really is on you, sort of taking responsibility within that, I suppose. And it's not at all to say that you or others didn't do that, because I felt like you weren't supported well enough to do that or to be to feel that you were in a safe enough place to go on that journey.” (P3)

A major learning for me from the trial was that "The answer is within you". It's not another medicine or another doctor or another therapeutic approach - the answer is within. And being given the tools to be able to work with that within yourself; whether it's journaling prompts, playlists, or body based practices. (P1)

I was given a lot of support with integration, but even with that, the onus really is on you, taking responsibility within that. If you've been someone who's had years where everything you were indoctrinated to believe was: “The solution to your problem is in a service or medication or therapy”. It's a big shift to think, "oh, gosh, no, the answer is in myself" That's massive. (P3)

## 4.3. Optimising integration

“So with that was really things I did myself, you know, like spending time in nature, writing, listening to the playlist, every day, pretty much meditating, that sort of stuff, things that didn't work before. The trial now worked really because I wasn't so desperate for them to work. I have that sort of spaciousness and lightness” (P2)

### 4.3.1. Guided journaling

Journaling and guided prompts helped some participants record experiences, return to insights later, and continue the integration process after formal study visits had ended.

I was given an integration booklet to help me as a resource that I could use if I wanted to, to help with the integration process. It has various kinds of questions that I could try answering to as a kind of journaling guide. And it inspired me to keep a journal, but I was supported in that process with the excellent integration resource that the team had produced. (P3)

I wasn't encouraged to keep a journal, but I did. And it was so valuable during both trials. Going back to that was similar to going back to the playlist to go back to the dosing days, and being able to go through that process again, and to be reminded of the insights and the connections, the epiphanies, that experience was so valuable. (P2)

“I mean, the other thing that I think, you know, my trial did did really well is I was given this booklet at the end, it was a small booklet, was it the end or actually might have been no, it must have been when I did my first dosing, because it was effectively a series of journal prompts. I was told that I could use it if it felt helpful. I also could completely ignore it. But it also have like little quotes from people like Jack Kornfield, Ram Dass, yeah, Ram Dass, just little things like that, which was actually really helpful because they were sort of subtle. Like, if you want more of this, this is where you can find it.” (P3)

“They said, here are some integration groups you might find useful. I am sure they told me about PsyPAN. But I also did you know, again, because this booklet had these little quotes, I, you know, went off and like, got it you know, and heard of "After the ecstasy the laundry"? , I got that audiobook and started watching lots of films about psychedelic experiences. I was really trying to keep the whole thing alive. Yeah, obviously listen to the playlist they gave me. But I'd also created my own playlist. And I listened to that a lot. I did use the booklet they gave me, I did a bit of journaling.” (P3)

### 4.3.2. Drawing and visual expression

Drawing and other creative materials were useful when experiences were difficult to put into words, especially after highly visual or non-verbal experiences.

“We weren't given any sort of integration booklets or anything, it was just sort of verbal things afterwards, but we weren't given, it's actually part of the trial requirements, we were offered the opportunity to draw our experiences after the DMT experience, which was actually really useful in terms of integrating some of the experience.” (P1)

“I had the same guide, one of my guides on both trials, so quite similar in some respects, but on the DMT trial, which is really lovely, they introduced a lot more materials during the on site integration sessions. So you know, they were odd materials and really getting into doing a lot of reparenting and getting to inner child stuff. And having a box of crayons is like entirely liberating and tried to draw draw things but it's not being pressured because you don't want to having to produce a masterpiece.” (P1)

### 4.3.3. Listening to the playlist and guided recordings

Participants described study playlists, guided breathwork and facilitator recordings as ways of reconnecting with the experience after dosing.

In terms of integration afterwards, on my own, I would listen to the playlist, a lot. Got back into music more. (P4)

“Yep. So they provided that they gave me three recordings. And it was one it was it was the guides that are involved in the trial. And in each recording, they it's like a kind of guided, meditative bit of breathwork with a poem. They're beautiful actually. So each one had a different poem, and a bit of guided breathwork. They last maybe three or four minutes each. And I've still got them. They're something that I can go back to if I wanted to They were given to me, I think, in the run up to the doses as well. But again, it was just these really interesting techniques to help me stay safe and calm and grounded.” (P3)

“And then the only other thing I want to do to say is that what's very powerful, I suppose about some of the tools that you'll might be given along the way is that you can continue to go back to those. I've still got the recordings of these breathwork exercises. One of the kinds of key resources I was given was a poem as well. So there was a poem that they read to me at times, which was just incredibly beautiful. It was all about finding the Warrior Within yourself and overcoming fear and overcoming anger, and it just all kinds of really powerful stuff. And it was read to me at various points during preparation and during the dose, pre-dosing. And at the end of the trial, I was given a copy of it. So again, it's something I can go back to, it's a material object that I've still got access to, in the same way that I still got access to the playlist. And given that there can be grief that comes up at the end of these trials, having something which gives you that that link back is is really helpful.” (P3)

### 4.3.4. Access to recordings or summaries

Some participants wanted access to recordings or summaries of dosing or integration sessions, especially when they feared losing important material. They also recognised that this would need careful boundaries and support.

“This is subclause to this. And I think I did mention it during our conversations is the what would have really helped me with to, to have had access to the session that I had the day after, when I came in. And yeah, I was in a state where I was grappling and trying to die. This hasn't worked for me, how do I make sense of if I still feel like the day after I was in a bit more of a kind of open state, and I would have really benefited from having access to the recording they took of that session? And they said... Yeah, they recorded it. And I asked them afterwards. And they were like: "No, there's no way we're not allowed to share that with you". And it's like, well, if I'd known that I would have put my own phone on the table in front of this and recorded it myself. If allowed.” (P2)

“I mean, this was more this was more about the day after that initial session that I found, because things closed in and closed down very, very quickly, particularly because I've got my secondary guide's cold as well, like everything was dead again quite quickly. But yeah, having access to that there was still I mean, again, maybe I'm grasping and clinging by saying that, but having access to your side, having access to yourself, you know, having access to sort of yeah, having access to that. Other materials as well. You know, they are using as part of your data Okay, audio recording of these things. Exactly. Yeah, that's that's all i all i wanted really was like access to that recording. So I could draw some strands from that and go, Okay, well, from the integration, we can focus on this or but and, you know, it wasn't allowed to, you know.” (P2)

“I just want to say I would definitely say that my craving for the recording was born out of desperation and clinging, but understandably, I was because "it hadn't worked" inverted commas. But I think even bearing that in mind, I think it would have benefited me. Yeah. So, it's a tough one.” (P2)

I think if patients are going to be given permission to view the video, it should happen in a therapeutic setting, with a therapist to work through it together and shouldn't be given ownership, not to take home and potentially post on Instagram. Plenty of people might want to do that and maybe regret it later, particularly when people are sharing things on social media hoping for a positive connection and it not going well. (P3)

“I'm gonna just add one other thing, which I guess slightly complicated, but I think at with, as with anything in this, it's also just always checking in about the reason why, why someone's asking because I can imagine a situation where if I didn't, I didn't do this. But if I have said to my therapists, or can I see that video, we want to see it, they would have said, let's explore why, because I was having quite a hard time accepting the whole thing to come to an end, grief was quite a big thing. And actually was, I was very paranoid and anxious that I was going to forget everything. And I was really worried about oh, what if this doesn't work, because I basically forget everything. And for me, asked him to watch the video would have been in the interests of I need to memorise this, I got a hold control, in a really controlling, grasping way, like, it went definitely would have helped me connect compassionately with myself as well, that would have been an added bonus. But if I'm honest with myself, it would have been very much in the interests of must remember, on the right note? Oh, yeah, like 12 to 3, I started dancing, you know, would have been that kind of Yeah. And actually, something I had to come to terms with is you've had the experience, trust you're gonna hold on to something. Like, there's something about the letting go process as well. So I just think it's important that therapists have, you know, a sort of sensitivity around, let's explore the reasons why let's, let's understand this is going to be helpful for you. We don't want to, we don't want to kind of collude with a less helpful urge. We want to kind of help this to be as here the therapeutic as possible, perhaps the healing thing for you as an individual is not seeing it? And is” (P3)

“I think it's the majority of the people, it's driven from that place? And I would say that the middle of what I'd said most people, because control is such a thing in depression. So I think most people would be driven from that place. And maybe the middle ground is if you're your guides, you know, this comes back to consent communication, or whatever, maybe they can send you some bullet points, or what you're thinking, during the session, you want to do an integration. During the session, you felt that some body based practices really good, you want to do X, this was really like for you, I don't know, they're just adds words to them. It could be just something that, again, that person could take away.” (P1)

### 4.3.5. Returning to practices that previously felt unhelpful

Some approaches that had previously felt ineffective, such as journaling, movement or nature connection, became more accessible or meaningful after the psychedelic experience.

“I think it's a bit of both, you could have tried these things beforehand, you could try journaling, or dance or any movement practice. And, you know, I think the receptivity is definitely, I'd even say to somebody who may have tried this before, yeah, might have tried five talking therapies, six SSRIs. That can work. But actually some of some of the activities you might have tried before, you might be more receptive. And they might actually work differently for you.” (P2)

“Like earlier, I said, you know, afterwards, like being out in nature, I wasn't so desperate for nature to work for me, because it was already in a better place. You know, journaling. It wasn't just this really long, sort of depressing piece of writing or doing in morning, that really helped me to express myself. Meditation, again, I wasn't grasping so much. So. Yeah. Yeah, completely. Like, if you desperately try and bring something out of it, it won't work for you. That's the problem, isn't it? Yeah.”” (P2)

I think bodywork, dance, physical movement. Which I'm uncomfortable with generally, but that's a path that's opened up to me since the second trial and has really served me well. (P2)

“More useful, because they're working with my depression, or my health generally, I guess, on a level that is actually working way better for me than just trying to resolve the problems of the mind with yet more thinking. I kind of feel like I've done that. And then it's like, alright, well, let's get on now dealing with what's here. And that seems to be bringing a lot, you know, sort of more comfort and safety, and you know, peace really. Not that there are a lot of any of those things generally for me still, but they helped me to tap into it. So yeah, doing EMDR has really helped doing cranio-sacral therapy.” (P2)

### 4.3.6. Resources for sustained integration

Participants wanted practical resources at the end of the study, including integration groups, reading, playlists and contacts for further support.

### 4.3.7. Continuity of care after trial participation

Participants described a gap between the depth of the study experience and the care available afterwards. Psychedelically informed follow-on support was seen as preferable where available.

“So yeah, but actually, the idea that there is something that people are given at the end of the trial with resources for further integration.” (P1)

“They said, here are some integration groups you might find useful. I am sure they told me about PsyPAN. But I also did you know, again, because this booklet had these little quotes, I, you know, went off and like, got it you know, and heard of "After the ecstasy the laundry"? , I got that audiobook and started watching lots of films about psychedelic experiences. I was really trying to keep the whole thing alive. Yeah, obviously listen to the playlist they gave me. But I'd also created my own playlist. And I listened to that a lot. I did use the booklet they gave me, I did a bit of journaling.” (P3)

“We're building the database, and there are a couple like IPT has a psychedelic integration therapists database actually building that. And then maybe for trial participants, and having people that are finishing, they do like psychosynthesis training, and they need client hours, they can do like reduced rate, low cost counselling.” (P1)

I started seeing my therapist after the first trial, and having the experience of going into the DMT trial and then going back into that therapeutic support is a huge improvement for me in terms of my integration and the support I've had. So I think that will be really important, some way of discharging people into something. (P1)

So, the stipulation from our trial was that I had to be going back to therapy or going into therapy afterwards... it was good, but it was also odd, because I had this experience on the trial, and then started a very traditional, typical, same old thing I've had before, an eating disorder therapy at an eating disorder service. And I was like: "God, you know, wow, this really is not the way to do it". And she was nice, she didn't stigmatise my experience, but I would much prefer to have been working with someone who was more psychedelically informed. (P3)

### 4.3.8. Briefing the support network

Participants felt that partners, family members and other supporters often needed simple guidance about what the participant might go through and how best to support them.

What is really important is support for families, something to give to them to help them support you during your integration process. You may not have the words at that point, and they may not know what to do. Something tangible that they can check to support you, without you having to say what support you need, would be really helpful. And I think planning in some activities, so children feel part of it, especially if there's a massive degree of change. It's like, "Well, mom's like a different person. What's going on right here?" I certainly started eating differently post-trial, really taking better care of myself and my body and just helping them with that too. We should focus on this as a sort of journey for the whole family. (P1)

“I think just explaining the process to say, you know, that you'll be away for a little bit of time, you'll come back, you'll you know, you might be a little bit of space, a bit of quiet time, and then actually even planned some family stuff. We had some really gentle evenings with, like, like soft movies and cosy blankets and cuddling up together, and just actually just feeling the family unit. And yeah, they were happy to do that, I think planning in some, some activities around almost to help them on the journey. Right. So they feel part of it, especially if there's a massive degree of change. Yeah. It's like, "well, mom's like a different person. What's going on right here?" I certainly started eating really differently post trial, really taking better care of myself and my body and just yeah, just really helping them with that, too. So we had lots of berries and fresh, yummy things, and dark chocolate and yeah, and some ice cream. So yeah, just really focusing on it as a sort of journey for the whole family.” (P1)

“She was absolutely over the moon to see that, you know, the depression had gone, but some of the things I was trying to talk to her about I felt frustrated with because you know, you can't it's ineffable, a lot of the experiences, you can't really talk about it but you feel like you want to. Yeah, so yeah, I think a lot better integration, good integration is absolutely vital.” (P4)

“I think the other thing is, it's, I think I mentioned it before, in terms of your family group and your source of support around that. I didn't know quite how to phrase it to my partner. Yeah, what had happened, I mean, she saw the positive changes, which was great. But other than that, you know, I think it would be better for, for somebody on the trial, to speak to partners to say, look, you know, if this happens, maybe try this, you know, give them some advice as well, kind of thing for yourself, so they're better prepared for when you return.” (P4)

# 5. After the study ends

## 5.1. Challenges after study ending

### 5.1.1. Anxiety about returning symptoms

Participants described anxiety and distress when symptoms returned after a period of relief, especially when no further access to the study intervention was available.

“But yeah, I think it is a very important thing, the integration and I think without it, without it being proper, people can have such a hard time because they're sort of, they've seen their way out of the problem. And then when that starts to sort of subside, then kind of stopped out, get back to where they were.” (P4)

“I think, Well, Andy, was, really, I mean, when I spoke to him, he said, "Oh, we're going to, I'm going to force it, I'd really have to do this again, and if I don't, then I want to, you know, I'm gonna have to cause problems for them" . And he was even at one point talking about about legal things, because, you know, he reckoned they messed his mind up so much kind of thing, then he wanted them to, to, to solve his problems. So yeah, we know this. Everybody had a different way, I suppose of dealing with things based on their experience, per se, my main one was that I didn't have the effective second dose. So that kind of felt like I missed out on it.” (P4)

### 5.1.2. Desire to access the study intervention

Participants discussed wanting further access to the intervention or to redosing, not simply because of the drug effect but because of the full support model around it.

“And I think that you're moving towards redosing, you know, like understanding that this isn't a once and done. And what's really interesting in the integration space is actually having some kind of way of measuring where people feel that need is coming up for them again, and they get a peer support model, you can support each other. And arguably, if you have peer support, you might need redosing later, or you might stay better for longer than having to need the dosing sooner because you know, you are supported on some level.” (P1)

### 5.1.3. Finding a therapist for further integration

Participants often struggled to find therapists who understood psychedelic experiences and could support integration after study completion.

“And then the other thing that I did, I got pretty desperate for some one-to-one, integration support, and I found a woman again through the psychedelic society who quite uniquely has experience of eating disorders and psychedelics and she lives in South Africa. Do you know her? Francesca?” (P3)

“Yeah, she did a panel talk for psychedelic society and I watched it, I had COVID at the time, a little bit out of my head, and I basically emailed her saying, "Help me". And I had some sessions with her, which I paid for, again, that was quite uncomfortable for me because it was quite a lot of money. But they were really interesting, we sort of, you know, we use dancing as a way of kind of integrating things. And again, it was useful just to talk someone who really got it. But yeah, it was not was not necessarily practically viable, I would have loved to continue working with her if I could afford it.” (P3)

“Were you given at the end of the trial any kind of how do you how do you say psychedelic sympathetic counsellors or whatever were you given any resources as far as that goes, because the only reason I got referred on the such by, after second trial was because I asked, I was like, "Do you know anyone that works in this area that does more body based work that will be open minded about psychedelics?" because you hear about people, they finish a trial, and they have to deal with the stigma of, or people that just don't understand or an existing therapists they go back to, and they just don't get it or they don't agree with it.” (P2)

“An idea we've had is that somehow the sector subsidises people like that. So post trial you can get, yeah, that's fair access to the therapeutic support that people need.” (P2)

“We're building the database, and there are a couple like IPT has a psychedelic integration therapists database actually building that. And then maybe for trial participants, and having people that are finishing, they do like psychosynthesis training, and they need client hours, they can do like reduced rate, low cost counselling.” (P1)

### 5.1.4. Seeking other psychedelic studies

Some participants looked for other trials after their study ended. This was framed as a sign of unmet care needs rather than as a simple desire for repeated trial participation.

“I've looked into all sorts of things, I've looked into whether I could join on the trials. I don't think I'm really eligible, like my trial was very specifically for anorexia. I don't have a diagnosis of treatment resistant depression, although I do struggle with depression, like I don't fit that most of the trials are focused on depression or increasingly PTSD. So I've looked at if I could find a trial, I've looked for therapists. I've self-medicated like I've really looked at every possible... I even pay for a bit of additional therapy, very, very short term with this woman in South Africa, I really tried lots..” (P3)

“I really wanted to take part in another trial. But the problem that I saw was That's because it was treatment resistant depression, that I'd have to go to my GP and he would have to say, well, you know, part of the criteria was either to have been on antidepressants and and had the talking therapies. Well, that's already demonstrated to have not worked, I've tried all of that. So if I went back to my GP, and you know, the criteria for taking part in the trial would be that I'd have to try those things again. No, no, they don't work. So I can't see a route to take part.” (P4)

“But the urge to do that indicates right. I agree with you that the solution is not "Oh, how can we provide even more trials for these people, so keep just going through more and more trials?". But I think the urge to do that is saying something about the treatment model? Yeah, my personal feeling is, is that if, you know, if you are going to roll out psychedelics as a treatment approach, I think what would have been helpful for me is, I had three doses in January, I think it would have been good to then have a repeated another three doses, say, like six months later, and not like, I'm doing the trial again, like, if that was going to be a treatment model, I think that would be, you know, with really thorough, comprehensive integration as a follow up, that, you know, operates over the course of a month or two, maybe even longer, so that people are just kind of being supported on their journey. With a constant focus on self healing, that is what you're always working towards, is.. getting to a point where you don't need anything else at all, but it's just an incredible the difficult place to get to if you're someone who has been in such a vulnerable, challenging state, or who's had very long term contact with services.” (P3)

You've had this bright light shone on you and you're like: "Oh, finally, all the weights lifted", and then you feel it coming back, you're like: "Well, what can I do?" But you now know, something can help you. I'm not saying there's any comfort in that desperation of nothing helping you, but it's what you know, and now you've been given this hope. (P2)

### 5.1.5. Thoughts about self-medicating

Some participants considered or used psychedelics outside the study context after trial completion. They distinguished this from the study intervention because the legal, therapeutic and support context was absent.

I thought: “well, the only way for me to get that experience back is to self-medicate”, but I still felt like I needed the therapeutic support, somebody to talk to about the experience. (P4)

“But without the sort of therapy side of it, even though it was helping quite a bit, I still felt like I needed the sort of the therapeutic support, you know, somebody to talk to about the experience and that kind of thing.” (P4)

“I also took the decision to source my own mushrooms, again, that came with risks, that came with a lot of anxiety, because it's kind of goes against the grain to be doing that. And I was micro-dosing for a while, which I did find quite helpful. But again, would really rather be doing that legally, it just doesn't sit easy with me to be doing that in some ways.” (P3)

“I don't talk openly about what I've done, not on a stage anymore. So right here, I applied for the DMT trial that P1 was on. And I think I'm really glad it didn't get on that because the whole IV thing and the intensity of it all... but after that I was like, right, well, my choice is to, you know, I need this, and I need to find it. Luckily, by doing the work I've been doing, I do know people that I can sort of trust in this area to recommend other people. But, you know, again, it's not a path I want to be on, I don't want to be breaking the law. I just want to access the treatment that I know can help me.” (P2)

I thought: “well, the only way for me to get that experience back is to self-medicate”, but I still felt like I needed the therapeutic support, somebody to talk to about the experience. (P4)

## 5.2. Support after the study ending

### 5.2.1. Importance of peer support

Peer support was experienced as valuable because other trial participants understood the specific research context, including placebo, blinding, dosing, integration and study ending.

“I might have mentioned this before, but I was responsible for just administrating the WhatsApp group for the phase two of the one trial, which is how I first came across P1's name. Way back and yeah, the people that had received the placebo, they dropped out of the group within a week or two, generally, they didn't stick around, because you had lots of people. Yeah, I mean, it wasn't all about how wonderful it was. They were challenging experiences being shared in there as well. But if you hadn't had, you know, any experience at all psychedelic experience, it was very much, you know, people were welcoming as well. But I guess there was that feeling of the haves and have nots, you know, it's really hard if you're living with that disappointment anyway. So then hear about other people's experiences. I think it probably just, you know, made me put you in an even worse place. Sadly, yeah. So it's like,” (P2)

“I was just going to say that it was, you know, really, while it was challenging, I mean, P2, and I think that's how we first started talking, I said, you know, my husband can develop a website where we can have like, community chat, you know, you can have like people who had placebos, and then you can start screaming because that to find closure to something you didn't have was really, really hard. But what was really encouraging is that those people had actually joined the WhatsApp group that they were willing to even been placebo people. And, and kind of a bit opposite to what P3 was saying a while If you hadn't been on, you might not have looked for a psychedelic community. These are people who had been on didn't get the psychedelic experience, but we're looking for community. So I think when you don't have the experience, or you do get screened out, I do think people start seeking community, because then they start thinking, can I take matters into my own hand and how to do that responsibly. But what does that mean? And so I think, yeah, there's a need for community across the board.” (P1)

And then afterwards, there was an integration group… and those sessions helped me until COVID hit and then I stopped going. I desperately needed even more sessions, because I was lost and disappointed and really gutted. (P2)

“But yeah, at least there was an integration group to go to, an integration group, which was really helpful in many respects. It was very kind of, even at that stage, because that's 2019, some more people have been through the trials, they didn't tell anyone who... though I asked them "who here has also gone through the clinical trial?" and he said, "I can't tell you, they'll have to tell you themselves", which was very different compared to after the one trial, where we all met up for a drink a couple of times afterwards with the whole team. Ethically, I'm not sure which is best, where the boundary should be, but at least I had that. It was.. when people found out you'd been on the trial, there were a lot of questions and that sometimes felt like attention that maybe was difficult to deal with, in addition to being there to try and work through, integrating what, what I've experienced, etc. But that's human nature, I guess. But yeah, so at least there was that that strand after the second trial. I should add on that front, really that, you know, a facilitator and an integration-group facilitator, they set up their integration circle about two years later, 2017 I did go to the first one, but you know, it was quite far down the line and a bit late for me really. And the an integration group as well, both groups were set up by clinicians who knew that there was nothing post trial to support people, and they've all done that without being paid. Um, they just do it because I know there's a gaping hole there. And I think that's something that's really got to change, you know.” (P2)

I still think there is a value in having spaces for clinical trial participants, as opposed to having it for everyone and having experienced psychonauts there. That can work for some people, for other people it's a very different path that they've taken. And if it is a sector-wide responsibility, funded by various organisations, I think that would be for the best. (P2)

I think that the field is moving towards redosing and understanding that this isn't a “once and done” treatment. And what's really interesting in the integration space is having peer support, meeting others who have been through the same clinical trial process, which is quite unique and really important. You usually do not get this in an ordinary integration groups. I think there's something really important about the peer connection and finding the others. (P1)

Most of the people in that space hadn't been on a clinical trial, I was very much a minority. Most of them had a whole range of journeys and experiences with psychedelics. It was fascinating to start to uncover this whole other world. Finding community was really powerful. I met very loving and caring people who made themselves available and said: “contact me if you're struggling”. So that was amazing. (P3)

“Life last year got really intense, I got married, my mom got cancer, she then got very ill. I started doing new job, everything started to become a bit much. And I think I found it then increasingly hard to keep the whole thing alive. And I'm a real creature of habit. So what was helpful for me in the early days was I was going to certain groups, like weekly. And then I missed several for all kinds of life reasons, and then kind of found it hard to get back into it. And, and I think the thing that I struggled with, actually, and this is more of a personal anxiety, but as my, the time between the present and my trial got further and further and further, I think I started to feel less like I was allowed to go into those spaces, which is rubbish. But I think I was partly like, oh, but you know, it's been a while. Am I allowed to go back and keep talking about this?” (P3)

### 5.2.2. Involving the whole family

Where relevant, participants felt that family members, including children, could benefit from age-appropriate explanation and planned supportive activities during integration.

## 5.3. Optimising the post-study period

### 5.3.1. Support networks and continued integration

Participants recommended clearer post-study support pathways, including peer support, integration resources, psychedelically informed therapists and guidance for families.
